# *In Situ* Study of Electron-Tunneling through Dynamic Interface at Individual Nanoemulsions by Multimodal Single-Entity Electrochemistry

**DOI:** 10.1021/acs.jpcb.6c04932

**Published:** 2026-10-21

**Authors:** Anuradha Khatri Chhetri, Mandip Adhikari, Jiyeon Kim

**Affiliations:** Department of Chemistry, 4260 University of Rhode Island, Kingston, Rhode Island 02881, United States

## Abstract

To overcome critical challenges in modern drug delivery, lipid nanocarriers such as nanoemulsions (NEs) have emerged as superior drug carriers. While advancements in nanotechnology allow precise control over NE size and structure, the rational design of their functionality remains difficult. This is because functionality is primarily dominated by dynamically or statically formed interfacial structures that cannot be easily characterized using *ex situ* bulk analysis or electron microscopy. Herein, we demonstrate multimodal single-entity electrochemistry (MSEE)—combining amperometric SEE with voltammetry—to *in situ* probe electron-tunneling through the dynamic interfaces of intact, individual NEs. Using highly monodisperse NEs synthesized with varying concentrations of Pluronic block copolymer surfactants, we investigated interfacial electron transfer (ET) rates at discrete NEs. Notably, ET through the NE interface aligns with the predictions of the Marcus–Hush theory. We determined the effective interfacial thickness, tunneling-distance decay constant, ET rate constants, and transfer coefficient. This enabled angstrom-resolution differentiation of interfacial structures and structure-dependent charge-transfer behaviors across a series of NEs, independent of morphological size differences. Our MSEE approach provides a powerful electrochemical framework for investigating nanostructure–function relationships in lipid nanocarriers with mixed interfacial layers, ultimately aiding in the optimization of desired functionality such as delivery efficiency, release kinetics, and carrier stability.

## Graphical abstract

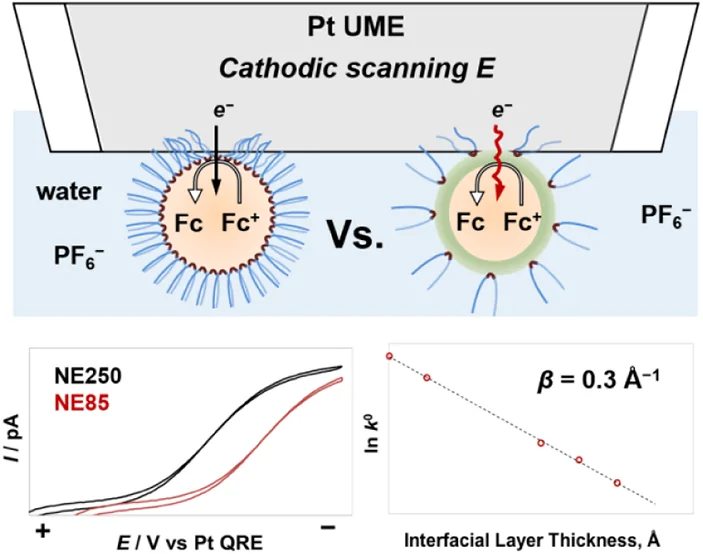

## Introduction

Lipids, essential components of biological membranes, offer unique physicochemical properties that solve critical challenges in pharmacological delivery methods, such as poor solubility, low bioavailability, enzymatic degradation, and off-target effects.^1^ Hence, lipid-based drug delivery systems have emerged as an enabling technology to enhance drug solubility, barrier penetration, controlled release, and biological protection in modern drug delivery.^1,2^ Among lipid nanocarriers like nanostructured lipid carriers, solid lipid nanoparticles, or liposomes, nanoemulsions (NEs) offer distinct advantages. NEs are fine oil-in-water (o/w) or water-in-oil (w/o) dispersions of two immiscible fluids.^3^ With droplet sizes ranging from 20 to 200 nm, they create a significant interfacial area for drug dissolution, improving the solubility and bioavailability of weakly water-soluble drugs, nucleic acids, and peptides.^3–6^ Because of the encapsulation capability of hydrophilic and hydrophobic drugs, NEs act as a flexible platform for diverse therapeutic applications.^7^ They can be formulated for oral, topical, intravenous, intrapulmonary, intranasal, and intraocular delivery.^8^ Furthermore, their ability to integrate new components with conventional equipment makes them cost-effective and easy to scale for commercial markets.^9^ Because of these physicochemical properties and adaptability, NEs have been explored as a core pillar of drug delivery applications.

Optimizing primary components such as surfactant systems requires the fundamental understanding of how surfactants dictate the structure, formation, and physicochemical properties of NEs. During oil-in-water contact, surfactants and cosurfactants create a complex mixed interfacial layer at an NE that can be either statically or dynamically formed.^10–12,21^ This layer controls crucial functions of NEs, including phase behavior, encapsulation efficacy, release kinetics, and stability. Because of this relationship between structure and function, modifying NE interfaces holds great promise for targeted drug delivery, particularly in treating malignancies and delivering drugs to more challenging systems in the body. However, traditional bulk analytical methods, e.g., mass spectrometry, UV–vis, IR spectroscopy, fluorescence microscopy, HPLC, and electron microscopy, are *ex situ* and provide only indirect or average measurements. They lack the nanometer-to-subnanometer resolution needed to directly understand these interfaces at intact NEs. Therefore, the field urgently needs *in situ*, direct, and individual-entity measurement techniques to deeply analyze intact NE structures that are further correlated with the unique functionality of NEs.

Single-entity electrochemistry (SEE) has proven to be highly applicable for *in situ* studies of individual vesicles, liposomes, microemulsions, and NEs due to its intrinsically high sensitivity.^13–17^ It is frequently utilized to determine the size distribution of droplets, structural differences, and partition coefficients, as well as to achieve ultralow detection limits in environmental and biomedical sensing.^17–20^ However, SEE based on conventional amperometric techniques relies on steady-state measurements. Consequently, it is limited in its ability to scrutinize the kinetics of interfacial electron transfer or resolve the intact structure of NE interfaces at subnanometer-to-nanometer resolution.

Here, we enhance the resolution of interfacial-structure studies at individual NEs by developing multimodal SEE (MSEE). This technique combines amperometric SEE, which monitors the trapping of NEs on a Pt ultramicroelectrode (Pt UME) in real time, with continuous voltammetry to *in situ* investigate charge transport across the NE boundary. Unlike an amperometric SEE, MSEE voltammograms yield rich information on interfacial electron transfer (ET) kinetics, transfer coefficients, effective layer thicknesses at the NE interface, and distance decay constants. Because our NE systems are inherently pinhole-free and highly monodisperse,^20–22^ the resultant interfacial ET can be treated as nonadiabatic ET, where distance-dependent ET is governed by the collective effect of mixed interfacial layers of NEs. This makes NEs an ideal, artifact-free model for applying Marcus–Hush theory to tunneling-dominated ET,^23,24^ thereby allowing us to evaluate MSEE’s analytical utility and correlate the interfacial layer thickness with the phase behavior of NEs.

In this work, we demonstrate MSEE to investigate dissimilar interfacial structures of a series of NEs by *in situ* probing electron-tunneling through a NE interface. This technique works by monitoring NEs as they are trapped on a Pt UME. Inside each NE is a redox mediator, ferrocene (Fc), which is instantaneously oxidized to ferrocenium (Fc^+^) upon NE’s collision with a Pt UME, producing a measurable anodic current spike. Immediately following this, voltammetry is applied to reduce Fc^+^ inside NEs via interfacial ET. Because of the high affinity between the hydrophilic group of NE surfactants^25^ and the inherently hydrophilic Pt UME surface,^26^ studying these trapped NEs as nanoreactors is technically feasible. Compared to traditional analytical approaches, MSEE offers several distinct advantages. It allows for the direct, *in situ* probing of concurrent charge transports across the interface of intact, pristine NEs in a nondestructive manner. Furthermore, it enables the application of well-established ET kinetic theories to correlate interfacial charge transports with the interfacial structure of NEs. Finally, the absence of pinholes on the NE interface minimizes experimental artifacts, thereby ensuring highly accurate and quantitative analysis of the electrochemical data.

We propose a novel strategy using outer-sphere electrode voltammetry coupled with amperometric SEE to characterize subnanometer-to-nanometer-thick interfacial layers within NEs as models of lipid-based nanocarriers. By using Fc as an outer-sphere redox mediator, we observe simple, tunneling-controlled ET between a NE and a Pt UME. Because Fc is sensitive only to the electrode’s density of states,^27,28^ resultant ET rates depend exponentially on the tunneling distance (i.e., the interfacial layer thickness). By adapting Marcus–Hush theory, we successfully determined the effective interfacial thickness, tunneling-distance decay constants, ET rate constants, and a transfer coefficient, thereby successfully distinguishing interfacial structures with angstrom-level resolution across a series of NEs.

## Experimental Section

### Chemicals

Pluronic F-127 block copolymer (F-127), bis­(2-ethylhexyl)-sebacate (dioctyl sebacate (DOS), 97%), tetrahydrofuran (THF, 99.9%), ferrocene (Fc, 98%), ammonium hexafluorophosphate (NH_4_PF_6_, 99.98%), and sodium chloride (99%) were used as obtained from Sigma-Aldrich. Potassium tetrakis­(pentafluorophenyl)­borate (KTFAB, 97%) was purchased from Alfa Aesar. Ferrocene methanol from Sigma-Aldrich was used after recrystallization. Nanopure water (18.2 MΩ·cm, TOC 2 ppb, prepared by Integral 5 from Millipore, US) was used for the NE synthesis and for solutions in all the electrochemical measurements. Isopropyl alcohol (IPA) was used for cleaning Pt UMEs.

### Nanoemulsion Synthesis

NE synthesis is reported elsewhere.^17^ Briefly, KTFAB (1.8 mg), F-127 (50–250 mg), and Fc (3.3 mg) were dissolved in 3 mL of THF solution. 8.8 μL of DOS was subsequently added, and the resulting mixture, i.e., THF cocktail was vortexed at 3000 rpm for 1 h. 0.1 mL of homogeneous THF cocktail was forcefully injected into 4 mL of nanopure water under vortex at 4000 rpm. The second batch prepared by the same procedure was combined with the first batch for a total of 8 mL solution, which was further homogenized at c.a. 5000 rpm (Polytron 10/35 GT, Kinematica, USA). 8 mL volume is particularly needed not to form foams during homogenization. Continuously, THF in the mixed solution was evaporated by N_2_ gas purging at a flow rate of 35–40 psi for 1.5 h to induce spontaneous emulsification. Finally, this was a NE stock solution (∼470 pM) ready for the characterization and MSEE.

### Fabrication of Pt UMEs

The fabrication of Pt UMEs is reported elsewhere.^29^ Briefly, a 1 cm long Pt wire with 25 μm diameter (PT00-WR-000114, 99.99%, annealed, Goodfellow, US) was inserted and positioned at the center of a 10 cm long pyrex capillary (9-000-1000, OD 1 mm, ID 0.2 mm, Drummond Scientific, PA, USA), and the entire composite underwent stretching, sealing, and pulling using CO_2_ laser puller (P-2000, Sutter Instrument, CA, USA) under vacuum (pulling parameters are in Table S1). As-pulled Pt UMEs were electrically connected with a Ni–Cu wire (160 μm, Alfa Aesar) and Ag epoxy (16014, Silver Conductive Epoxy, H20E EPO-TEK, Ted Pella) and glued with 5 min epoxy (5 min Epoxy gel, Devcon, OH, USA). As-pulled Pt UME was further polished to expose well-defined geometry and size of a Pt disk using a microelectrode polisher (MHK 1A, HEKA Electronic, MA, USA) with diamond papers (50-30150 (0.5 μm), 5030155 (0.1 μm) diamond lapping films, Allied, CA, USA). Polished Pt UMEs were immersed in IPA overnight for cleaning. Pt UMEs were vigorously rinsed with stirred water. To ensure the cleanliness of the electrode surface, Pt UMEs were cleaned with piranha solution before usage. To suppress electrostatic discharge (ESD) damage, Pt UMEs were handled with ESD-protection wristbands and stored on tip holders connected to grounding.

### Dynamic Light Scattering (DLS) Measurements

Size distribution, polydispersity, and ζ-potentials of NEs were measured using the DLS technique (Nanosizer Malvern Instrument). For the analysis, 2 mL of NE stock solution was diluted with 6 mL of nanopure water. About 1 mL of this diluted NE solution was delivered to a cuvette (DTS0012) for the estimation of the average size and polydispersity index. Continuously, ζ-potential was measured using a Y-shaped cuvette (DTS1070) filled with the diluted NE solution.

### TEM Measurements with the Uranyless Negative Staining Method

NEs were visually characterized by TEM at 100–200 kV (JEM-2100F, JEOL, MA). Uranyless negative stain (Delta Microscopies, France) was used because of the better contrast on organic molecules by staining a nonradioactive lanthanide mix. The specimen preparation is reported elsewhere.^17^ Briefly, 3.0 μL of NE stock solution was deposited on the carbon side of a 300 mesh Cu TEM grid with C/Formvar film (FCF-300, Electron Microscopy Sciences, Hatfield, PA) (NE suspension: 1.0 mL of NE stock sample was diluted in 3.0 mL of nanopure water). After 30 s of NE deposition, remaining solvent was wicked with filter paper. The grid was immediately immersed in a drop of uranyless negative stain for 30 s. The excess stain was wicked using filter paper, and the grid was dried overnight at room temperature and ambient pressure for imaging.

### Multimodal Single-Entity Electrochemistry (MSEE) Measurements

MSEE were performed with a two-electrode cell using a bipotentiostat (CHM8022D or CHM760E, CH Instruments, Austin, TX). For the sample preparation, 200 μL of NE stock solution was added to 10 mL of 10 mM NH_4_PF_6_ solution, vortexed for 15 min at 3000 rpm, and left on a bench prior to usage. Pt UMEs as a working electrode and Pt wire (250 μm dia., Alfa Aesar) as a quasi-reference electrode (Pt QRE) were used to perform amperometric SEE at 0.85–0.95 V vs Pt QRE and voltammetry (from 0.8 V to 0 or −0.25 V vs Pt QRE). Background voltammograms were obtained at the beginning of electrochemical measurements. For amperometric SEE, a constant potential of 0.85 (or 0.9–0.95) V was applied using the amperometric *i*-*t* technique, and currents were observed for 800 s. The continuous voltammetry was conducted by cathodic scanning of potentials, and these experimentally obtained voltammograms were corrected by background subtraction and further analyzed for voltammetric fitting with voltammograms theoretically simulated by COMSOL Multiphysics.

## Results and Discussion

### Characterization of Nanoemulsions

A series of NEs were synthesized with sequentially decreasing the amount of Pluronic F-127 surfactants from 250 mg to 50 mg in a 3.0 mL THF cocktail. These NEs were characterized by DLS, where the average diameter, zeta potential (ζ) as a measure of NE’s surface charges, and the polydispersity index (PDI) were measured (raw data in Figures S1 and S2). The PDI was 0.17 ± 0.05, indicating high monodispersity of synthesized NEs. With decreasing F-127 amount from 250 to 85 mg, the average sizes of NEs (denoted as NE250 and NE85) nearly maintain similarity (i.e., 40 ± 2 nm (*N* = 20, *N* denotes the number of replicate measurements) and 42 ± 2 nm (*N* = 20), respectively), while ζ potentials rise steeply to more negative values, i.e., from −17 ± 2 mV (*N* = 20) to −35 ± 2 mV (*N* = 20) (Figure 1A). With 50 mg F-127, the size of NE50 increases prominently to 56 ± 2 nm (*N* = 15) with a more negative ζ potential of −42 ± 2 mV (*N* = 15). Among these NEs, similar sizes but obviously different ζ potentials could imply the dissimilarity in their nanostructures. In fact, similar sizes and high monodispersity were visually confirmed by TEM measurements using uranyless negative staining method (Figure 1B).

**Figure 1:**
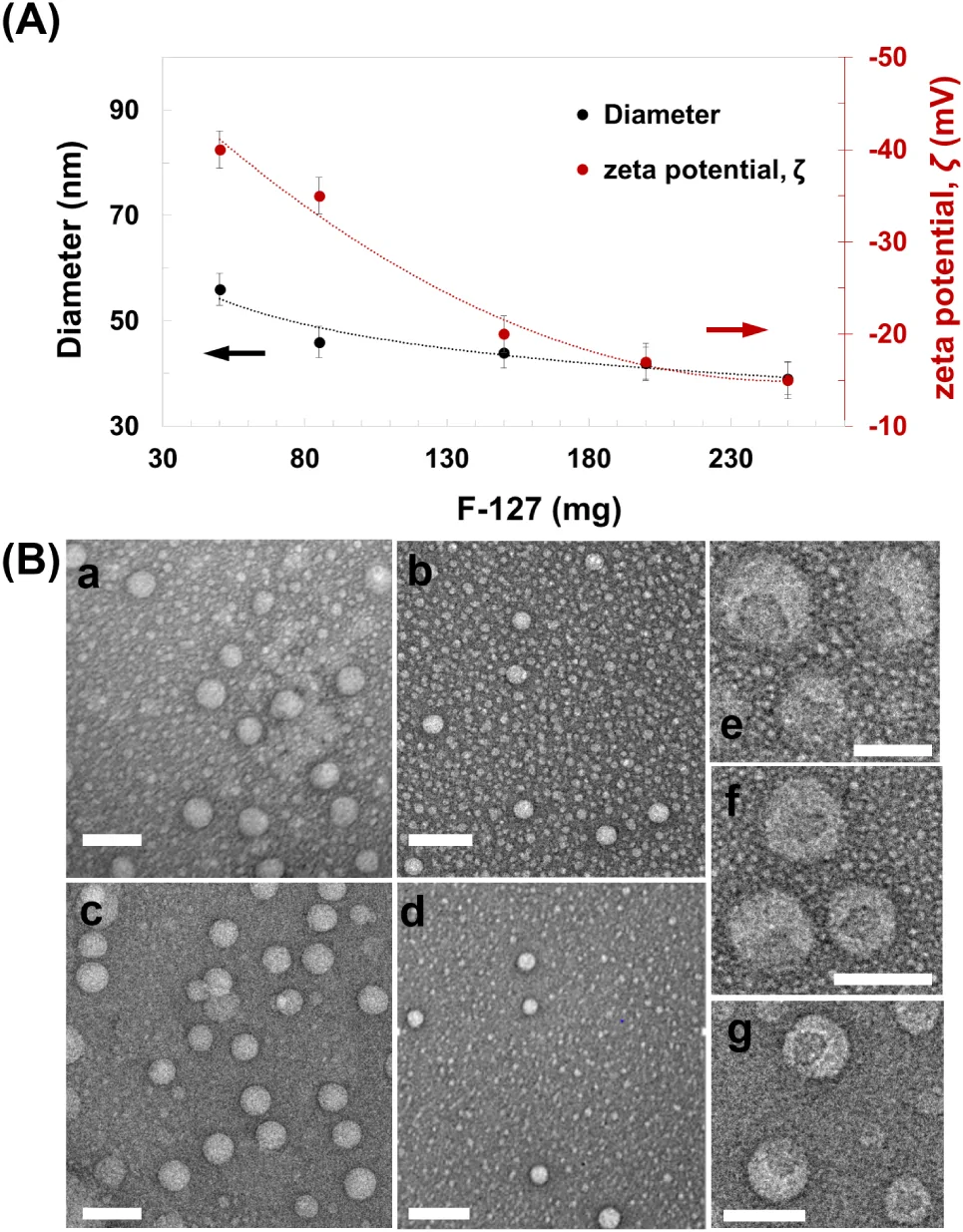
(A) Average diameters and ζ potentials of NEs as a function of F-127 surfactant amounts, as estimated by DLS. (B) TEM images of NE250 (a), NE150 (b), NE85 (c), and NE50 (d) obtained at 100 kV by uranyless negative staining with 100 pM NE suspensions. NE250 (e), NE85 (f), and NE50 (g) were cross-sectioned by the longer exposure of 100 kV electron beam, where no discernible difference was observed. Scale bars correspond to (a) 60 nm, (b) 100 nm, (c) 60 nm, (d) 100 nm, (e) 30 nm, (f) 60 nm, and (g) 60 nm.

Morphological differences, however, were barely discernible in the TEM images. While TEM provides valuable information regarding particle shape, size, and polydispersity, it offers limited insight into the inner or interfacial structures of NEs due to the intrinsically low contrast of their components.^30^ To address this, we increased the exposure time of a 100 kV electron beam to partially melt the NEs, aiming to uncover clues about their internal or interfacial configurations. Nevertheless, cross-sectional imaging revealed no noticeable structural distinctions across the different formulations, further underscoring the resolution and contrast limitations of conventional TEM for the NE samples (NE250, NE85, and NE50 in Figure 1B-e, f, and g, respectively).

Given the significantly different ζ-potentials observed despite the similar droplet sizes ranging from NE250 to NE50, we hypothesize that these NEs possess inherently dissimilar structures, particularly at their interfaces. For NE250, where the hydrophobic core is compactly surrounded by the hydrophobic poly­(propylene oxide) (PPO) blocks of the Pluronic F-127 copolymer surfactant, the two hydrophilic distal poly­(ethylene oxide) (PEO) chains of the folded F-127 extend into the aqueous phase, fully stabilizing the dispersed NEs (Figure 2, bottom left). In this configuration, the hydrophobic borate compound, TFAB^–^—acting as a cation exchanger within the NE—does not participate in structural stabilization and remains randomly dispersed inside the core. Consequently, the overall ζ-potential is primarily dictated by the electronegative oxygens of the hydrophilic PEO chains at the NE250 interface, resulting in mildly negative values.

**Figure 2:**
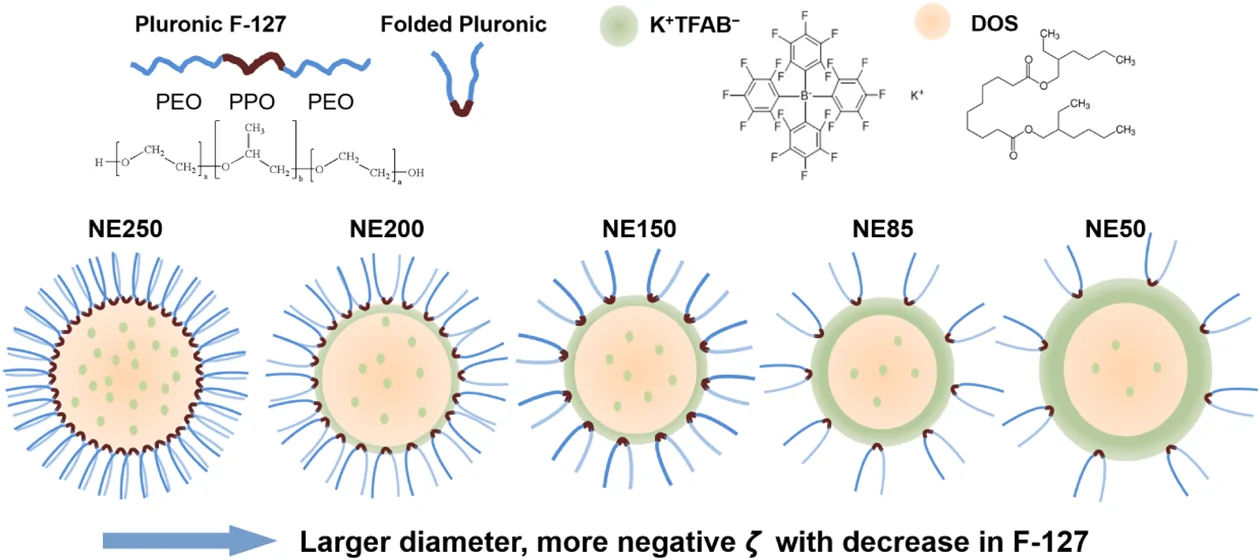
Schematic illustrations of components for NE synthesis and the hypothesized NE structures of NE250, NE200, NE150, NE85, and NE50 with dynamically formed interfacial layer of TFAB^–^ (green layer).

In contrast, for NE85 and NE50, which feature reduced amounts of F-127, the hydrophobic core is only coarsely covered by surfactants, leading to deficient steric stabilization in the aqueous medium. To compensate for this and provide additional structural stability, the hydrophobic TFAB^–^ anions likely align near the NE interface to minimize the high surface energy. Under these conditions, the strongly negative charge of the interfacial TFAB^–^ borate clusters heavily contributes to the total ζ-potential, making it substantially more negative than the baseline potential contributed by the PEO chains alone. Notably, TFAB^–^ still exists with K^+^ counterions, so the overall electroneutrality is maintained inside a NE. Our proposed mechanism for these dynamic interfacial structures across the various NE formulations is illustrated in Figure 2. Ultimately, a lower surface coverage of F-127 surfactants results in a thicker interfacial layer of hydrophobic TFAB^–^, which correlates with a gradual increase in the overall NE diameter (theoretical estimation of F-127 coverage on a NE is in Table S2). If the primary difference in NE structure lies in the dynamically formed interfacial or inner layers of NEs, neither TEM nor conventional *ex-situ* analytical techniques are suitable for accurately capturing these unique structures and their interfacial behaviors.

Previously, we used the amperometric SEE technique to not only study the structural differences between NE250 and NE85 but also test the temporal stability of NE250 along with DLS and TEM measurements over 2–3 days.^17^ That work revealed a borate (TFAB^–^) interfacial layer on the NE85, which acts as an electrochemical tunneling barrier, and the reasonable kinetic stability of NE250 over 15 h. However, this steady-state measurement, as well as conventional microscopy or other *ex-situ* analytical methods, cannot easily analyze the dynamic interfaces in these NEs. To address this, we developed a new electroanalytical method: multimodal single-entity electrochemistry (MSEE). We used MSEE to investigate a series of different NEs and experimentally validate our structural hypothesis.

### Multimodal Single-Entity Electrochemistry of NEs

We developed MSEE, coupling the amperometric SEE with voltammetry. This allows us to track the trapping of NEs under a Pt UME in real time and subsequently use the NEs as nanoreactors. We studied a series of NEs containing Fc as a probe redox molecule, monitoring dynamic interfacial ET.

During amperometric SEE, NEs in aqueous solution diffuse and collide with a Pt UME at the anodic potential for Fc oxidation. These discrete collisions produce immediate, discrete anodic current spikes, reflecting the continuous oxidation of Fc to Fc^+^ until all Fc inside the colliding NE is converted (Figure 3A). Depending on the thickness of the dynamic interface at NEs, ET across the NE interface can readily occur (black solid line arrow in Figure 3A left) or be mediated by electron tunneling, i.e., nonadiabatic ET (red curved line arrow in Figure 3A left). Consequently, the anodic potential required to completely oxidize Fc inside an NE must become more positive to overcome this latent tunneling barrier at the NE interface. However, the difference in anodic potentials across a series of NEs is insufficient to quantitatively map the dissimilarities in their interfacial structures.

**Figure 3:**
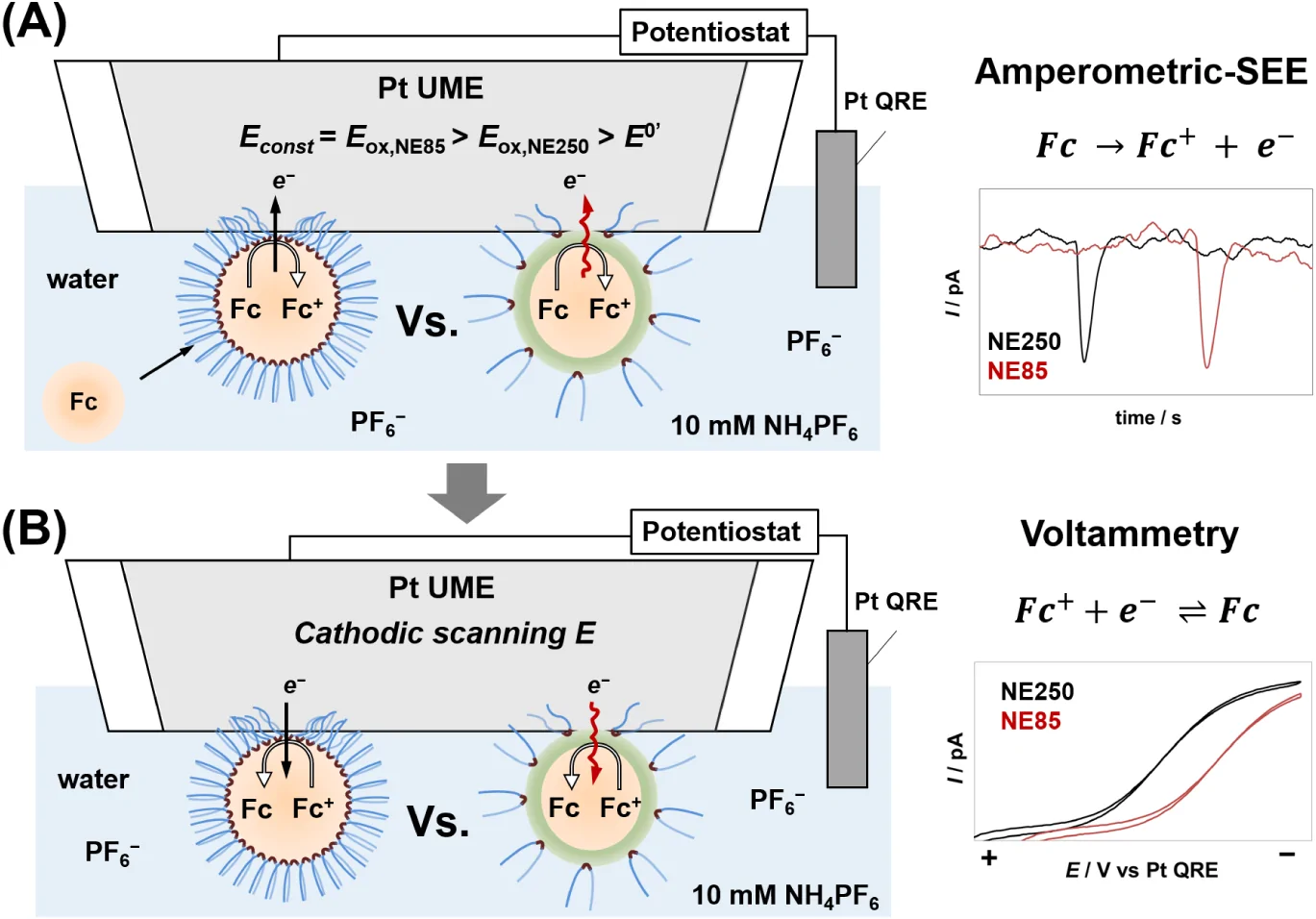
Schematic illustration of MSEE measurements with NE250 or NE85 dispersed in 10 mM NH_4_PF_6_. (A) Amperometric SEE is measured first, where NEs spontaneously diffuse and collide onto a Pt UME at the anodic potential (left), and anodic current spikes from Fc oxidation inside each NE are discretely monitored with time, i.e., *i-t* curves, to real-time track the trapping of NEs under the Pt UME (right). Depending on the interfacial structures at NEs, the anodic potential required to fully oxidize Fc inside a NE could increase more positively. For example, a thicker interface at NE (e.g., NE85) can retard the direct ET (black solid line arrow), thereby leading to e-tunneling-controlled ET, i.e., nonadiabatic ET (red curved line arrow). (B) Voltammetry is performed immediately after amperometric SEE, where electrolyzed Fc^+^ inside a trapped NE is reduced during cathodic scanning of potentials (left). The resultant voltammograms for Fc^+^ reduction are obtained via interfacial ET at each NE (right), of which shapes are governed by interfacial ET kinetics and the interfacial structures of NEs.

NEs can stick to the naturally hydrophilic surface of a Pt UME after collision and electrolysis.^26^ This attachment is driven by the hydrophilic distal groups of the PEO chains on F-127 that surround the NE.^25^ Trapping the NEs allows for continuous voltammetry and monitoring of interfacial ET during Fc^+^ reduction (Figure 3B, left). The resulting voltammograms vary from steep to sluggish depending on differences in the interfacial structures of the NEs (Figure 3B, right). These shapes indicate whether the process occurs via direct ET or electron tunneling-mediated ET, respectively. Compared with the current spike responses in amperometric SEE, this method provides much richer information about interfacial structures and ET kinetics. We employed this unique approach of MSEE to investigate NEs, including NE250, NE200, NE150, NE85, and NE50.

Figure 4A–E displays MSEE data for NE250, NE200, NE150, NE85, and NE50, respectively. First, a series of distinctly sharp current spikes with nearly uniform magnitudes were observed over time in the amperometric SEE. In amperometric SEE, an anodic potential of 0.85 V vs Pt QRE was applied for NE250, while those of other NEs ranged from 0.9 to 0.95 V vs Pt QRE. The 0.85 V potential for NE250 was selected based on cyclic voltammetry for Fc oxidation in a THF organic solution. At this potential, a steady-state current was observed, indicating that diffusion-limited ET was achieved (Figure S4).^31^

**Figure 4:**
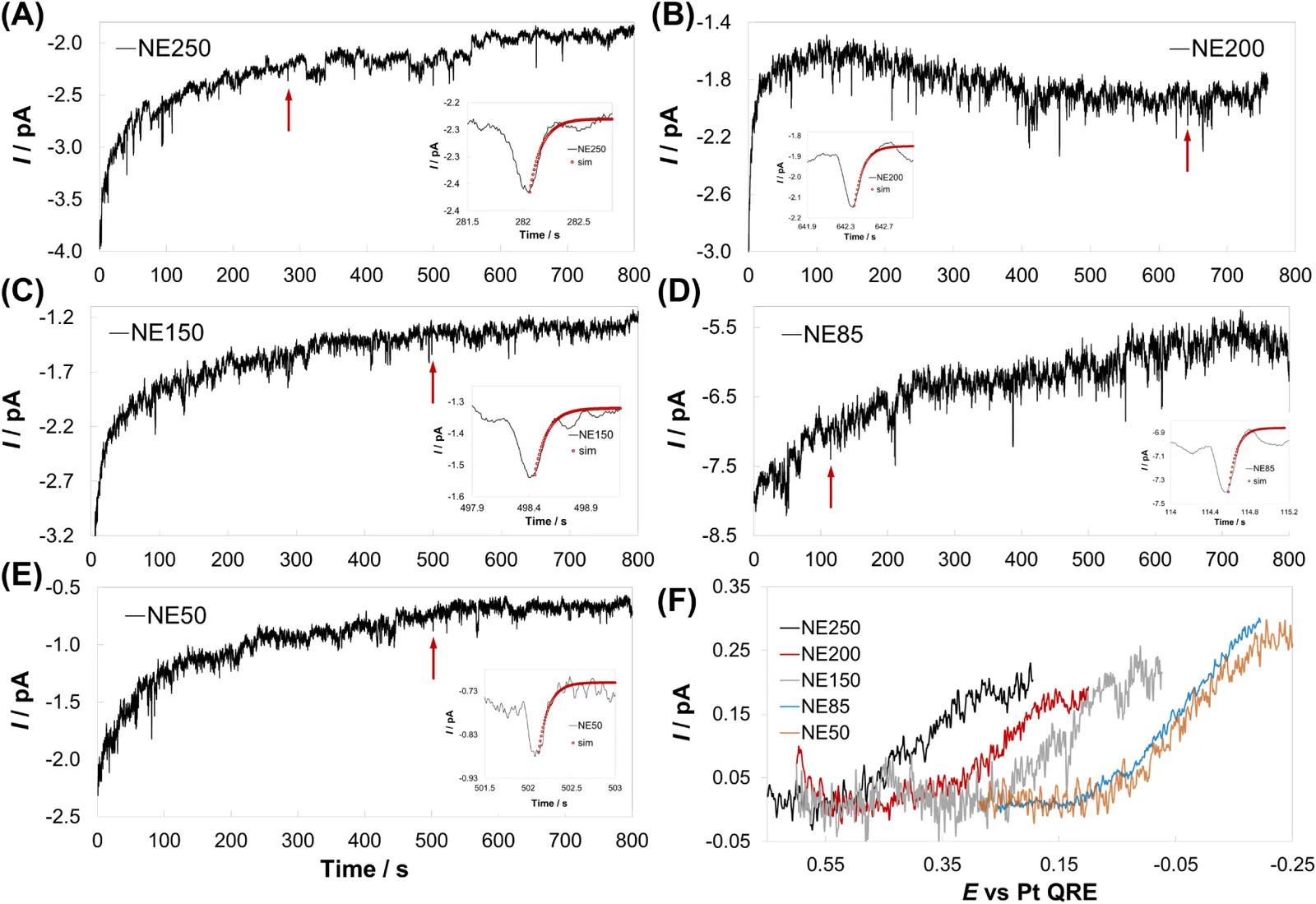
*i-t* curves during amperometric SEE with (A) NE250 under 0.85 V anodic potential, (B) NE200 under 0.90 V, (C) NE150 under 0.90 V, (D) NE85 under 0.95 V, and (E) NE50 under 0.95 V. (F) Continuous voltammograms from trapped NE250, NE200, NE150, NE85, and NE50 under a Pt UME coupled with amperometric SEE measurements are shown after background subtraction. All scan rates are 25 mV/s. Each solution contains 8 pM NEs in 10 mM NH_4_PF_6_. A Pt QRE was used as a reference electrode. For clarity, forward waves were shown in voltammograms.

The integrated charges for each current spike ranged from 10 to 50 fC, with approximately 25 fC having the highest probability (*N* = 15) (Figure S5). During this integration, we only counted current spikes with magnitudes at least three times greater than the inherent background noise level of ∼50 fA. Integrated charges of 20–30 fC as the highest frequency correspond to 200–300 zmoles of Fc oxidized inside individual NEs. In our previous study, we found that a single NE250 could carry up to ca. 300 zmoles of Fc molecules (equivalent to 30 fC).^17^ This high capacity is due to the high solubility of Fc via intermolecular interactions within NE components, including van der Waals interactions, hydrogen bonding, and noncovalent interactions such as lone pair-π* inside a NE.^18^ The consistency of the estimated charges (20–30 fC at each NE250, *N* = 15) during our present measurements indicates the full electrolysis of Fc inside the NE under the given condition (0.85 V vs Pt QRE). The corresponding charge distribution curves are shown in Figure S5A.

Highly similar amperometric SEE data and charge distribution curves were obtained for NE200, NE150, NE85, and NE50 when applying higher anodic potentials (0.9–0.95 V vs Pt QRE). At 0.85 V, however, these NEs exhibited only a few current spikes and lower integrated charges than 10 fC because the potential was insufficient for full electrolysis of Fc (Figures S6, S7). Applying higher potentials shifted the peak charge distribution to 20–30 fC (*N* = 15) (see Figure 4B–E and Figure S5). This indicates that full electrolysis of internal Fc requires a higher electric potential to overcome the interfacial tunneling barrier and facilitate ET. Consequently, these variations in required anodic potential directly reflect the structural differences among the NE interfaces.

To understand how interfacial structures affect electrochemical behavior of NEs, we performed continuous voltammetry immediately after each amperometric SEE. During this process, all Fc^+^ trapped inside the NEs under a Pt UME were electrochemically reduced across the NE interfaces. Figure 4F shows the resulting voltammograms for the trapped NEs (*N* = 10 for each NE). The signal profiles varied distinctly across the series; background-subtracted curves became sequentially sluggish and shifted negatively from NE250 down to NE50. Because voltammetry is less sensitive than amperometric SEE, we intentionally trapped multiple NEs for these measurements. Notably, NEs exhibited steady-state voltammograms, which occur when the scan rate is significantly slower than the diffusion rate,^31^

1 $$v\ll RTD/nFr^{2}$$

where *v* is the scan rate (25 mV/s), *R* is the gas constant (8.314 J/mol·K), *T* is the temperature (298 K), *D* is the diffusion coefficient (*D*_Fc_ = 10^–7^ cm^2^/s),^17^ *n* is the number of electrons transferred, *F* is the Faraday constant (96485 C/mol), and *r* is the radius of the electrode (i.e., the radius of the contact point between a Pt UME and a NE in this system, considered as an effective fitting parameter). Due to the extremely small radius of the contact point between a NE and a Pt UME,^17^ 25 mV/s is much slower than the diffusion rate for Fc^+^ reduction inside NEs (e.g., 2.56 × 10^9^ V/s with a contact point radius, *r* = ∼19 pm), thereby yielding steady-state voltammograms at trapped NEs. The effective contact point radius varied within 5.7–19 pm in the analysis. Notably, the ratio between these contact point radii and the diameter of NEs ranges from 0.014 to 0.047%, which is nearly similar (0.019–0.036%) to that of with nano- to microemulsions studied by Bard and coworkers.^15^ In addition, the anticipated steady-state current, ∼0.3 pA with a contact point radius of 5.7 pm, is well agreed with the experimental voltammograms in Figure 4F (more details in SI).

As hypothesized in Figure 2, the voltammograms in Figure 4F demonstrate that the presence and the effective thickness of a dynamic interfacial layer at NEs act as a tunneling barrier, significantly affecting the interfacial ET kinetics. These reactions are modeled as nonadiabatic ET, allowing for quantitative analysis to extract ET kinetic data and structural information in the following section.

### Dependence of the Electron Transfer Rate on the Effective Interfacial Layer Thickness of NE

The voltammograms in Figure 4F indicate that Fc^+^ reduction at the NEs is driven by ET via tunneling through the dynamic interface composed of TFAB^–^. In this system, the exponential dependence of the ET rate constant between an electrode and redox species on the tunneling distance (i.e., interfacial layer thickness, *d*) can be described as,^31^

2 $$k\left(d\right)=k_{b}\left(0\right)e^{-\beta d}$$

where *k*_*b*_(0) and *k*(*d*) are the heterogeneous ET rate constant values at the bare electrode surface (*d* = 0) and at the distance *d* from the electrode, respectively, and β is the tunneling decay coefficient (Å^–1^). To further quantitatively interpret ET rates under the nonadiabatic conditions, we adapt classical Marcus–Hush theory as,^24,32,33^

3 $$k=\nu_{n}\kappa_{el}\kappa_{n}$$

where *k* is the heterogeneous ET rate constant, ν_*n*_ is the effective nuclear vibration frequency, κ_*el*_ is the electronic transmission coefficient, and κ_*n*_ is the nuclear reorganization factor. Among these three quantities, κ_*el*_ contains the exponential decay with distance as,^24,31^

4 $$\kappa_{el}\left(d\right)=\kappa_{el}\left(0\right)e^{-\beta d}$$

where κ_*el*_(0) and κ_*el*_(*d*) are the κ_*el*_ values at the electrode surface (*d* = 0) and at a distance *d* from it, respectively. In the nonadiabatic regime at larger distances, the electronic coupling is getting weaker, thus κ_*el*_(*d*) < 1, which can be reduced to:^24,33,34^

5 $$\kappa_{el}\left(d\right)=\frac{\nu_{el}^{0}}{\nu_{n}}e^{-\beta d}$$

where $\nu_{\mathrm{el}}^{0}$ is energy-independent electron-hopping frequency at *d* = 0.

According to the Landau–Zener formalism, $\nu_{\mathrm{el}}^{0}$ can be further expressed as,^24,35^

6 $$\nu_{el}^{0}=\frac{4\pi^{2}\rho_{M}{\left(H_{12}^{0}\right)}^{2}}{h\sqrt{4\pi \frac{\lambda }{k_{B}T}}}$$

where λ is the reorganization energy of the redox mediator (0.975 eV for ferrocene),^36^ *h* is the Planck constant (4.16 × 10^–15^ eV s), *k*_B_ is Boltzmann’s constant (8.62 × 10^–5^ eV/K), ρ_*M*_ is the density of states of the metal (2.3 eV^–1^ atom^–1^ for Pt),^37^ $\left(H_{12}^{0}\right)$ is the electronic coupling element at *d* = 0 (≈0.1 eV),^24^ and *T* is the temperature.

Particularly, the heterogeneous formal rate constant *k* ^0′^ (cm/s) at the formal potential *E* = *E* ^0′^ can be estimated by integrating *k* over various distances from the electrode,^24^

7 $$k^{0^{\prime }}=\int_{d=0}^{\infty }k\left(E=E^{0^{\prime }}\right)dx=\nu_{n}\kappa_{n}\left(E=E^{0^{\prime }}\right)\int_{d=0}^{\infty }\kappa_{el}dx$$

where κ_*n*_ at *E* = *E* ^0′^ is expressed with the reorganization energy of λ by Feldberg and Sutin as,^24^

8 $$\kappa_{n,E=E^{0^{\prime }}}=\frac{\pi }{\sqrt{1+\frac{\pi k_{B}T}{\lambda }}}\exp\left[-\frac{\lambda }{4k_{B}T}\right]$$

Finally, *k* ^0′^ derived by Feldberg and Sutin for nonadiabatic reactions at the bare electrode surface (*d* = 0), i.e., $k_{b}^{0^{\prime }}$ is given as,^24^

9 $$k_{b}^{0^{\prime }}=\frac{2\pi^{2}\rho_{M}{\left(H_{12}^{0}\right)}^{2}}{\beta h\sqrt{1+\frac{\lambda }{\pi k_{B}T}}}e^{-\lambda /4k_{B}T}$$

Using 1 Å^–1^ for β value reported for a bare Pt electrode,^38^ $k_{b}^{0^{\prime }}$ can be estimated as 22.7 cm/s for Fc/Fc^+^ at a bare Pt UME. For other outer-sphere redox mediators, a similar range of *k* ^0′^ has been experimentally determined.^39–41^

From eqs 2 and 9, the distance-dependent formal rate constant *k* ^0′^(*d*) can be finally yielded as:

10 $$k^{0^{\prime }}\left(d\right)=k_{b}^{0^{\prime }}e^{-\beta d}$$

Subsequently, we simulated voltammograms using the Butler–Volmer formalism with adapting the distance-dependent formal rate constant *k* ^0′^(*d*) from eq 10,^31^

11 $$i=FAk^{0^{\prime }}\left(d\right)\left[C_{O}\left(0,t\right)e^{-\alpha F/RT(E-E^{0^{\prime }})}-C_{R}\left(0,t\right)e^{\left(1-\alpha \right)F/RT(E-E^{0^{\prime }})}\right]$$

where *i* is the current, α is the transfer coefficient, *F* is the Faraday constant, *R* is the gas constant, *E* ^0′^ is the formal potential, and *C*_*O*_(0,*t*) and *C*_*R*_(0,*t*) are the concentrations of oxidized and reduced redox species at the electrode surface at a given time, *t*, respectively.

Herein, β for the dynamic interface of TFAB^–^ at NEs in eq 10 is theoretically estimated as:^31,42^

12 $$\beta \approx \frac{4\pi \sqrt{2m\Phi_{Barrier}}}{h}\approx \frac{4\pi \sqrt{2m\left(\Phi_{metal}-\chi \right)}}{h}\approx 1.02{Å}^{-1}eV^{-1/2}\times {(\Phi_{metal}-\chi )}^{1/2}$$

where *m* is the mass of an electron (9.1 × 10^–28^ g), *h* is the Planck constant, and Φ_*Barrier*_ is the potential barrier height at a contact between a Pt metal and a layer of TFAB^–^, which is approximated as the difference between the metal work function Φ_*metal*_ (for Pt, 5.93 eV),^43–45^ and the electron affinity χ of the dielectric (for TFAB^–^, 5.85 eV).^46^ Resultantly, β for TFAB^–^ interfacial layer could be estimated as 0.29 Å^–1^, which is within a reasonable range compared to β = 0.2–0.3 Å^–1^ reported for aromatic chains.^47–49^

Using the finite element analysis with COMSOL Multiphysics and the theoretically estimated values of $k_{b}^{0^{\prime }}$ and β (i.e., 22.7 cm/s and 0.3 Å^–1^, respectively), a series of voltammograms were simulated by varying the interfacial layer thickness *d* from 0 to 35 Å (currents are normalized with the steady-state currents in Figure 5A). The voltammograms are gradually shifted to more negative potentials, spanning c.a. 500 mV difference with increasing interfacial layer thickness up to 35 Å. Moreover, the voltammograms significantly change at *d* as small as 0.1 to 2.5 Å, or 2.5 to 5 Å. These simulated voltammograms were compared with the experimental curves (shown in Figure 4F) to determine *d* at various NEs. In Figure 5B, experimental voltammograms with NE250, NE200, NE150, NE85, and NE50 (*N* = 10 for each NE) agreed well with theoretical curves with the interfacial layer thickness as 0.1, 5.0, 17.0, 25.0, and 30.0 Å, respectively. From eq 10 with β = 0.3 Å^–1^ and the *d* values determined by voltammetric fitting, corresponding *k* ^0′^ at each NE could be estimated as 22.0, 5.14, 0.145, 0.0135, and 0.00306 cm/s, separately in a given order. Noticeably, the retardation of ET by the dynamic TFAB^–^ interface at NEs is pronounced, since *k* ^0′^ values decrease nearly 4 orders of magnitude within ∼30 Å thick interfacial layers, thereby leading to prominently distinguishable voltammograms across different NEs. As these kinetic parameters are measured and determined with multiple NEs, they could represent collective responses. Also, the variation of contact radius between 5.7 and 19 pm exhibits an ∼10 mV shift in the simulated voltammograms, thereby leading to the uncertainty at the 1–2 Å level in the estimation of interfacial layer thickness at NEs (more details in SI and Figure S12).

**Figure 5:**
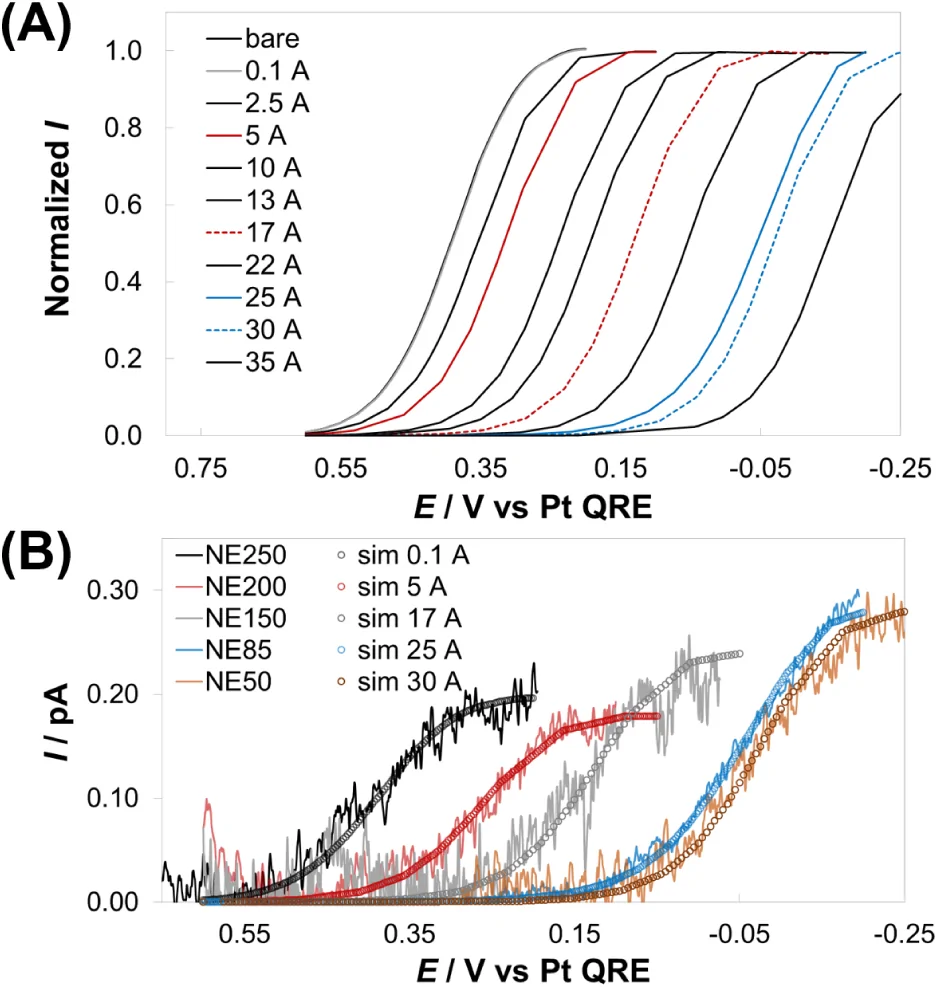
(A) Simulated voltammograms for the Fc^+^ reduction of a hypothetical system with parameters given in SI and the interfacial layer thickness *d* varying between 0 and 35 Å. The current is normalized by the maximum steady-state current. Simulations were carried out for the Fc^+^ concentration inside NEs as a bulk, an electrode diameter from a contact point between a NE and a Pt UME, and a diffusion coefficient of Fc^+^ inside a NE, *D*_Fc_ = 10^–7^ cm^2^/s.^17^ (B) Voltammetric fitting with experimental voltammograms from Figure 4F. Current magnitudes in theoretical simulation were adjusted by varying a contact point radius (ranging within 5.7–19 pm) between a NE and a Pt UME as an effective fitting parameter instead of the number of trapped NEs. For clarity in voltammetric fitting, only forward waves were used.

Further, the change in ln *k* ^0′^ values as a function of *d* is plotted in Figure 6, where the curve exhibits a good linearity with a slope of 0.296 and a y-intercept of 3.137 corresponding to β and ln $k_{b}^{0^{\prime }}$ , respectively. These values are highly consistent with theoretically determined $k_{b}^{0^{\prime }}$ = 22.7 cm/s and β = 0.29 Å^–1^ using eqs 9 and 12. A uniform β over the interfacial layer thickness range of 0.1 Å to 30 Å strongly indicates that the resulting interfacial ET is dominated by a single step-tunneling mechanism without transitioning to hopping.^38,50,51^ Typically, a transition to hopping in charge transports (through tethered molecular wires, organic, or inorganic films) is evidenced by a breakpoint in β or a much shallower distance dependence where β < 0.1 Å^–1^.^38,51^ Such transitions typically occur at approximately 1 nm in nonconjugated systems^50^ and 4 nm in conjugated ones.^52–54^ For TFAB^–^ at the NE interface, the electron-cloud density on the boron is delocalized by the strong-withdrawing effect of the fluorine on the four perfluorophenyl rings.^55^ This could increase the effective electron affinity of TFAB^–^,^46^ lowering the effective barrier height in eq 12 and minimizing interference in charge transport, thereby facilitating a tunneling-controlled ET over the entire 30 Å range in this NE system.

**Figure 6:**
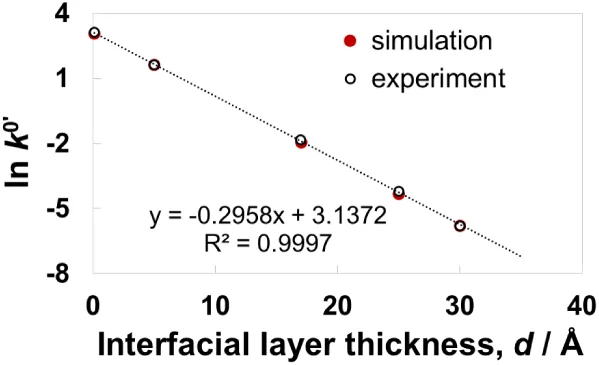
Dependence of the electron transfer rate k^0′^ on the interfacial layer thickness of a NE, *d*. Natural logarithm of the *k* ^0′^ from each NE is plotted as a function of the tunneling distance, i.e., the dynamic interfacial layer thickness of a NE for the Fc^+^ reduction reaction.

In fact, the complexity of dynamic interfaces at NEs stems from potential steric hindrance by F-127 surfactants, alongside uncertainties regarding the compactness, alignment, and stacking density of TFAB^–^ layers near the interface. For NE250, which features a 0.1 Å thick dynamic interface, the simulated voltammogram nearly overlaps with that of a bare electrode (Figure 5A, gray and black solid lines on the leftmost side), respectively, suggesting the absence of a borate interface. Also, this rapid ET at NE250—resembling a bare electrode—indicates that F-127 surfactants have a negligible effect on interfacial ET, likely due to the high structural flexibility of the F-127 block copolymer.^56–59^ Given the inherent uncertainties within the TFAB^–^ interface, the thickness (*d*) determined for each NE via voltammetric fitting represents the effective thickness of the tunneling-active interfacial layer. Notably, because spontaneous emulsification imparts high kinetic stability to the NE structure, the interface can be considered inherently pinhole-free.^21,22^ This allows us to exclude confounding effects from contact resistance or contact properties on electron tunneling or hopping, which are common complications in molecular wire or ultrathin-film devices.^60–66^

### Accordant Evidences for Tunneling-Dominant Electron Transfer

Validating the tunneling-dominated ET mechanism in NEs is crucial for accurately determining the effective thickness of their dynamic interfacial layers. To rigorously confirm this mechanism over multistep hopping, we scrutinized the transfer coefficient, α. As depicted in Figure 7A, α values for NE50, NE85, NE150, NE200, and NE250 were extracted from voltammetric fitting and range between 0.43 and 0.50. Because these values remain independent of the interfacial layer thickness (*d* = 0.1–30 Å), they strongly corroborate a concerted, single-step tunneling mechanism. This aligns well with theoretical expectations, where a nearly uniform α = 0.50 ± 0.05 is the established benchmark for tunneling-dominated ET in electrochemical systems.^23,67^

**Figure 7:**
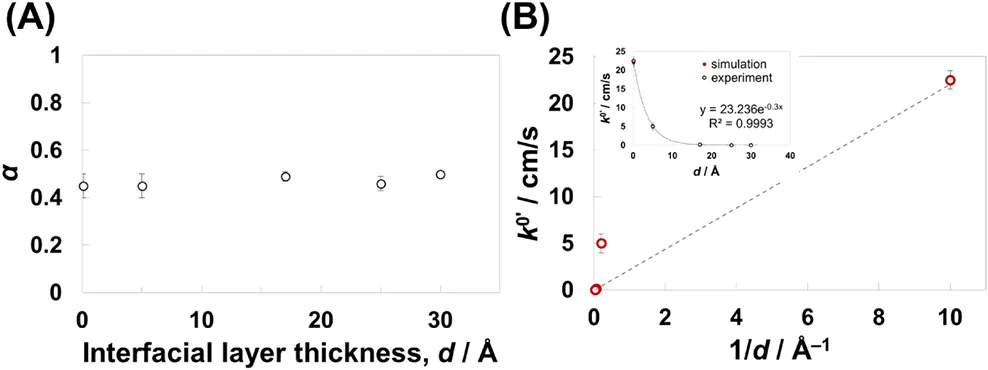
(A) Dependence of the transfer coefficient, α (open circles, determined by simulation), as a function of the effective interfacial layer thickness of a NE, *d.* (B) Electron transfer rate, *k* ^0′^ vs 1/*d*. Inset, an equivalent plot of Figure 6 exhibits a clear exponential dependence of electron transfer rate k^0′^ on an effective interfacial layer thickness of NEs.

In contrast, multistep-hopping-prevailing ET—typically observed in molecular wires or ultrathin film devices—exhibits a distinct dependence on the tunneling distance.^38,50,68,69^ In these systems, α (or 1 – α) drops from 0.5 to extraordinarily low values of 0.1–0.2 as the tunneling distance increases. Hopping mechanisms often yield nonideal α values, particularly in films or redox polymers, where asymmetrical activation energy barriers, structural variations, and imperfectly symmetrical redox sites complicate the charge transfer.^38^ Additionally, the overall α in a hopping process is dictated by the kinetics of the rate-determining step within the sequence, introducing further interpretive complexity.^31^ Similar anomalous α values have also been reported for disordered materials deposited on metal electrodes.^69^ Mallouk and coworkers attributed the apparently low transfer coefficients of ∼0.2 to the highly defective structures of multilayer-metal phosphate films on gold electrodes during electron hopping.^69^ In contrast, intrinsically pinhole-free NE interfaces provide a unique ET system that effectively prevents measurement artifacts caused by defects like pinholes,^21,22^ thereby ensuring a highly reliable interpretation of the obtained electrochemical data.

The contrast between single-step tunneling and multistep hopping is especially clear in the distance dependence of ET rates, *k* ^0′^. While hopping processes generally display a shallower distance dependence—often inversely proportional to distance or its square —tunneling is characterized by an exponential decay, i.e., e^–β*d*^.^31,51,67,70,71^ To elucidate this, experimentally determined *k* ^0′^ values were plotted against both 1/*d* and *d* in Figure 7B and its inset, alongside theoretically determined values. The distance dependence in our NE system is evidently nonlinear with 1/*d* (Figure 7B). Instead, a plot of *k* ^0′^ versus *d* manifests exponential behavior (Figure 7B inset). This explicitly exhibits typical tunneling across the entire NE interfacial thickness range of 0.1 to 30 Å, where the pre-exponential factor of 23.2 cm/s closely matches $k_{b}^{0^{\prime }}$ = 22.7 cm/s from eq 9, and the decay rate of −0.3 is consistent with β = ∼0.3 Å^–1^ from eq 12. Overall, the magnitude of the distance decay constant β, the distance independence of α (which remains close to 0.5 along layer thickness *d*), and the clear exponential dependence of *k* ^0′^ on *d* confirm that single-step tunneling dominates interfacial charge transport in this NE system.

## Conclusions

In this work, we developed multimodal single-entity electrochemistry to enable the *in situ* study of electron-tunneling through dynamic interfaces at authentic NEs. Amperometric SEE-coupled voltammetry was employed to monitor the real-time trapping of individual NEs onto a Pt UME and to *in situ* investigate electron tunneling in interfacial ET reactions during potential-scanning. This approach yields comprehensive insights into ET kinetics and interfacial structures at NEs. Voltammetric data were quantitatively analyzed with adapting Marcus–Hush theory to determine key parameters, including effective interfacial thickness, tunneling-distance decay constants, heterogeneous ET rate constants, and transfer coefficients across a series of NEs. Notably, rigorous comparisons of electron tunneling versus hopping were utilized to probe ET at discrete NEs. All acquired data confirm that single-step tunneling dominates the overall interfacial ET reactions within a 0.1–30 Å range of the dynamic interface. The power of this novel electroanalytical capability was demonstrated by discriminating dissimilar dynamic interfacial structures of various NEs at angstrom resolution, surpassing morphological size differences that conventional analytical techniques could not elucidate. Furthermore, the high kinetic stability of NE integrity—achieved via spontaneous emulsification—provides an inherently pinhole-free system. This makes the platform uniquely suited for exploring interfacial ET *in situ*, eliminating concerns regarding defect-induced artifacts or contact resistance effects. This innovative electroanalytical approach will be broadly useful for elucidating the structure–function relationships of diverse lipid nanocarriers, facilitating the deliberate design and optimization of these carriers to achieve targeted functionalities, such as enhanced delivery efficiency, controlled release kinetics, and superior structural stability.

## Supplementary Material





## Notes

Supplementary experimental section; the interfacial coverage of F-127 on each NE; characterization of nanoemulsions by dynamic light scattering (DLS); electrochemical characterization of Pt UMEs; cyclic voltammetry for Fc oxidation in THF cocktail solution; charge distribution curves from amperometric SEE data with NE250, NE200, NE150, NE85, and NE50 under full electrolysis condition; amperometric SEE data with NE200, NE150, NE85, and NE50 at anodic potential, 0.85 V; charge distribution curves from amperometric SEE data with NE200, NE150, NE85, and NE50 measured at 0.85 V; fitting *i-t* decay with bulk electrolysis model; amperometric SEE data with sampling time 1.6 ms; COMSOL multiphysics simulation model; dependence of simulated voltammograms on α values; estimation of a steady-state current in MSEE; the effect of contact radius, an effective fitting parameter, on the estimation of interfacial layer thickness (PDF)
Global definitions; Model 1; Study 1; and Results (PDF)
