# Supplementary material for: In Situ Study of Electron-Tunneling through Dynamic Interface at Individual Nanoemulsions by Multimodal Single-Entity Electrochemistry
Source: J Phys Chem B. 2026 Oct 21;130(44):11931–41. doi: 10.1021/acs.jpcb.6c04932 (PMC13746782; doi:10.1021/acs.jpcb.6c04932)
Supplement: Supplementary file 1 [file jp6c04932_si_001.pdf]

Supporting information I

In Situ Study of Electron-Tunneling through Dynamic Interface at Individual Nanoemulsions by Multimodal Single-Entity Electrochemistry

Anuradha Khatri Chhetri,[a] Mandip Adhikari,[a] and Jiyeon Kim[a,*]

[a] Department of Chemistry, University of Rhode Island, Kingston, Rhode Island, 02881, United States

* To whom all correspondence should be addressed. E-mail: jkim25@uri.edu.

Table of Contents

1. Supplementary Experimental Section.--S2
2. The interfacial Coverage of F-127 on each NE.--S3
3. Characterization of Nanoemulsions by Dynamic Light Scattering (DLS).---------------------------S4
4. Electrochemical Characterization of Pt UMEs.--S6
5. Cyclic Voltammetry for Fc oxidation in THF Cocktail Solution. -------------------------------------S7
6. Charge Distribution Curves from Integrated Current Spikes in Amperometric SEE Data with NE250, NE200, NE150, NE85, and NE50 under Full Electrolysis Condition.----------------------S8
7. Amperometric SEE data with NE200, NE150, NE85, and NE50 at Anodic Potential, 0.85 V.---S9
8. Charge Distribution Curves from Integrated Current Spikes in Amperometric SEE Data with NE200, NE150, NE85, and NE50 Measured at 0.85 V. ---S10
9. Fitting *i*-*t* Decay with Bulk Electrolysis Model.---S11
10. Amperometric SEE data with Sampling Time 1.6 ms. ---S13
11. COMSOL Multiphysics Simulation Model.---S15
12. Dependence of Simulated Voltammograms on α Values. --S18
13. Estimation of a Steady-State Current in MSEE.--S20
14. The Effect of Contact Radius, an Effective Fitting Parameter on the Estimation of Interfacial Layer Thickness.---S21

1. Supplementary Experimental Section.

Pt UME preparation undergoes three steps of stretching, sealing and pulling. Each parameter of laser puller and the resulting dimensions are given in the Table S1.

Table S1. Pulling programs and parameters of the laser puller for Pt UME preparation: stretching, sealing and pulling.

Step	Parameter	Operation
Stretching	*H* = 310, *F* = 4, *V* = 14, *D* = 120, *P* = 0<br>*H* = 310, *F* = 4, *V* = 14, *D* = 120, *P* = 0<br>*H* = 310, *F* = 4, *V* = 14, *D* = 120, *P* = 0<br>*H* = 310, *F* = 4, *V* = 15, *D* = 120, *P* = 0<br>*H* = 310, *F* = 4, *V* = 15, *D* = 120, *P* = 0<br>*H* = 310, *F* = 4, *V* = 15, *D* = 120, *P* = 0<br>*H* = - , *F* = 4, *V* = 15, *D* = 120, *P* = 0	Right after the sixth line, program is stopped manually.<br>Resulting dimension: OD 145-150 μm, ID 45-50 μm.
Sealing (with stoppers)	*H* = 340, *F* = 4, *V* = 14, *D* = 120, *P* = 0	Program should be run for 13.57 – 13.65 s.<br>Resulting dimension: OD 145-150 μm, ID 25 μm.
Pulling	*H* = 340, *F* = 3, *V* = 15, *D* = 118, *P* = 0	Electrode is pulled in one loop.

H = Heat, F = Filament, V = Velocity, D = Delay, and P = Pull.

2. The interfacial Coverage of F-127 on each NE.

We estimated the interfacial coverage of F-127 on each NE by the ratio of total number of F-127 molecules over the total NE surface area in Table S2. Since no discernible peak at ~10 nm for F-127 micelle was observed in DLS analysis,[S1] we did not consider the contribution of F-127 micelle to this coverage estimation. The coverage ranges from 9.4 molecules/nm^2, 7.6 molecules/nm^2, 5.9 molecules/nm^2, 3.5 molecules/nm^2, and 2.5 molecules/nm^2 for NE250, NE200, NE150, NE85, and NE50, respectively. This level of interfacial coverage reflects full monolayers with maximum adsorption, similar to 3.2 molecules/nm^2 of F-127 on iron oxide nanoparticles,[S2] or 5.2 ~11.2 molecules/nm^2 of F108 chains (similar structure to F127) on polystyrene nanoparticles with diameter of 69-482 nm.[S3] In NE synthesis, we induced the spontaneous emulsification by evaporating THF solvent with dry N_2 gas, which forms highly monodisperse NEs. Resultantly, the size and the electrochemical behavior related to the intrinsic interfacial structures of NEs are highly uniform confirmed by TEM and SEE measurements.[S4] Accordingly, the high uniformity in the amount and distribution of F-127 at the NE interface was assumed across individual NEs as well.

Table S2. Interfacial Coverage of F127 (molecules/nm^2) on each NE.

	r_{NE} by DLS (nm)	F-127 concentration in NE final stock solution (mM)	F-127 coverage (molecules/nm^2)
NE250	21	0.18	9.4
NE200	21	0.15	7.6
NE150	22	0.11	5.9
NE85	23	0.064	3.5
NE50	28	0.038	2.5

3. Characterization of Nanoemulsions by Dynamic Light Scattering (DLS).

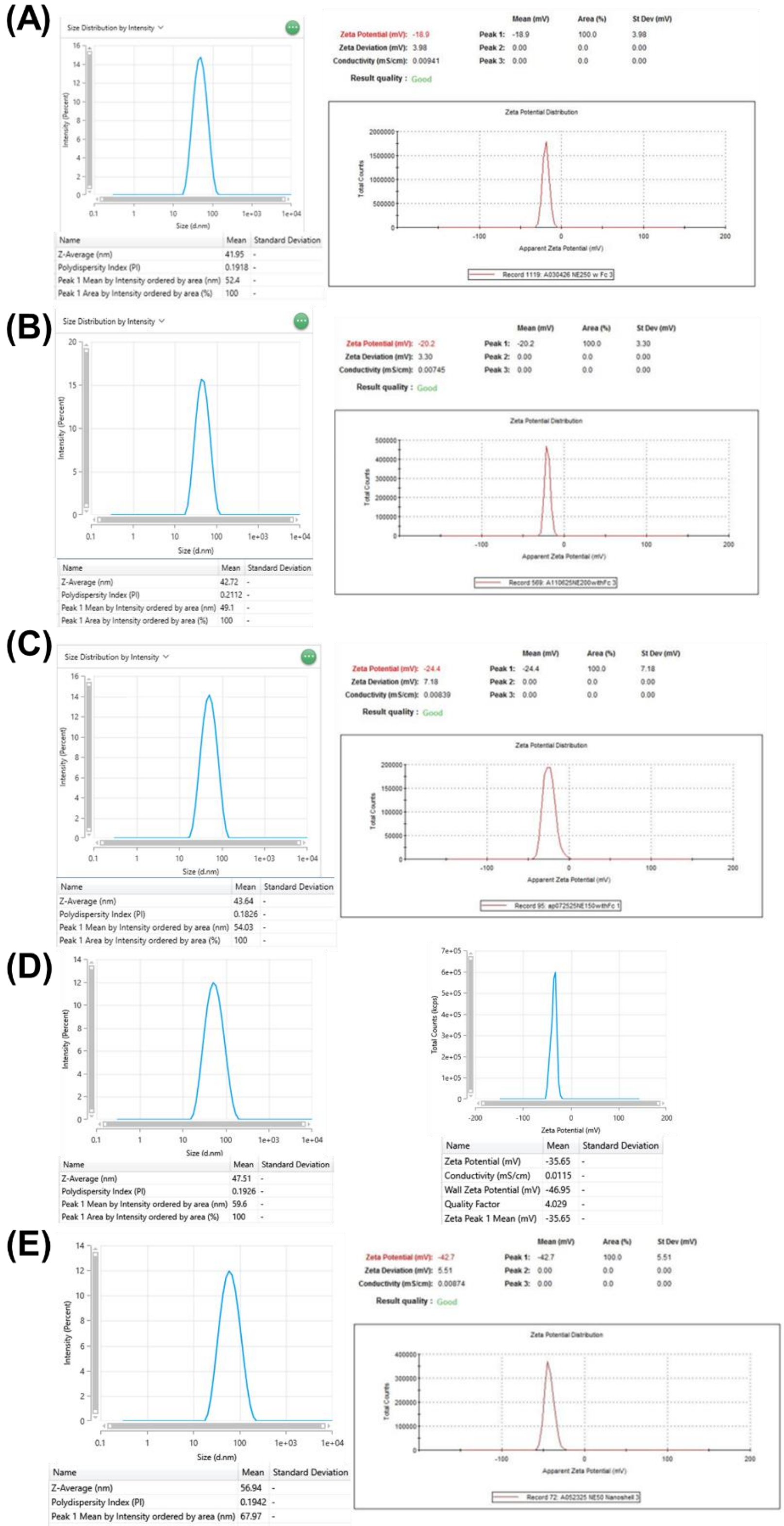

Figure S1. Raw data of DLS results showing average diameters, polydispersity index (PI or PDI), and average zeta potentials of (A) NE250, (B) NE200, (C) NE150, (D) NE85, and (E) NE50 diluted in nanopure water.

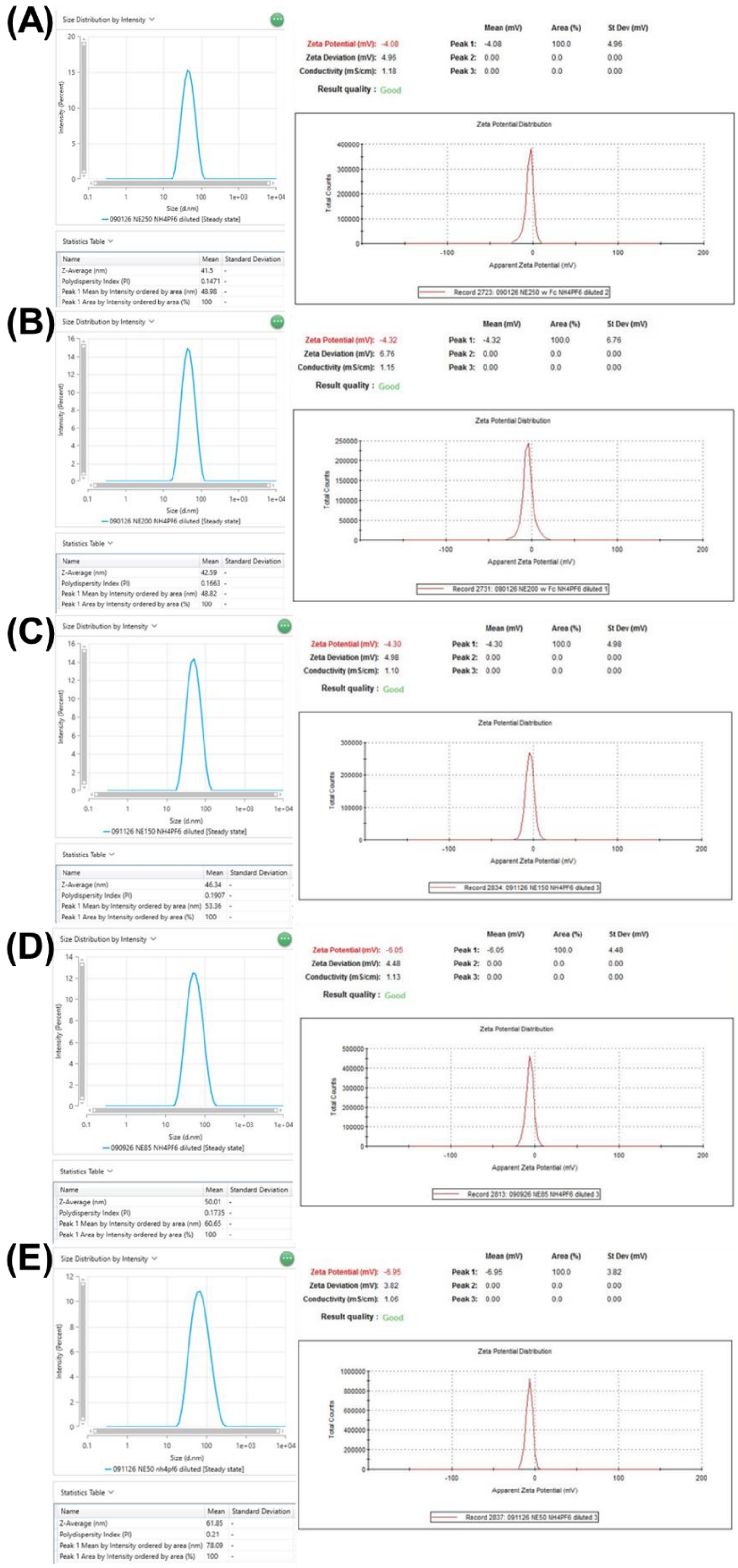

Figure S2. Raw data of DLS results showing average diameters, polydispersity index (PI or PDI), and average zeta potentials of (A) NE250, (B) NE200, (C) NE150, (D) NE85, and (E) NE50 diluted in 10 mM NH_4PH_6.

We showed the DLS data of sizes and ζ-potentials of NEs diluted in nanopure water in Figure S1 and diluted in 10 mM NH_4PF_6 electrolytes in Figure S2, respectively. Trend in NE size changes in 10 mM NH_4PF_6 are nearly identical to those in nanopure water, while only NE50 in NH_4PF_6 was about 10 % larger than the average diameter in water. However, ζ-potentials of all NEs in 10 mM NH_4PH_6 were uniform in –4 ~ –7 mV ranges regardless of their anticipated difference in interfacial structures. No clear trends in ζ-potentials were observed across the different NEs, limiting our insight into their nanostructural differences. In fact, this is a trivial result, where the moderate adsorption of a cationic solute at the Stern plane of a NE as well as the moderately high supporting electrolyte concentration yielding a thin diffuse layer could result in vastly different ζ-potentials from the intrinsic ζ-potentials of NEs. Contrarily, in nanopure water, little Stern layer adsorption with negligible effect of electrolyte considerably extending the diffuse layer could enable us to observe the ζ-potentials close to the true and intrinsic values.[S5]

4. Electrochemical Characterization of Pt UMEs.

A prepared Pt UME was electrochemically characterized by performing voltammetry in 1 mM FcMeOH, 10 mM NaCl solution (Figure S3). According to equation S1 for the limiting current at a Pt UME with *n* (transferred electron number, *n* = 1 for Fc), the radius of Pt UME is estimated as 0.5 µm.[S6]

$$i_{lim} = 4xnFD_iC_ia \qquad \text{(S1)}$$

where *F* is the Faraday constant (96485 C/mol), and *n*, D_i, and C_i are the transferred number of electrons, a diffusion coefficient, and a bulk concentration of redox mediator (i) in the aqueous electrolyte solution, respectively, and *x* is a function of glass outer radius (r_g) / Pt radius (*a*) of a Pt UME. r_g/a = 4 determined by optical microscope has *x* = 1.08.

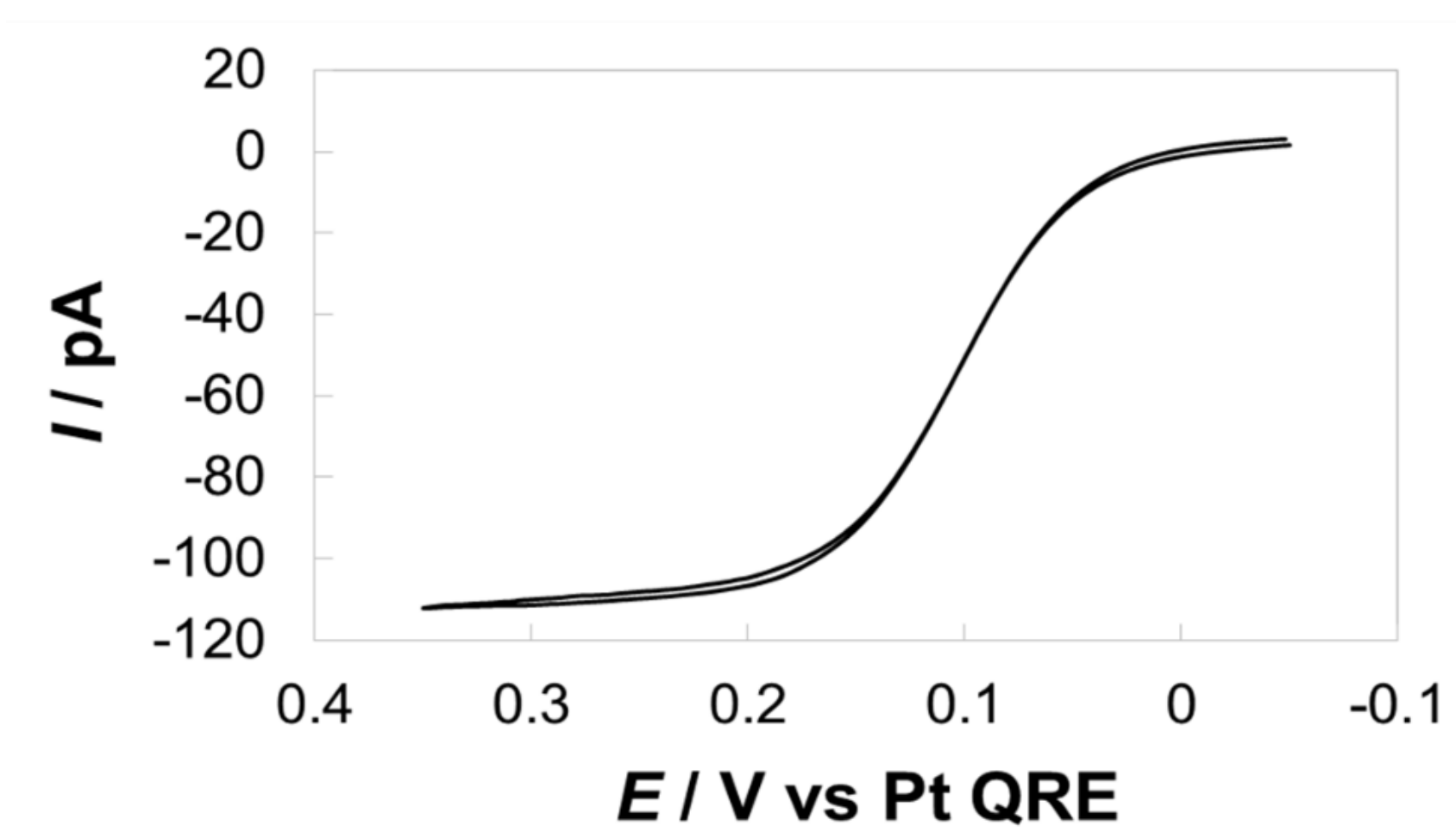

Figure S3. Voltammogram of FcMeOH oxidation using 0.5 µm radius Pt UME in 1 mM FcMeOH, 0.1 M NaCl solution. Scan rate was 25 mV/s. Pt QRE is used as a reference

5. Cyclic Voltammetry for Fc oxidation in THF Cocktail Solution.

To determine the diffusion-limiting potential for Fc oxidation inside a NE during MSEE measurements, we performed the voltammetry with THF cocktail solution containing Fc (Figure S4). Due to a low dielectric constant, 7.58 of THF, a large uncompensated resistance has been observed even with borate compounds as supporting electrolytes. Under this condition, we could obtain a diffusion limiting current at ~0.85 V vs Pt QRE. Since DOS used for a core of NEs has even lower dielectric constant, ε of 4.7 than THF, the presence of borate compounds ($TFAB^-$) in NE is necessary as a supporting electrolyte to reduce the uncompensated resistance and drive electrochemical reaction. The Pt UME tested here was also electrochemically characterized by voltammetry in 1 mM FcMeOH, 10 mM NaCl solution (see Figure S3).

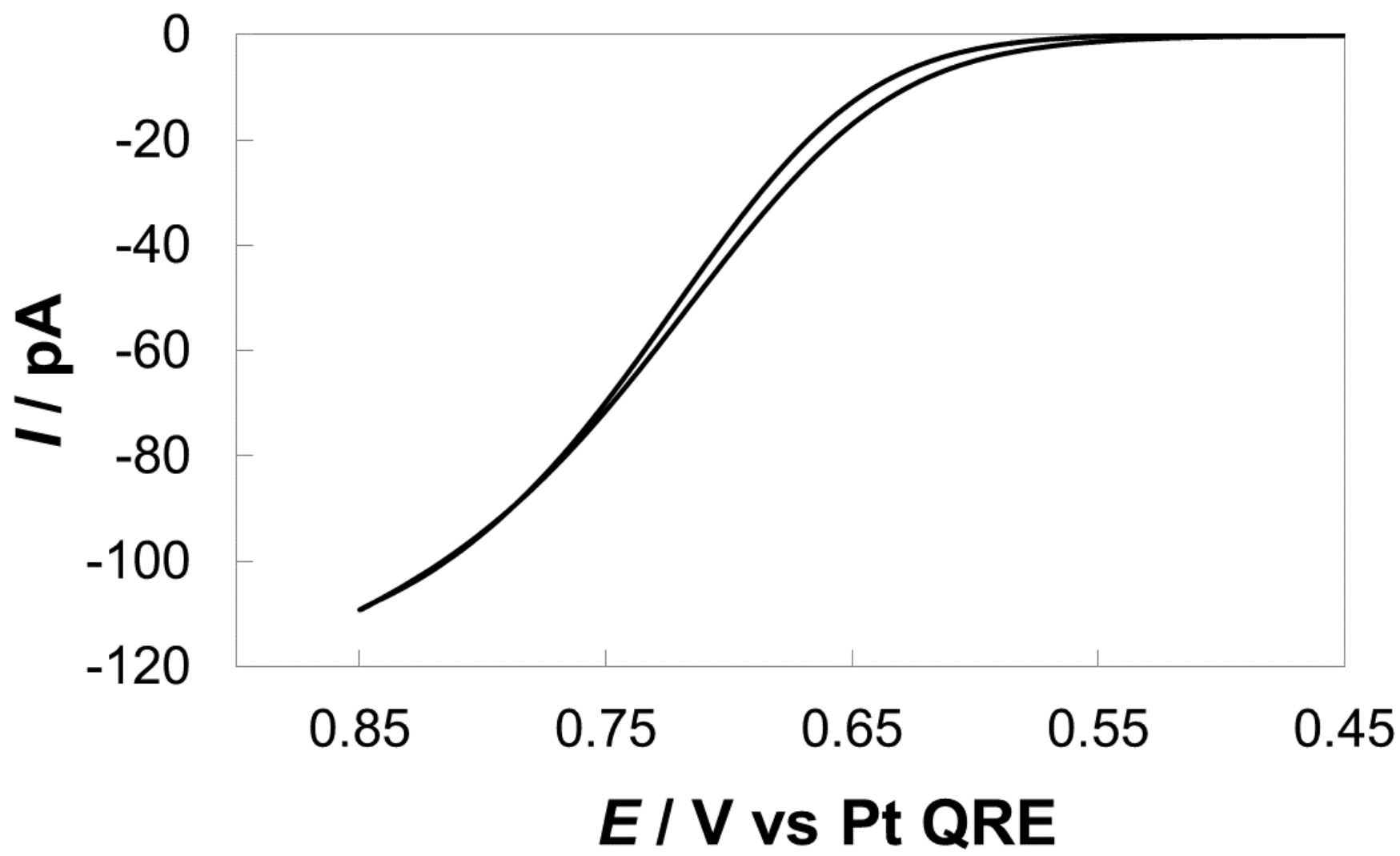

FigureS4. Voltammogram of Fc oxidation in the THF cocktail containing 1 mM Fc. Scan rate was 25 mV/s. Pt QRE is used as a reference electrode.

6. Charge Distribution Curves from Integrated Current Spikes in Amperometric SEE Data with NE250, NE200, NE150, NE85, and NE50 under Full Electrolysis Condition.

Each amperometric SEE data with NE250, NE200, NE150, NE85, and NE50 measured at 0.85 ~0.95 V was further analyzed to construct the charge distribution curves (N = 15). Integrated current spikes in amperometric SEE with NE250, NE200, NE150, NE85, and NE50 exhibit 20–30 fC as the highest frequency in the charge distributions over collision events (see Figure S5). This corresponds to 200–300 zmoles of Fc inside individual NEs undergoing full electrolysis, which are consistent with the original amount of Fc contained in each NE.[S4]

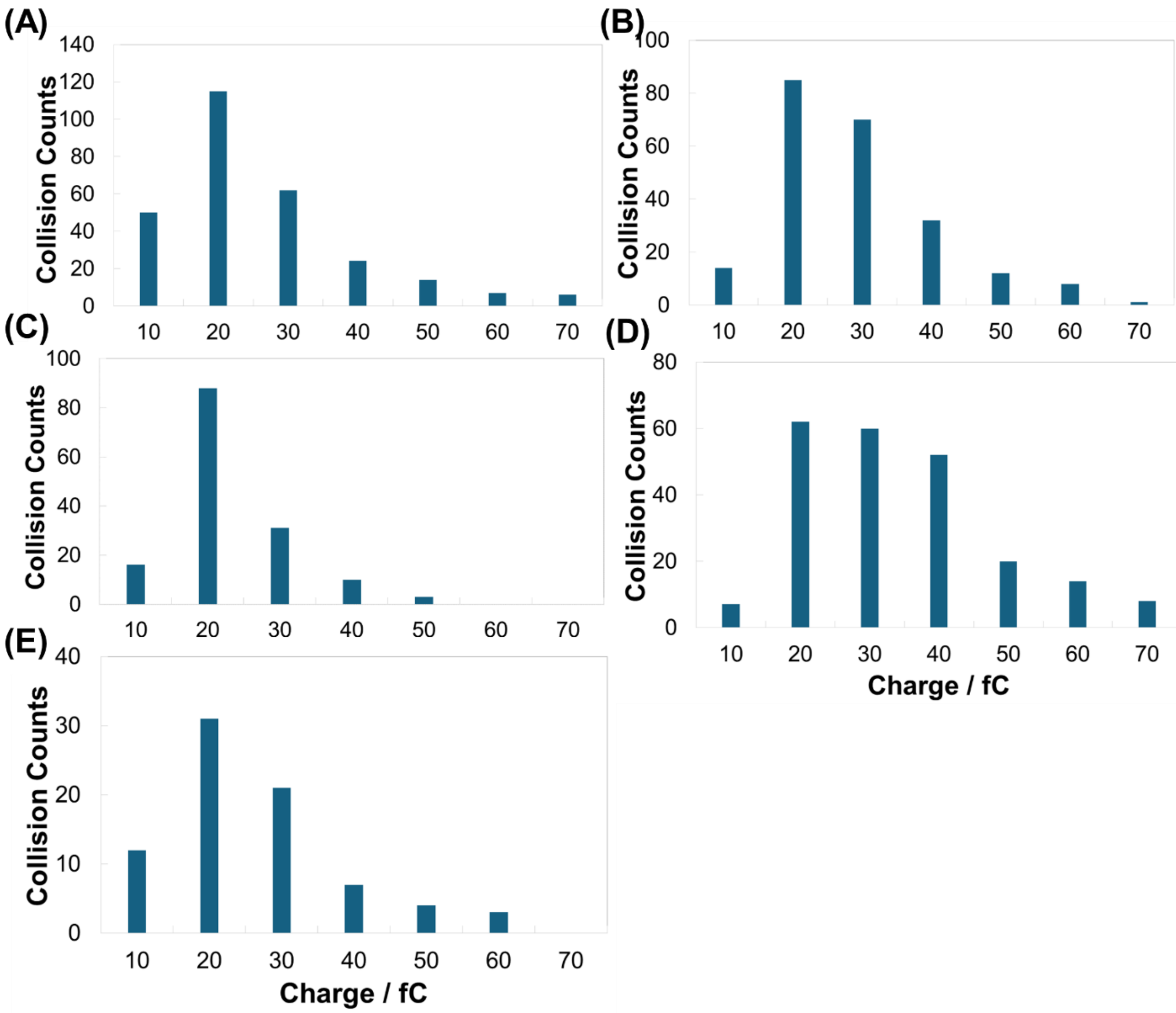

Figure S5. Charge distribution curves from the integrated current spikes in amperometric SEE data measured under full electrolysis condition (A) NE250, (B) NE200, (C) NE150, (D) NE85, (E) NE50 (Corresponding amperometric SEE data are shown in Figure 4).

7. Amperometric SEE data with NE200, NE150, NE85, and NE50 at Anodic Potential, 0.85 V.

Amperometric SEE were measured with NE200, NE150, NE85, and NE50 at anodic potential 0.85 V vs Pt QRE (N = 4), which was insufficient to overcome the tunneling barrier of interfacial layers and completely electrolyze all Fc inside NEs upon a collision between a NE and a Pt UME. Resultantly, no or barely discernible current spikes were obtained in Figure S6 (blue solid lines), whereas distinctly sharp current spikes were observed at the higher anodic potentials of 0.9-0.95 V vs. Pt QRE (black solid lines).

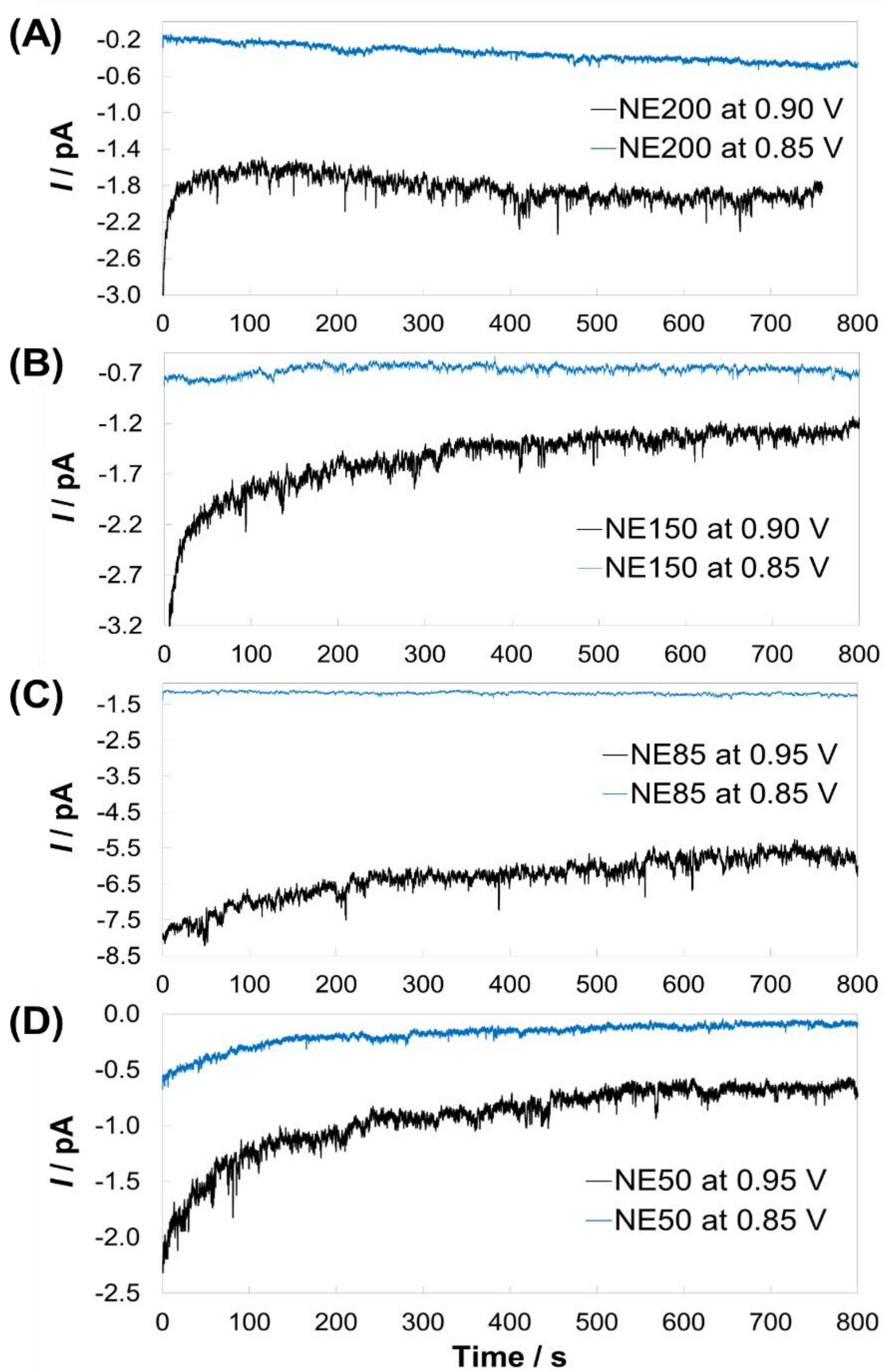

Figure S6. *i-t* curves during amperometric SEE (blue solid lines) with (A) NE 200, (B) NE150, (C) NE85, and (D) NE50 under anodic potential at 0.85 V vs Pt QRE. Each curves were compared with *i-t* curves measured at higher anodic potentials, 0.9-0.95 V (black solid lines) with full electrolysis of Fc during amperometric SEE measurements.

8. Charge Distribution Curves from Integrated Current Spikes in Amperometric SEE Data with NE200, NE150, NE85, and NE50 Measured at 0.85 V.

Each amperometric SEE data with NE200, NE150, NE85, and NE50 measured at 0.85 V was further analyzed to construct the charge distribution curves (N = 4). Integrated current spikes in amperometric SEE with NE200, NE150, NE85, and NE50 exhibit less than ~10 fC as the highest frequency in the charge distributions over collision events (Figure S6 and S7), indicating insufficient electrolysis of Fc inside NEs due to the tunneling barrier at the NE interface.

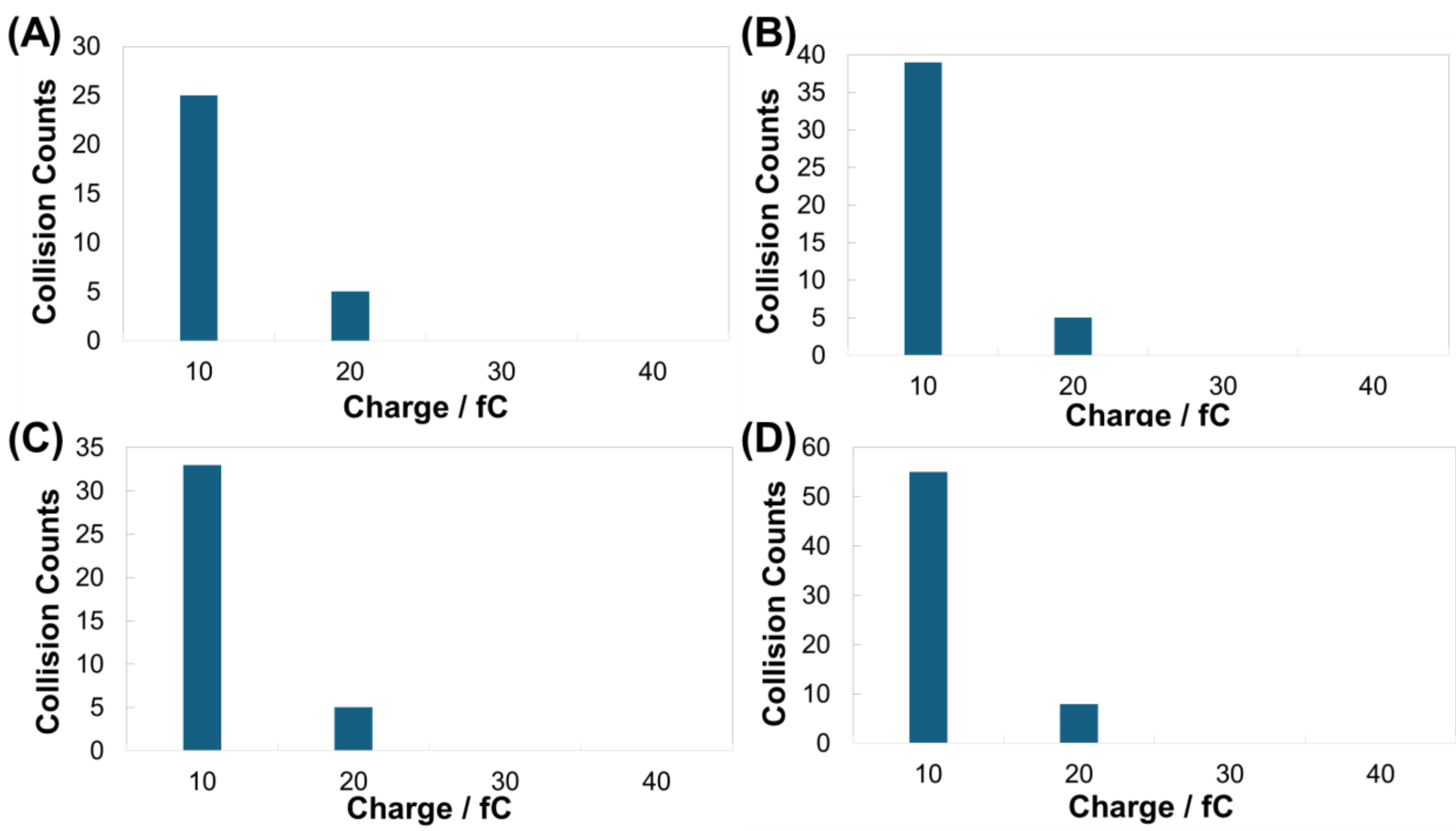

Figure S7. Charge distribution curves from the integrated current spikes in amperometric SEE data measured at 0.85 V under insufficient electrolysis condition (A) NE200, (B) NE150, (C) NE85, (D) NE50 (Corresponding amperometric SEE data are shown in Figure S6).

9. Fitting *i-t* Decay with Bulk Electrolysis Model.

Current decays in each current spike follow the bulk-electrolysis model with showing exponential decay with time (representative simulations with bulk-electrolysis model are shown in the inset (red open circles) in Figure 4A-E). It should be noted that the entire ~300 zmoles of Fc in a single NE is completely electrolyzed, thus depleted within ~ 300 ms upon a single collision with a Pt UME owing to the highly enhanced mass transfer and the high anodic overpotentials. Upon a collisional event, the contact point between a NE and a Pt UME can be considered as an effective inlaid disk electrode for Fc electrolysis inside a NE, of which radius ranges smaller than tens of picometer. For example, mass transfer rate ($m_{Fc} = D_{Fc}/r$) is promoted up to c.a. 50 cm/s with diffusion coefficient of Fc inside a NE, $D_{Fc} = 10^{-7}$ cm^2/s [S4] and a radius of the contact point (an effective fitting parameter), r = 19 pm. If a large ratio between an electrode area (A) and a volume of a cell (V) is fulfilled to enable highly efficient electrolysis, a controlled-potential bulk electrolysis gives exponential current decay with time and eventually attain the background current level. [S7,S8]

$$i(t) = i(0)\exp(-pt) \qquad \text{(S2)}$$

where $p = m_{Fc}A/V$, $i(0)$ is initial current (or, peak current) in current decay, t is time. Subsequently, the duration of the electrolysis for a given condition as,

$$\frac{-p}{2.3}t = \log\left[\frac{i(t)}{i(0)}\right] \qquad \text{(S3)}$$

Accordingly, 99 % completion of electrolysis needs t as $4.6/p$. In typical bulk electrolytic cell under vigorous stirring, $m \approx 10^{-2}$ cm/s with A (cm^2) $\approx V$ (cm^3), $p \approx 10^{-2}$ s^{-1}, so 99 % electrolysis requires ~460 s or ~7.7 min. In this SEE setup, owing to highly enhanced m_{Fc} up to c.a. 50 cm/s, the electrolysis can be kinetically controlled by the inherent ET rate, k_f (see the equation 9 in the main text), thereby achieving $p \approx k_f A/V_{NE} \approx 9.1$ s^{-1} with k_f = ~23 cm/s, $A/V_{NE} = 0.4$ ($A = 1.1 \times 10^{-17}$ cm^2, $V_{NE} = 2.8 \times 10^{-17}$ cm^3). Correspondingly, 99 % electrolysis in a NE could be completed within ~500 ms, which is reasonably consistent with the duration time of current spikes in our amperometric SEE data. Notably, the current density estimated from $i(0)$ at each current spike is ~12 kA/cm^2 (e.g., $i(0)$ = 0.135 pA, $A = 1.1 \times 10^{-17}$ cm^2 with a contact radius = 19 pm), which is 5 orders of magnitude higher than the current density in a highly efficient bulk electrolytic cell. Such a high current density enables the complete electrolysis of Fc in a NE less than 500 ms.

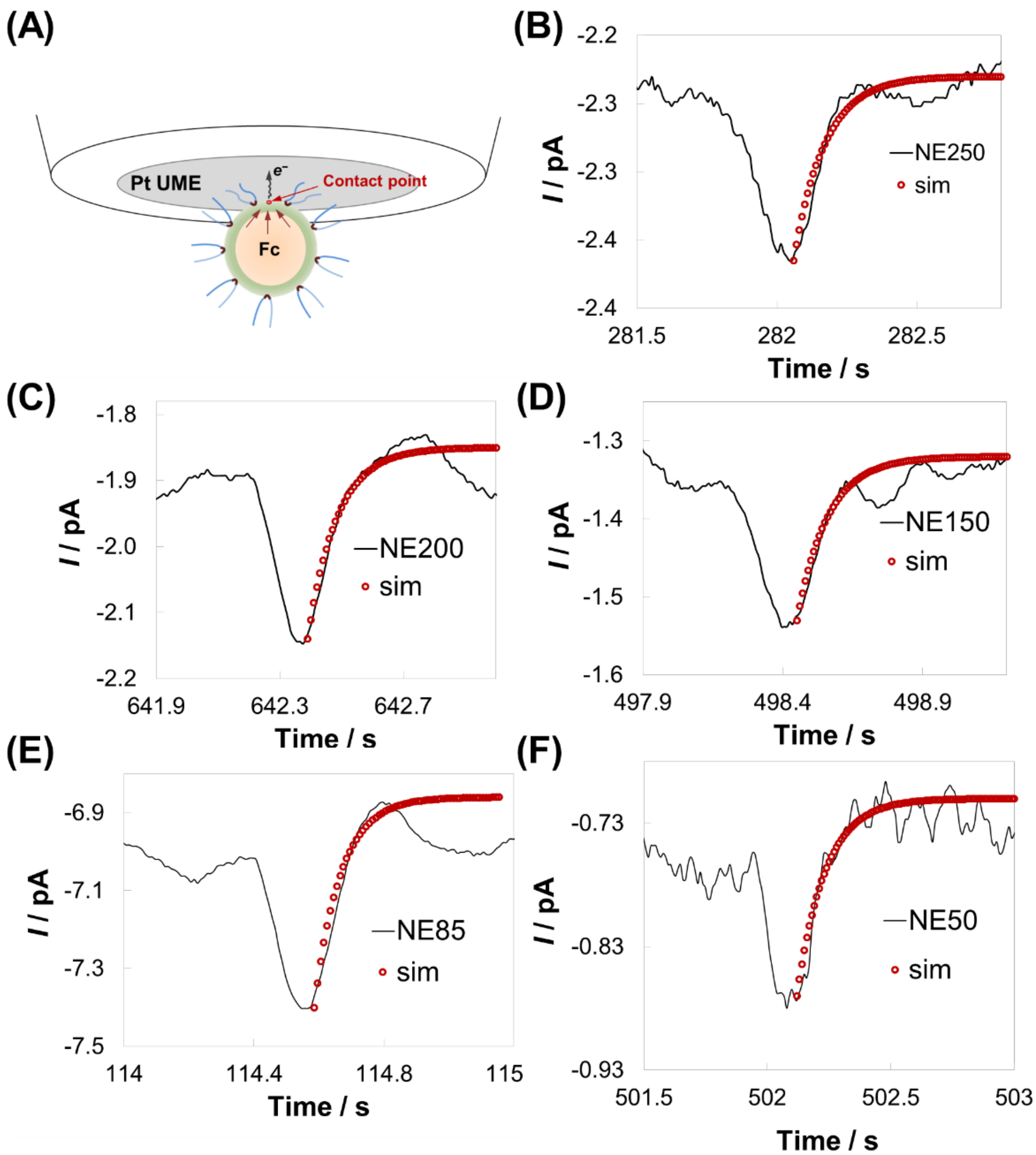

Figure S8. (A) Schematic illustration of a contact between a NE and a Pt UME during amperometric SEE, (B)-(F) Magnified current spikes in *i*-*t* curves of amperometric SEE shown in Figure 4A-E insets, respectively. The experimental curves (black solid lines) are fitted with simulated *i*-*t* behavior for the first order homogeneous electrolysis reaction (i.e., bulk electrolysis model) shown by red open circles.

10. Amperometric SEE data with Sampling Time 1.6 ms.

In amperometric SEE measurements, we tested sampling intervals of 1.6 ms, and confirmed the consistent responses with 10 ms sampling time. With NE250 and a 0.85 V anodic potential, a series of distinctly sharp current spikes were observed, and current spikes 3 times larger than the background noise level were analyzed and integrated to construct charge distribution curves (Figure S9). Each current spike followed bulk-electrolysis behavior, where current decays are well fitted with the simulated *i-t* curves using nearly same parameters as the data with 10 ms sampling time (Figure S9B-S9D and Figure S8, respectively). Also, the charge distribution curves from integrated current spikes exhibited 20–30 fC as the highest frequency in the charge distributions over collision events (Figure S9E), which is consistent with the result of amperometric SEE measurements with 10 ms sampling time (Figure S5). Considering these consistencies in current decays and the charge-distributions, we confirmed that monitoring NE collision events is nearly independent within the range of sampling time 1.6 – 10 ms.

In fact, nano or microemulsion collisional events spanning a few hundred ms are much slower processes than metal-nanoparticle collisions onto a microelectrode with tens of μs duration time.[S7,S9, S10] Hence, sampling times of 1.6 - 10 ms used in this work would hardly distort the inherent responses in NE collisions onto a Pt UME. Overall, 10 ms sampling intervals were selected for both accurate response shapes in current decays and reasonably low background noise in amperometric SEE measurements. In the theoretical fitting with bulk-electrolysis model, the instrument response function was not considered. Even if some delay is anticipated by the instrument response function, it would be still exponentially decaying with exhibiting either underestimated or overestimated electrolysis rate, while the integrated charge amounts should be independently equivalent. The correction from instrument response function would be considered in the future work.

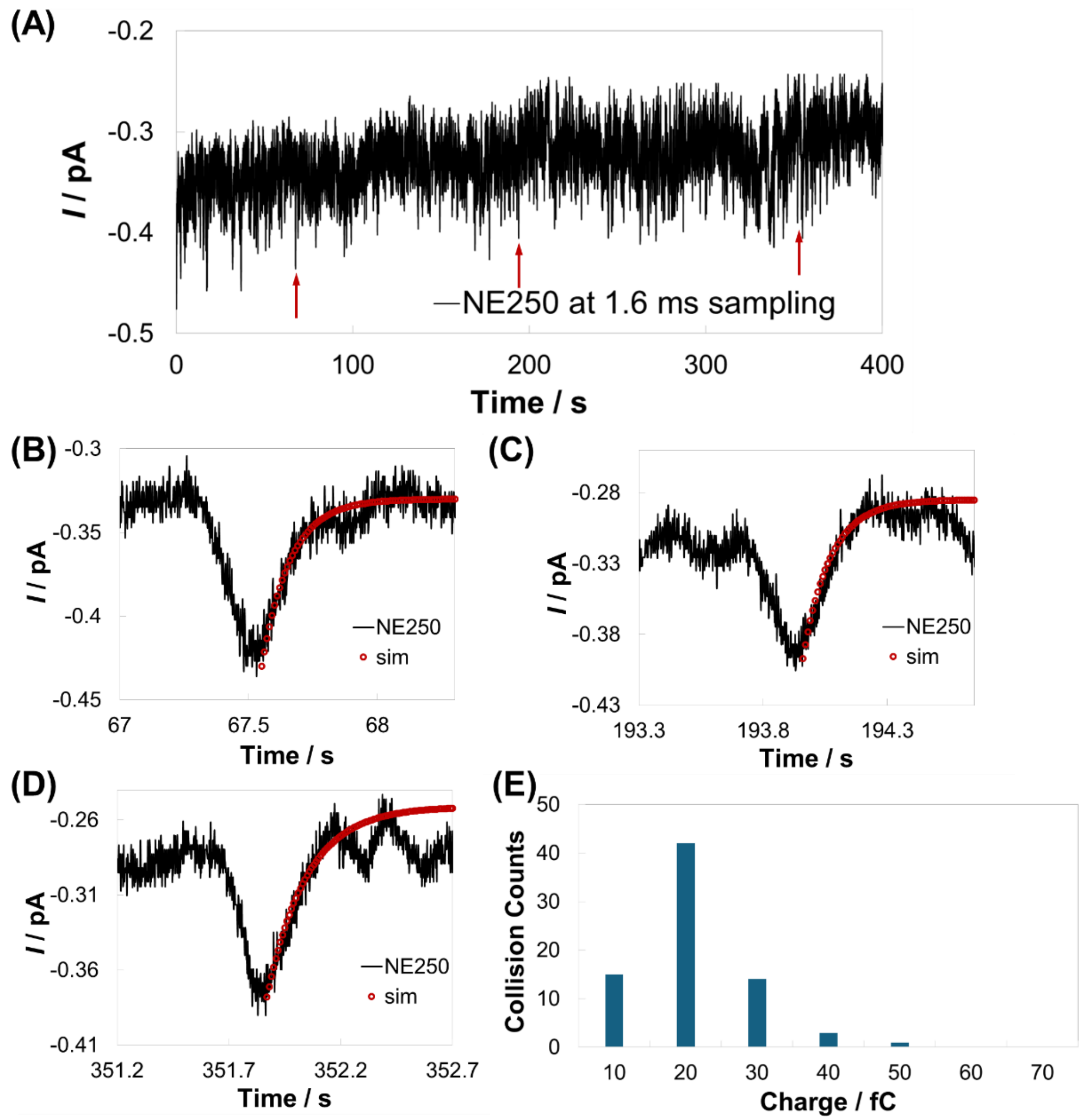

Figure S9. (A) *i*-*t* curve during amperometric SEE with NE 250 under anodic potential at 0.85 V vs Pt QRE and a sampling time 1.6 ms. (B)-(D) Magnified current spikes in *i*-*t* curve of amperometric SEE in Figure S8A. The experimental curves (black solid lines) are fitted with simulated *i*-*t* behavior with bulk electrolysis model (equation S2) shown by red open circles. (E) Charge distribution curves from the integrated current spikes in Figure S9A.

11. COMSOL Multiphysics Simulation Model.

The voltammetry diffusion problem with interfacial ET at the contact between a NE and a Pt UME is solved differential equation in cylindrical coordinates.

$$O + e \underset{k_b}{\overset{k_f}{\rightleftarrows}} R \quad \text{(S4)}$$

$$\frac{\partial c_1}{\partial t} = D_1 \left(\frac{\partial^2 c_1}{\partial r^2} + \frac{1}{r}\frac{\partial c_1}{\partial r} + \frac{\partial^2 c_1}{\partial z^2}\right) \quad \text{(S5)}$$

$$\frac{\partial c_2}{\partial t} = D_2 \left(\frac{\partial^2 c_2}{\partial r^2} + \frac{1}{r}\frac{\partial c_2}{\partial r} + \frac{\partial^2 c_2}{\partial z^2}\right) \quad \text{(S6)}$$

where t is time, r and z are coordinates in parallel and normal directions to the Pt UME interface, respectively. $c_1 (r, z, t)$ and $c_2 (r, z, t)$ are the concentrations of Fc in oxidized and reduced forms, respectively. Herein, we simplify the geometry of a NE contacted on a Pt UME, where a NE is considered as a rectangular geometry with a particular radius of contact point with a Pt UME. Also, homogeneous NE interface is assumed, where the effective interfacial layer thickness determines the overall ET rates. Except the contact point between a NE and a Pt UME, no electron transfer is considered through the insulating NE surface. The variables and the geometry are introduced as follows (see Figure S10):

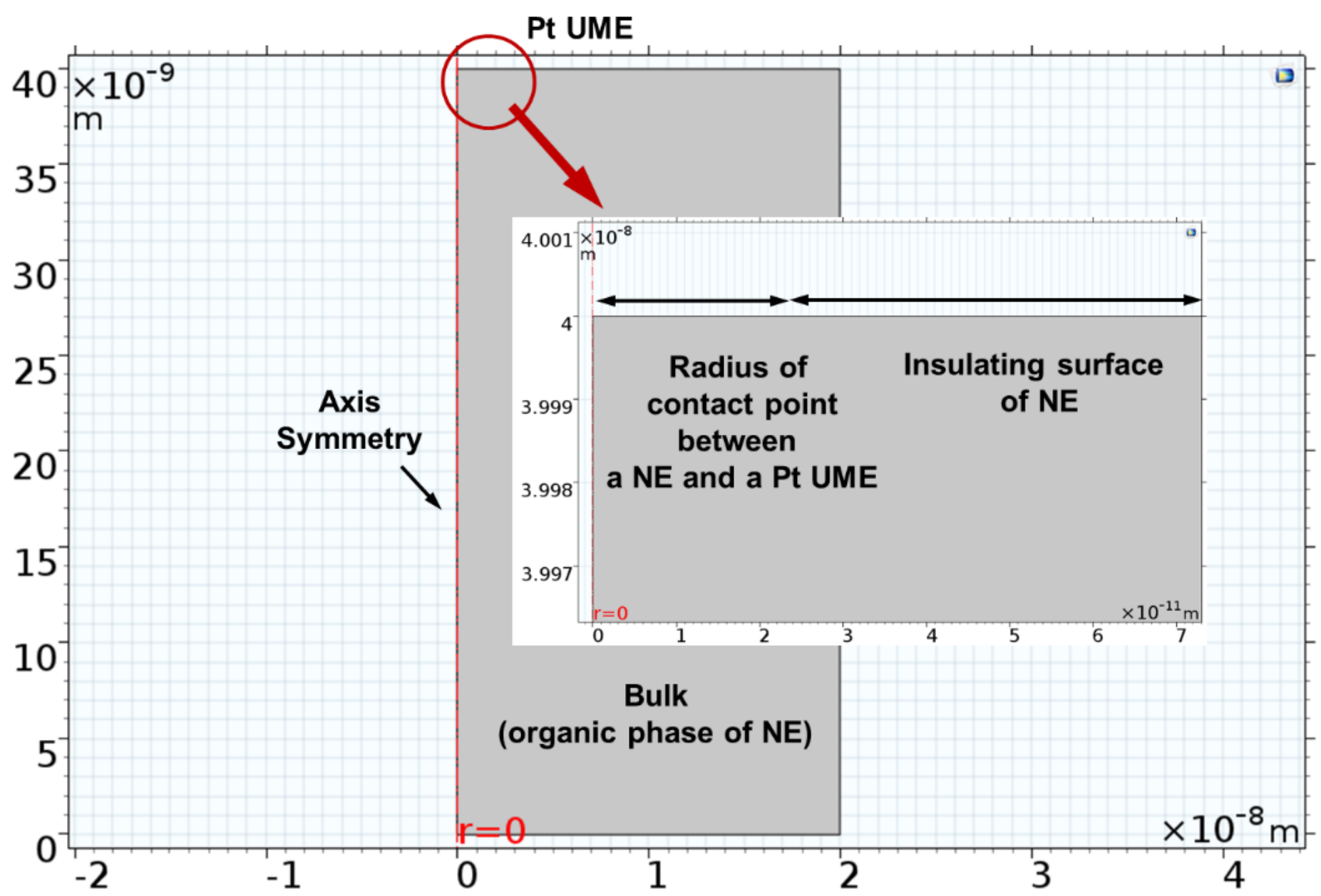

Figure S10. Schematic view of geometry in finite element simulation with COMSOL Multiphysics.

radius = a contact radius between a NE and a Pt UME

D = diffusion coefficient of Fc^+/Fc in NE organic phase

Width = simulation space limit in the radial direction

Height = simulation space limit in the vertical direction

$C1(r, z, \tau)$ = the concentrations of Fc^+ in NE organic phase

$C2(r, z, \tau)$ = the concentrations of Fc in NE organic phase

$C1_{ini}$ = initial concentration of Fc+ inside a NE

n = charge of Fc^+

E_{ini} = initial potential in voltammetry

E_{fin} = switching potential in voltammetry

E^0 = the formal potential of Fc^+/Fc redox reaction ($= E^{0\prime}$)

k_{bare} = formal rate constant for nonadiabatic reactions at the electrode surface (= $k^{0'}{}_b$ in eq 9)

al = the transfer coefficient (α)

v = scan rate (V/s)

tau = ($E_{fin} - E_{ini}$)/v (= τ)

F = 96485 C/mol

beta = tunneling distance decay constant, 3 nm^{-1}

thickness = effective interfacial layer thickness

k^0 = distance-dependent formal rate constant, k_{bare}*exp(-1*beta*thickness) (= $k^{0'}(d)$ in eq 10)

The boundary condition at the NE interface is given by

$$D_1 \left[\frac{\partial c_1(r,z,t)}{\partial z}\right]_{z=0} = D_2 \left[\frac{\partial c_2(r,z,t)}{\partial z}\right]_{z=0} = -k_f c_1(r,0,t) + k_b c_2(r,0,t) \qquad \text{(S7)}$$

where k_f and k_b are the heterogeneous ET rate constants for forward and reverse transfer, respectively (see eq S8 and S9).

The rate constants are given by the Butler-Volmer type relation as,

$$k_f = k^0 exp\left[-\frac{\alpha nF(E-E^{0'})}{RT}\right] \quad \text{(S8)}$$

$$k_b = k^0 exp\left[\frac{(1-\alpha)nF(E-E^{0'})}{RT}\right] \quad \text{(S9)}$$

where k^0 is the distance-dependent formal rate constant, α is the transfer coefficient, E is the applied potential at a Pt UME, and $E^{0'}$ is the formal potential of Fc^+/Fc redox reaction. In voltammetry, the potential is swept linearly at the rate of v from the initial potential, E_{ini} to finial potential, E_{fin}, and the sweep direction is reversed at E_{fin}. The potential wave is expressed as,

$$E = E_{ini} + \frac{2(E_{fin}-E_{ini})}{\pi} sin^{-1}\left\{sin\left[\frac{\pi vt}{2(E_{fin}-E_{ini})}\right]\right\} \quad \text{(S10)}$$

The initial conditions in the NE organic phase (i.e., bulk) are given by

$$c_1(r, z, 0) = 15.7\ M \quad \text{(S11)}$$ [S4]

$$c_2(r, z, 0) = 0\ M \quad \text{(S12)}$$

The other boundary conditions are defined in the COMSOL model report.

Herein, D was determined by Stokes-Einstein relation,[S4]

$$D = \frac{k_B T}{6\pi\eta r} \quad \text{(S13)}$$

where k_B is Boltzmann constant, T is the temperature, η is the viscosity of solvent, and r is a radius of Fc^+/Fc.

The kinetic parameters, k^0, α, and the effective interfacial layer thickness (denoted as *thickness* in COMSOL simulation) were determined via the theoretical simulation. Since interfacial thickness determines k^0 and the overall shape and steepness of voltammograms, *thickness* was sequentially varied for the voltammetric fitting and the determination of kinetic parameters. We determined α by deviating it from 0.5 to finely fit the experimental voltammograms. The values of α = 0.43 – 0.5 showed a good agreement with the experimental voltammograms, which are nearly close to 0.5, indicating the one-step tunneling in the interfacial ET reaction.

More detailed report of this simulation is in supporting information II.

12. Dependence of Simulated Voltammograms on α Values.

In voltammetric fitting, the value of α is critical to determine the shape of simulated voltammograms, thereby fitting simulated results with experimental voltmmograms. Herein, we simulated a series of voltammograms with various α ranging from 0.2 to 0.8, and compared these curves to show how much each curve is deviated from the best-fit result with α = 0.5 and the particular interfacial thickness, e.g., 30 Å (brown open circles in Figure S11, equivalent to brown open circles in Figure 5B).

In Figure S11A, a series of voltammograms with $k^{0'}_{bare}$ = 22.7 cm/s and effective interfacial layer thickness 30 Å were simulated with varying α within the range of 0.2 – 0.8, where all curves were distinctly different. All currents are normalized by the steady-state current with α = 0.5. In Figure S11B, an effective interfacial layer thickness was modulated within 2 - 20 Å with α = 0.2 to apparently overlap with the best-fit result (brown open circles) of α = 0.5 and the interfacial thickness 30 Å, where none of interfacial layer thickness with α = 0.2 exhibited a good agreement with the best-fit curve. In Figure S11C, $k^{0'}_{bare}$ as well as interfacial layer thickness was further changed to simulate a voltammogram close to the best-fit curve. At most, the curve with $k^{0'}_{bare}$ = 0.5 cm/s, 0.1 Å interfacial layer thickness, and α = 0.2 barely matched with the best-fit (grey open circles in Figure S11C). With α = 0.3, parameters of $k^{0'}_{bare}$ = 0.2 cm/s and 0.01 Å interfacial layer thickness results in a nearly overlapped voltammogram with the best-fit, while an unreasonably small $k^{0'}_{bare}$ for outer-sphere redox mediators [S11,S12,S13]and an unrealistically thin interfacial layer thickness suggest unreliable fitting results. Overall, the series of fitting results with α values deviated from α =0.5 clearly indicates the stiffness of α ranges determined by voltammetric fitting.

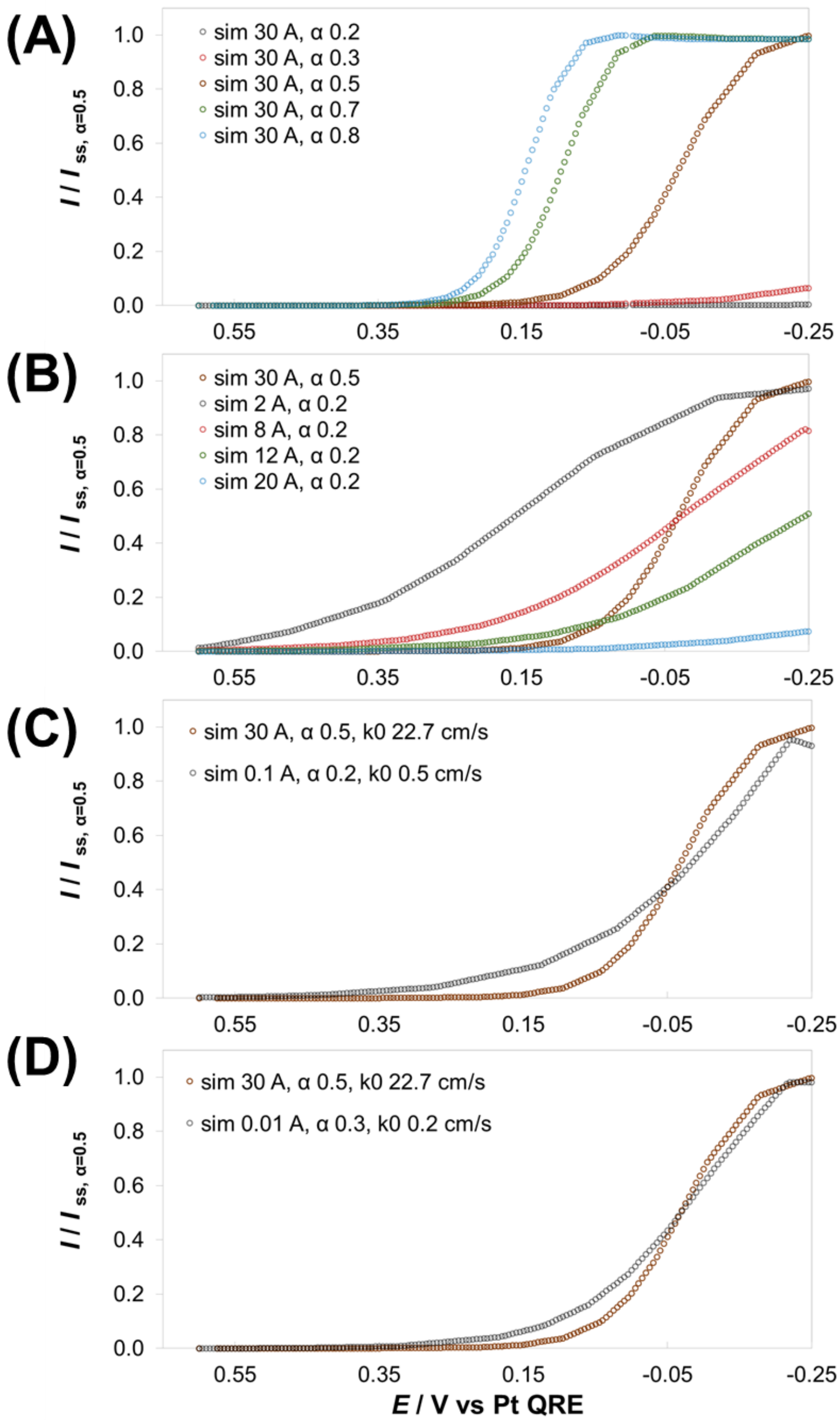

Figure S11. Voltammograms simulated with (A) various α values ranges from 0.2 to 0.8, $k^{0'}_{bare}$ = 22.7 cm/s and effective interfacial layer thickness 30 Å, (B) an effective interfacial layer thickness within 2 - 20 Å with α = 0.2. (C) $k^{0'}_{bare}$ = 0.5 cm/s, 0.1 Å interfacial layer thickness, and α = 0.2. (D) $k^{0'}_{bare}$ = 0.2 cm/s and 0.01 Å interfacial layer thickness with α = 0.3. All simulated voltammograms are compared to the best-fit result (brown open circles) of α = 0.5 and the interfacial thickness 30 Å.

13. Estimation of a Steady-State Current in MSEE

According to equation S14, a steady-state current can be estimated as ~0.3 pA for voltammograms in Figure 4F, where NEs are in contact with a Pt with a contact radius a = 5.7 pm (an effective fitting parameter), and undergo Fc^{+} reduction reaction with n (transferred electron number, n = 1 for Fc/Fc^{+}), RG factor of x = 1.00 and other parameters the same as boundary conditions used in the COMSOL simulation.

$$i_{lim} = 4xnFD_{Fc}C_{Fc}a \qquad \text{(S14)}$$

where F is the Faraday constant (= 96485 C/mol), and D_i (= 10^{-7} cm^2/s), and C_i (= ~15 M) are a diffusion coefficient and a concentration of Fc in the NE organic phase as a bulk solution, respectively.[S4]

14. The Effect of Contact Radius, an Effective Fitting Parameter on the Estimation of Interfacial Layer Thickness

When the contact radius varies between 5.7 pm and 19 pm in the theoretical simulation, resultant voltammograms shift within ~10 mV, which corresponds to deviations at ~1 Å to maximum 2 Å level depending on the range of interfacial layer thickness (Figure S12). Accordingly, in our theoretical analysis, the variation of a contact radius within 5.7 – 19 pm would lead to the uncertainty at 1-2 Å level in the estimation of interfacial layer thickness at NEs. In Figure S12, normalized voltammograms with contact radii, 5.7 and 19 pm are compared at different interfacial layer thickness within 0.1 – 30.0 Å, which exhibits slight kinetic difference.

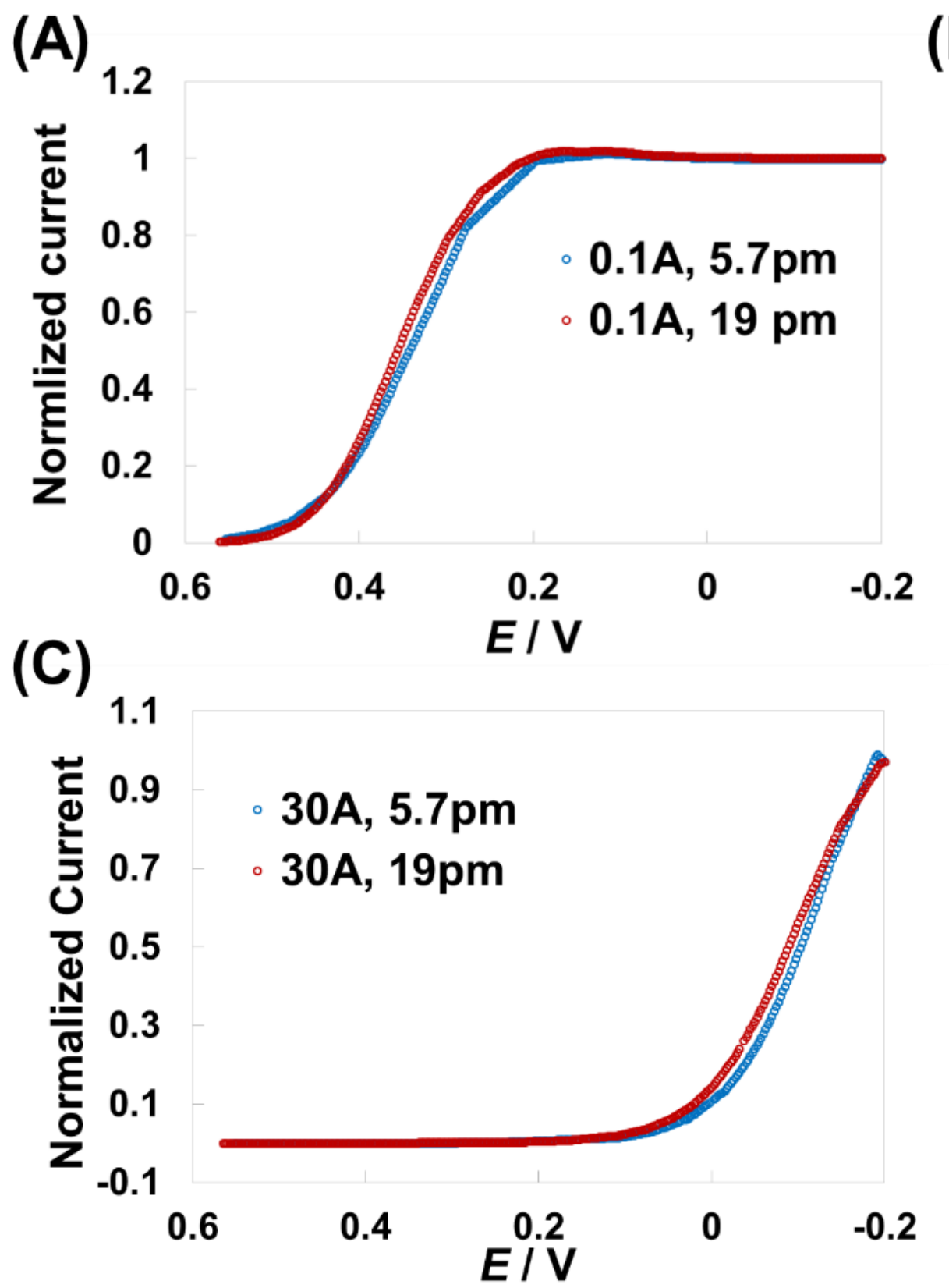

Figure S12. Comparison of simulated voltammograms with contact radii, 5.7 pm and 19 pm at different interfacial layer thickness of (A) 0.1 Å, (B) 17 Å, (C) 30 Å. Currents are normalized with the each limiting-current, respectively.

References.

S1. Roselynn Cordero; Ali Jawaid; Ming-Siao Hsiao; Zoë Lequeux; Richard A. Vaia; Christopher K. Ober, Mini Monomer Encapsulated Emulsion Polymerization of PMMA Using Aqueous ARGET ATRP, ACS Macro Lett. 2018, 7, 459–463, doi.org/10.1021/acsmacrolett.8b00038

S2. Dehvari, K.; Lin, K.-S.; Wang, S. S.-S., Structural Characterization and Adsorption Properties of Pluronic F127 Onto Iron Oxides Magnetic Nanoparticles, Nanosci. Nanotechnol., 2014, 14, 2361-2367, doi.org/10.1166/jnn.2014.8537

S3. Tan, J.S.; Butterfield, D.E.; Voycheck, C.L.; Caldwell, K.D. ; Li, J.T., Surface modification of nanoparticles by PEO/PPO block copolymers to minimize interactions with blood components and prolong blood circulation in rats, Biomaterials, 1993, 14, 823-833, 10.1016/0142-9612(93)90004-L

S4. Sabaragamuwe, S.G.; Conti, D.; Puri, S.R.; Andreu, I.; Kim, J., Single-Entity Electrochemistry of Nanoemulsion: The Nanostructural Effect on Its Electrochemical Behavior. *Anal. Chem*., **2019**, 91, 9599-9607, doi.org/10.1021/acs.analchem.9b00920

S5. Hunter R.J., Zeta potential in colloid science, principles and applications, colloid Science A series of monographs, Academic press, San Diego, 1988, p220-239

S6. Cornut, R.; Lefrou, C., A unified new analytical approximation for negative feedback currents with a microdisk SECM tip. *J. Electroanalytical. Chem.*, **2007**, 608, 59-66, 10.1016/j.jelechem.2007.05.007

S7. Kim, B.K.; Boika, A.; Kim, J.; Dick, J.E.; Bard, A.J., Characterizing Emulsions by Observation of Single Droplet Collisions- Attoliter Electrochemical Reactors. *J. Am. Chem. Soc.*, **2014**, 136, 4849-4852, doi.org/10.1021/ja500713w

S8. Bard, A. J.; Faulkner, L. R., “Electrochemical Methods: Fundamental and Applications”, **2004**, 2nd Ed., John Wiley & Sons INC.

S9. Kim, B-K.; Kim, J.; Bard, A.J., Electrochemistry of a Single Attoliter Emulsion Droplet in Collisions. *J. Am. Chem. Soc.*, **2015**, 137, 2343-2349, doi.org/10.1021/ja512065n

S10. Robinson, D.A.; Edwards, M.A.; Ren, H.; White, H.S., Effects of Instrumental Filters on Electrochemical Measurement of Single-Nanoparticle Collision Dynamics. *Chem. Electro. Chem*. **2018**, 5, 3059- 3067, doi.org/10.1002/celc.201800696

S11. Sun, P.; Mirkin, M. V., Kinetics of Electron-Transfer Reactions at Nanoelectrodes. *Anal. Chem.* **2006**, 78, 6526– 6534, DOI: 10.1021/ac060924q

S12. Nioradze, N.; Chen, R.; Kurapati, N.; Khvataeva-Domanov, A.; Mabic, S.; Amemiya, S., Organic Contamination of Highly Oriented Pyrolytic Graphite As Studied by Scanning Electrochemical Microscopy. *Anal. Chem.* **2015**, 87, 4836– 4843, DOI: 10.1021/acs.analchem.5b00213

S13. Kim, J.; Bard, A.J., Electrodeposition of Single Nanometer-Size Pt Nanoparticles at a Tunneling Ultramicroelectrode and Determination of Fast Heterogeneous Kinetics for $Ru(NH_3)_6^{3+}$ Reduction. *J. Am. Chem. Soc.* **2016**, 138, 975-979, doi.org/10.1021/jacs.5b11655
