# Supplementary material for: In Situ Study of Electron-Tunneling through Dynamic Interface at Individual Nanoemulsions by Multimodal Single-Entity Electrochemistry
Source: J Phys Chem B. 2026 Oct 21;130(44):11931–41. doi: 10.1021/acs.jpcb.6c04932 (PMC13746782; doi:10.1021/acs.jpcb.6c04932)
Supplement: Supplementary file 2 [file jp6c04932_si_002.pdf]

Supporting information II

In Situ Study of Electron-Tunneling through Dynamic Interface at Individual Nanoemulsions by Multimodal Single-Entity Electrochemistry

Anuradha Khatri Chhetri,[a] Mandip Adhikari,[a] and Jiyeon Kim[a,*]

[a] Department of Chemistry, University of Rhode Island, Kingston, Rhode Island, 02881, United States

* To whom all correspondence should be addressed. E-mail: jkim25@uri.edu.

Fc+ reduction-tunneling, thickness 0.1A

Report date	Jun 18, 2026 6:44:37 PM

Contents

1. Global Definitions **4**

1.1. Parameters 1 4

1.2. Variables 5

2. Model 1 ..... **6**

2.1. Definitions 6

2.2. Geometry 1 8

2.3. Transport of Diluted Species 9

2.4. Mesh 1 21

3. Study 1 ..... **25**

3.1. Time Dependent 25

3.2. Solver Configurations 25

4. Results **29**

4.1. Data Sets 29

4.2. Plot Groups 30

1 Global Definitions

Date	Jan 30, 2026 11:50:11 AM

GLOBAL SETTINGS

Name	Fc+ reduction-tunneling, thickness 0.1A.mph
Path	C:\Users\Jkim\Documents\JKim\Jay only-simulation\2026\Multimodal SEE\Fc+ reduction-tunneling, thickness 0.1A.mph
Version	COMSOL Multiphysics 5.3a (Build: 229)

USED PRODUCTS

COMSOL Multiphysics
Chemical Reaction Engineering Module

1.1 PARAMETERS 1

PARAMETERS

Name	Expression	Value	Description
width	20[nm]	2E−8 m	
height	40[nm]	4E−8 m	
radius	5.7[pm]	5.7E−12 m	
D	1E-7[cm^2/s]	1E−11 m^2/s	
c1ini	15.7[mol/L]	15700 mol/m^3	
F	96485[C/mol]	96485 C/mol	
n	1	1	
f	38.92[V^(-1)]	38.92 1/V	
Eo	0.50[V]	0.5 V	
kbare	22.7[cm/s]	0.227 m/s	
al	0.5	0.5	
v	0.025[V/s]	0.025 V/s	
tau	(Eini - Efin)/v	16 s	
Eini	0.6[V]	0.6 V	
Efin	0.2[V]	0.2 V	
beta	3 [nm^(-1)]	3E9 1/m	
ko	kbare*exp(-1*beta*thickness)	0.22029 m/s	
thickness	0.1E-10 [m]	1E−11 m	

1.2 VARIABLES

1.2.1 Variables 1

SELECTION

Geometric entity level	Entire model

Name	Expression	Unit	Description
kft	ko*exp((-al)*n*f*(E-Eo))	m/s	
kbt	ko*exp((1 - al)*n*f*(E-Eo))	m/s	
E	(sw1*(Eini - v*t)) + (sw2*(Efin + v*(t - tau)))	V	
sw1	flc1hs(1 - t/tau, 0.0000001)		
sw2	flc1hs(t/tau - 1, 0.0000001)		

2 Model 1

SETTINGS

Description	Value
Unit system	Same as global system

2.1 DEFINITIONS

2.1.1 Variables

Variables 2

SELECTION

Geometric entity level	Entire model

Name	Expression	Unit	Description
current	-tipint(n*F*D*2*pi*r*c1z)	A	

2.1.2 Component Couplings

Integration 1

Coupling type	Integration
Operator name	tipint

SELECTION

Geometric entity level	Boundary
Selection	Boundary 3

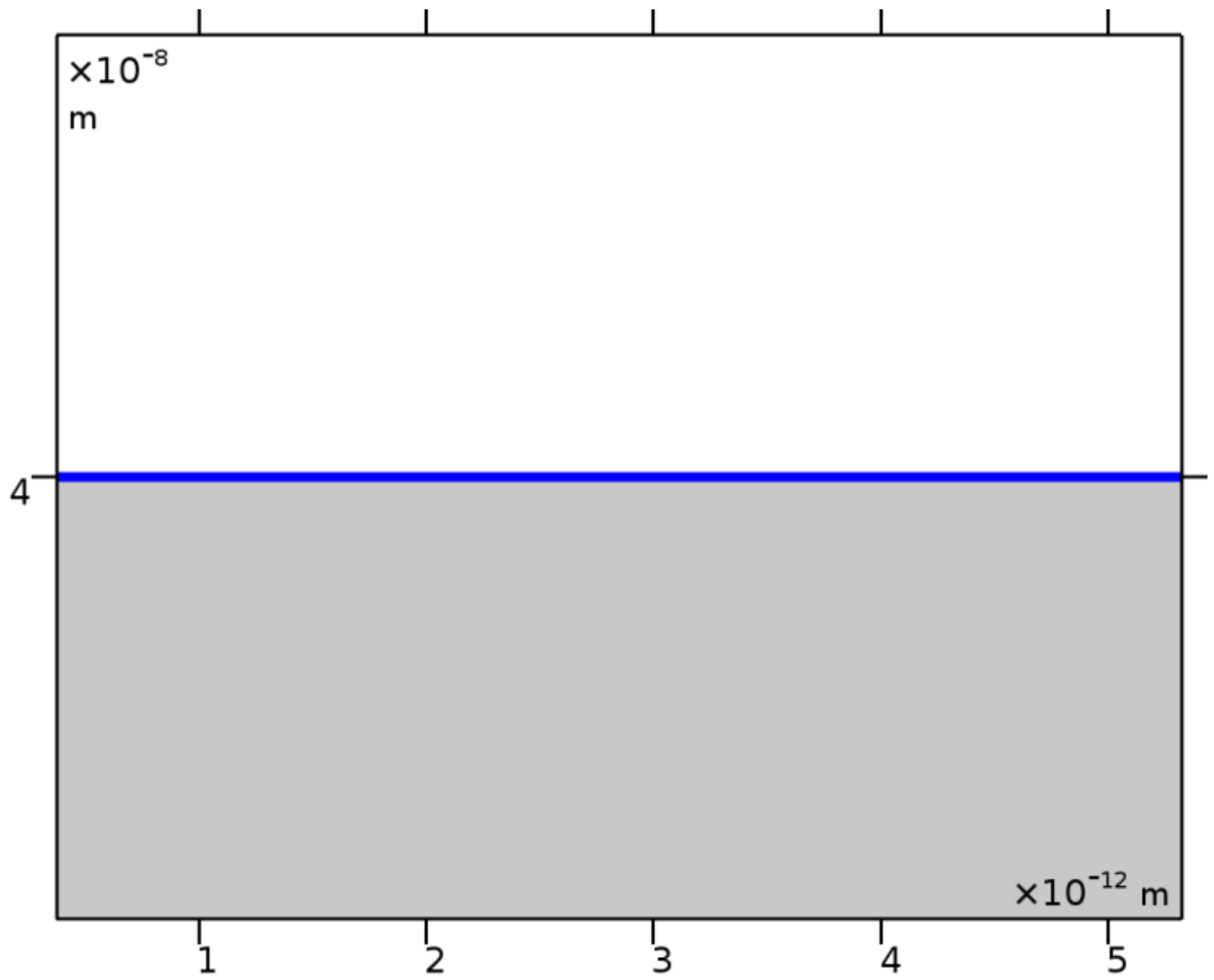

Selection

ADVANCED

Description	Value
Integration order	1

2.1.3 Coordinate Systems

Boundary System 1

Coordinate system type	Boundary system
Tag	sys1

SETTINGS

First	Second	Third
t1	to	n

2.2 GEOMETRY 1

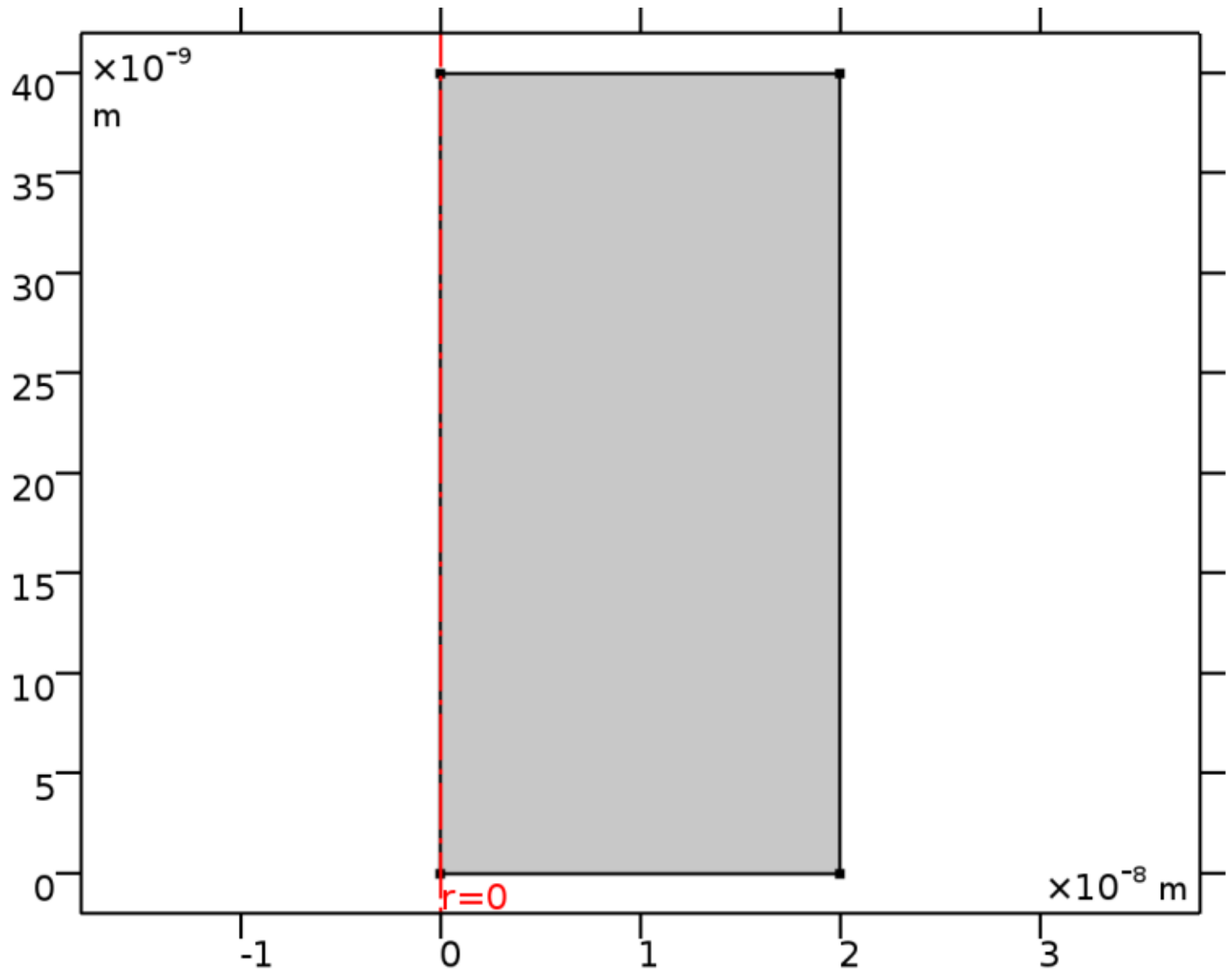

Geometry 1

UNITS

Length unit	m
Angular unit	deg

GEOMETRY STATISTICS

Description	Value
Space dimension	2
Number of domains	1
Number of boundaries	5
Number of vertices	5

2.2.1 Polygon 1 (pol1)

SELECTIONS OF RESULTING ENTITIES

Description	Value
Resulting objects selection	On

SETTINGS

Description	Value
Show in physics	All levels

OBJECT TYPE

Description	Value
Type	Solid

COORDINATES

Description	Value
r	{0, 5.7E-12, 2.0E-8, 2.0E-8, 0}
z	{4.0E-8, 4.0E-8, 4.0E-8, 0, 0}

2.3 TRANSPORT OF DILUTED SPECIES

USED PRODUCTS

COMSOL Multiphysics
Chemical Reaction Engineering Module

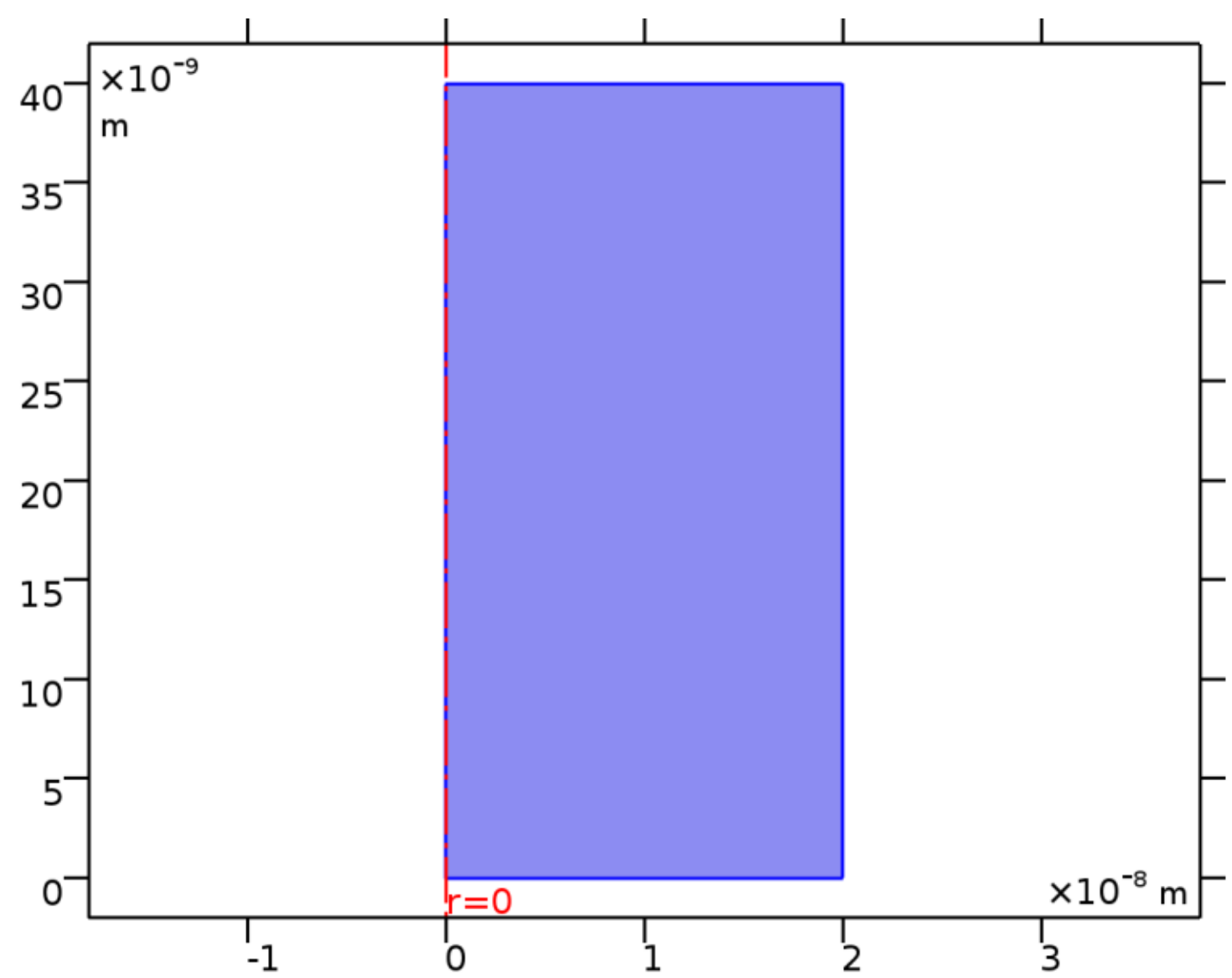

Transport of Diluted Species

SELECTION

Geometric entity level	Domain
Selection	Domain 1

EQUATIONS

$$\frac{\partial c_i}{\partial t} + \nabla \cdot (-D_i \nabla c_i) = R_i$$

$$\mathbf{N}_i = -D_i \nabla c_i$$

2.3.1 Interface settings

Transport mechanisms

SETTINGS

Description	Value
Convection	Off
Migration in electric field	Off
Mass transfer in porous media	Off

2.3.2 Variables

Name	Expression	Unit	Description	Selection	Details
chds.R_c1	0	mol/(m³·s)	Total rate expression	Domain 1	+ operation
chds.R_c2	0	mol/(m³·s)	Total rate expression	Domain 1	+ operation
chds.epsilon_p	1	1	Porosity	Domain 1	
chds.theta	chds.epsilon_p	1	Liquid volume fraction	Domain 1	
chds.av	0	1	Gas volume fraction	Domain 1	
chds.nr	dnr	1	Normal vector, r component	Boundaries 1–5	
chds.nphi	0	1	Normal vector, phi component	Boundaries 1–5	
chds.nz	dnz	1	Normal vector, z component	Boundaries 1–5	
chds.nrmesh	dnrmesh	1	Normal vector (mesh), r component	Boundaries 1–5	
chds.nphimesh	0	1	Normal vector (mesh), phi component	Boundaries 1–5	
chds.nzmesh	dnzmesh	1	Normal vector (mesh), z component	Boundaries 1–5	
chds.nrc	root.nrc/chds.ncLen	1	Normal vector, r component	Boundaries 1–5	
chds.nphic	0	1	Normal vector, phi component	Boundaries 1–5	

Name	Expression	Unit	Description	Selection	Details
chds.nzc	root.nzc/chds.ncLen	1	Normal vector, z component	Boundaries 1–5	
chds.ncLen	sqrt(root.nrc^2+root.nzc^2+eps)	1	Help variable	Boundaries 1–5	

2.3.3 Diffusion

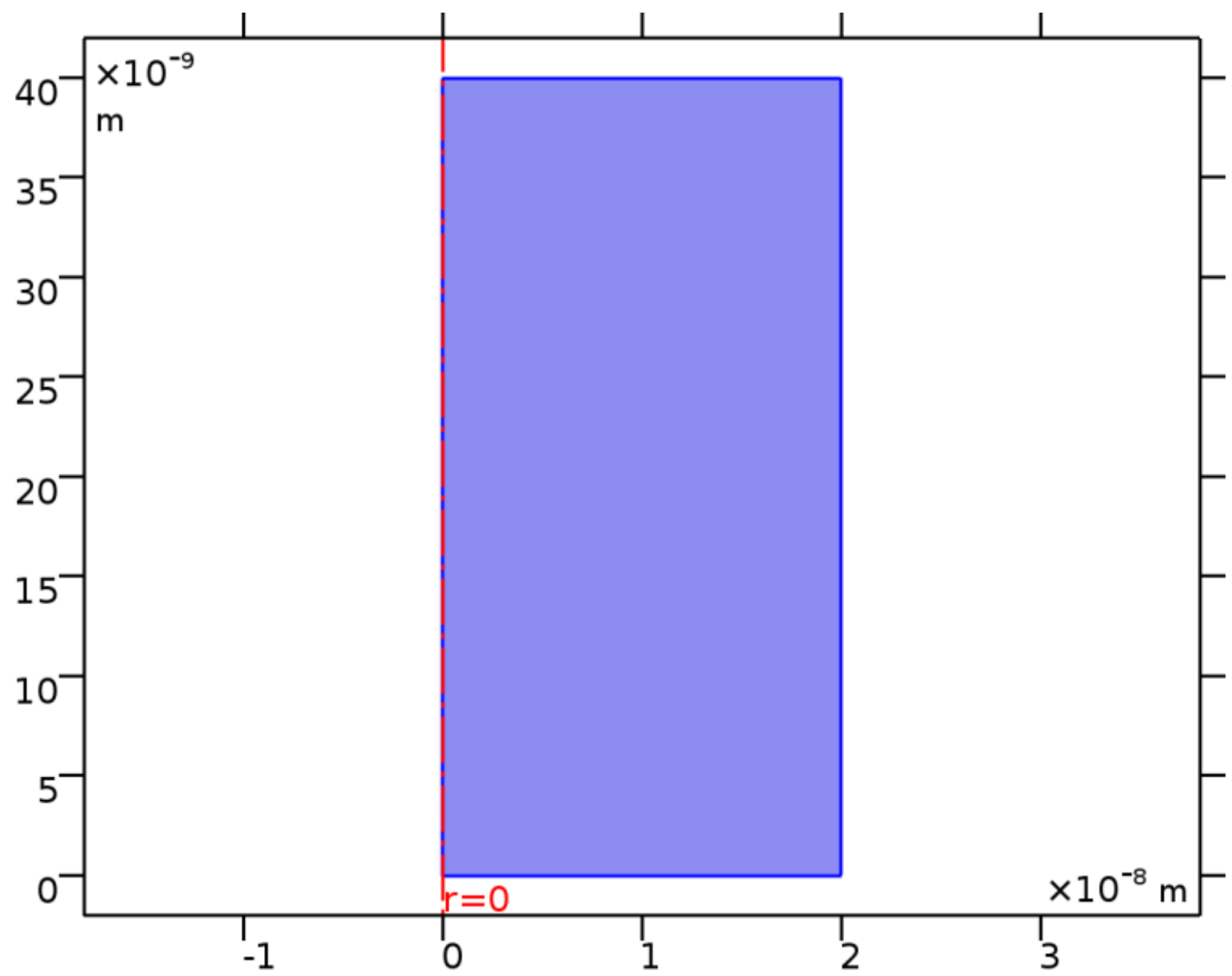

Diffusion

SELECTION

Geometric entity level	Domain
Selection	Domain 1

EQUATIONS

$$\frac{\partial c_i}{\partial t} + \nabla \cdot (-D_i \nabla c_i) = R_i$$

$$\mathbf{N}_i = -D_i \nabla c_i$$

Diffusion

SETTINGS

Description	Value
Material	None
Diffusion coefficient	User defined
Diffusion coefficient	{{D, 0, 0}, {0, D, 0}, {0, 0, D}}
Diffusion coefficient	User defined
Diffusion coefficient	{{D, 0, 0}, {0, D, 0}, {0, 0, D}}

Description	Value
Coordinate system	Global coordinate system
Temperature	User defined
Temperature	293.15[K]

USED PRODUCTS

COMSOL Multiphysics

Variables

Name	Expression	Unit	Description	Selection	Details
domflux.c1r	2*chds.dflux_c1r*pi*r	mol/(m·s)	Domain flux, r component	Domain 1	
domflux.c1z	2*chds.dflux_c1z*pi*r	mol/(m·s)	Domain flux, z component	Domain 1	
domflux.c2r	2*chds.dflux_c2r*pi*r	mol/(m·s)	Domain flux, r component	Domain 1	
domflux.c2z	2*chds.dflux_c2z*pi*r	mol/(m·s)	Domain flux, z component	Domain 1	
chds.ndflux_c1	chds.bndFlux_c1	mol/(m²·s)	Normal diffusive flux	Boundaries 2–5	
chds.ndflux_c2	chds.bndFlux_c2	mol/(m²·s)	Normal diffusive flux	Boundaries 2–5	
chds.ntflux_c1	chds.bndFlux_c1	mol/(m²·s)	Normal total flux	Boundaries 2–5	
chds.ntflux_c2	chds.bndFlux_c2	mol/(m²·s)	Normal total flux	Boundaries 2–5	
chds.bndFlux_c1	if(r>0.001/sqrt(sqrt(mean(emetric2))),-0.5*dflux_spatial(c1)/(pi*r),NaN)	mol/(m²·s)	Boundary flux	Boundaries 1–5	
chds.bndFlux_c2	if(r>0.001/sqrt(sqrt(mean(emetric2))),-0.5*dflux_spatial(c2)/(pi*r),NaN)	mol/(m²·s)	Boundary flux	Boundaries 1–5	
chds.D_c1rr	D	m²/s	Diffusion coefficient, rr component	Domain 1	+ operation
chds.D_c1phir	0	m²/s	Diffusion coefficient, phir component	Domain 1	+ operation

Name	Expression	Unit	Description	Selection	Details
chds.D_c1zr	0	m^2/s	Diffusion coefficient, zr component	Domain 1	+ operation
chds.D_c1rphi	0	m^2/s	Diffusion coefficient, rphi component	Domain 1	+ operation
chds.D_c1phiphi	D	m^2/s	Diffusion coefficient, phiphi component	Domain 1	+ operation
chds.D_c1zphi	0	m^2/s	Diffusion coefficient, zphi component	Domain 1	+ operation
chds.D_c1rz	0	m^2/s	Diffusion coefficient, rz component	Domain 1	+ operation
chds.D_c1phiz	0	m^2/s	Diffusion coefficient, phiz component	Domain 1	+ operation
chds.D_c1zz	D	m^2/s	Diffusion coefficient, zz component	Domain 1	+ operation
chds.D_c2rr	D	m^2/s	Diffusion coefficient, rr component	Domain 1	+ operation
chds.D_c2phir	0	m^2/s	Diffusion coefficient, phir component	Domain 1	+ operation
chds.D_c2zr	0	m^2/s	Diffusion coefficient, zr component	Domain 1	+ operation
chds.D_c2rphi	0	m^2/s	Diffusion coefficient, rphi component	Domain 1	+ operation
chds.D_c2phiphi	D	m^2/s	Diffusion coefficient, phiphi component	Domain 1	+ operation
chds.D_c2zphi	0	m^2/s	Diffusion coefficient, zphi component	Domain 1	+ operation

Name	Expression	Unit	Description	Selection	Details
chds.D_c2rz	0	m^2/s	Diffusion coefficient, rz component	Domain 1	+ operation
chds.D_c2phiz	0	m^2/s	Diffusion coefficient, phiz component	Domain 1	+ operation
chds.D_c2zz	D	m^2/s	Diffusion coefficient, zz component	Domain 1	+ operation
chds.Dav_c1	0.5*(chds.D_c1rr+chds.D_c1zz)	m^2/s	Average diffusion coefficient	Domain 1	
chds.Dav_c2	0.5*(chds.D_c2rr+chds.D_c2zz)	m^2/s	Average diffusion coefficient	Domain 1	
chds.tflux_c1r	chds.dflux_c1r	$mol/(m^2·s)$	Total flux, r component	Domain 1	+ operation
chds.tflux_c1phi	chds.dflux_c1phi	$mol/(m^2·s)$	Total flux, phi component	Domain 1	+ operation
chds.tflux_c1z	chds.dflux_c1z	$mol/(m^2·s)$	Total flux, z component	Domain 1	+ operation
chds.tflux_c2r	chds.dflux_c2r	$mol/(m^2·s)$	Total flux, r component	Domain 1	+ operation
chds.tflux_c2phi	chds.dflux_c2phi	$mol/(m^2·s)$	Total flux, phi component	Domain 1	+ operation
chds.tflux_c2z	chds.dflux_c2z	$mol/(m^2·s)$	Total flux, z component	Domain 1	+ operation
chds.dfluxMag_c1	sqrt(chds.dflux_c1r^2+chds.dflux_c1phi^2+chds.dflux_c1z^2)	$mol/(m^2·s)$	Diffusive flux magnitude	Domain 1	
chds.dfluxMag_c2	sqrt(chds.dflux_c2r^2+chds.dflux_c2phi^2+chds.dflux_c2z^2)	$mol/(m^2·s)$	Diffusive flux magnitude	Domain 1	
chds.tfluxMag_c1	sqrt(chds.tflux_c1r^2+chds.tflux_c1phi^2+chds.tflux_c1z^2)	$mol/(m^2·s)$	Total flux magnitude	Domain 1	
chds.tfluxMag_c2	sqrt(chds.tflux_c2r^2+chds.tflux_c2phi	$mol/(m^2·s)$	Total flux magnitude	Domain 1	

Name	Expression	Unit	Description	Selection	Details
	^2+chds.tflux_c2z^2)				
chds.dflux_c1r	-chds.D_c1rr*c1r-chds.D_c1rz*c1z	mol/(m²·s)	Diffusive flux, r component	Domain 1	
chds.dflux_c1phi	-chds.D_c1phir*c1r-chds.D_c1phiz*c1z	mol/(m²·s)	Diffusive flux, phi component	Domain 1	
chds.dflux_c1z	-chds.D_c1zr*c1r-chds.D_c1zz*c1z	mol/(m²·s)	Diffusive flux, z component	Domain 1	
chds.dflux_c2r	-chds.D_c2rr*c2r-chds.D_c2rz*c2z	mol/(m²·s)	Diffusive flux, r component	Domain 1	
chds.dflux_c2phi	-chds.D_c2phir*c2r-chds.D_c2phiz*c2z	mol/(m²·s)	Diffusive flux, phi component	Domain 1	
chds.dflux_c2z	-chds.D_c2zr*c2r-chds.D_c2zz*c2z	mol/(m²·s)	Diffusive flux, z component	Domain 1	
chds.grad_c1r	c1r	mol/m⁴	Concentration gradient, r component	Domain 1	
chds.grad_c1phi	0	mol/m⁴	Concentration gradient, phi component	Domain 1	
chds.grad_c1z	c1z	mol/m⁴	Concentration gradient, z component	Domain 1	
chds.grad_c2r	c2r	mol/m⁴	Concentration gradient, r component	Domain 1	
chds.grad_c2phi	0	mol/m⁴	Concentration gradient, phi component	Domain 1	
chds.grad_c2z	c2z	mol/m⁴	Concentration gradient, z component	Domain 1	
chds.helem	h	m	Element size	Domain 1	
chds.glim_mass	0.1[mol/m^3]/chds.helem	mol/m⁴	Lower gradient limit	Domain 1	
chds.Res_c1	c1t-chds.R_c1	mol/(m³·s)	Equation residual	Domain 1	
chds.Res_c2	c2t-chds.R_c2	mol/(m³·s)	Equation residual	Domain 1	

Shape functions

Name	Shape function	Unit	Description	Shape frame	Selection
c1	Lagrange (Linear)	mol/m^3	Concentration	Material	Domain 1
c2	Lagrange (Linear)	mol/m^3	Concentration	Material	Domain 1

Weak expressions

Weak expression	Integration order	Integration frame	Selection
2*(-c1t*test(c1)+chds.dflux_c1r*test(c1r)+chds.dflux_c1z*test(c1z))*pi*r	2	Material	Domain 1
2*(-c2t*test(c2)+chds.dflux_c2r*test(c2r)+chds.dflux_c2z*test(c2z))*pi*r	2	Material	Domain 1
2*chds.streamline*(isScalingSystemDomain==0)*pi*r	2	Material	Domain 1
2*chds.crosswind*(isScalingSystemDomain==0)*pi*r	4	Material	Domain 1

2.3.4 Axial Symmetry 1

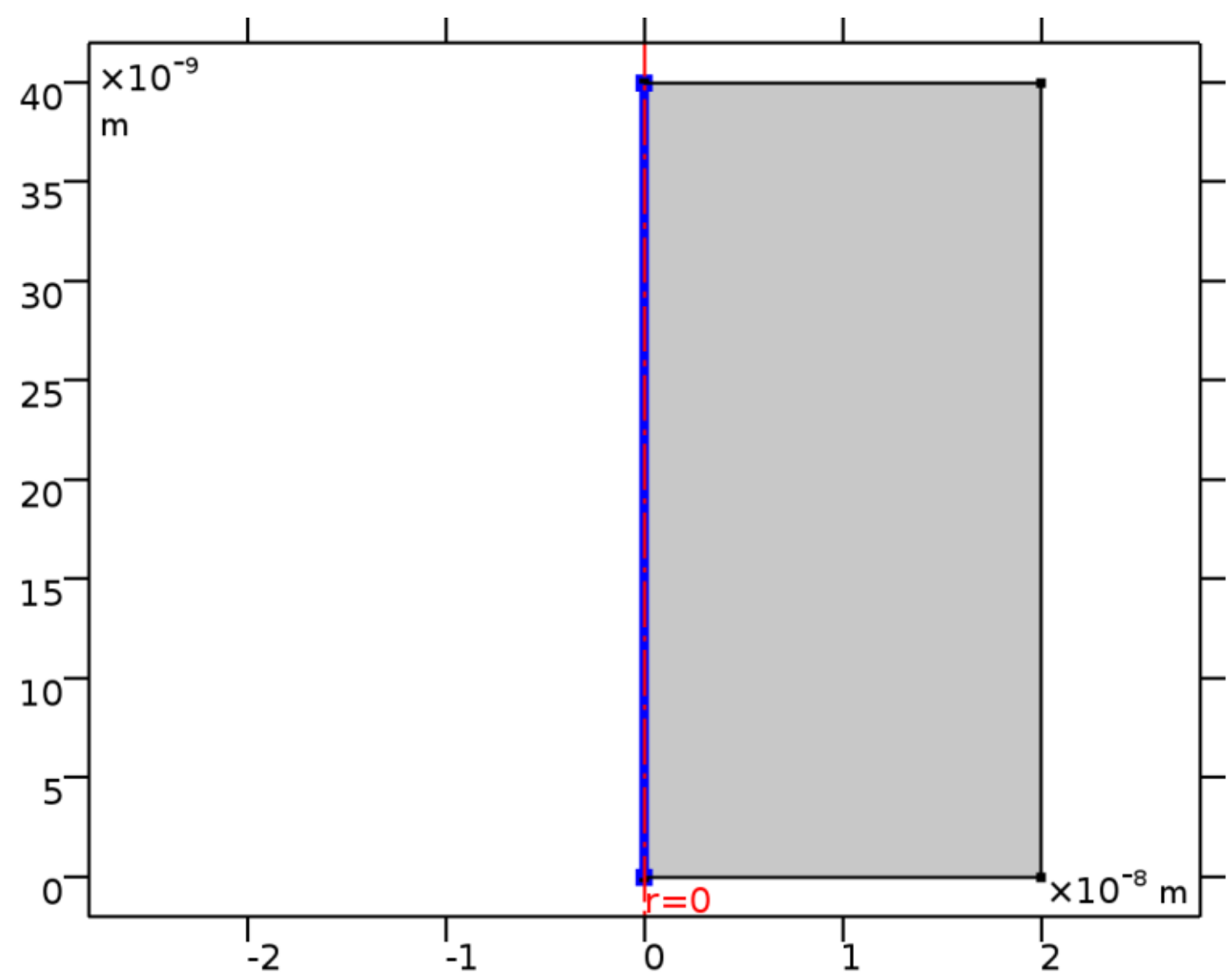

Axial Symmetry 1

SELECTION

Geometric entity level	Boundary
Selection	Boundary 1

USED PRODUCTS

COMSOL Multiphysics

2.3.5 No Flux 1

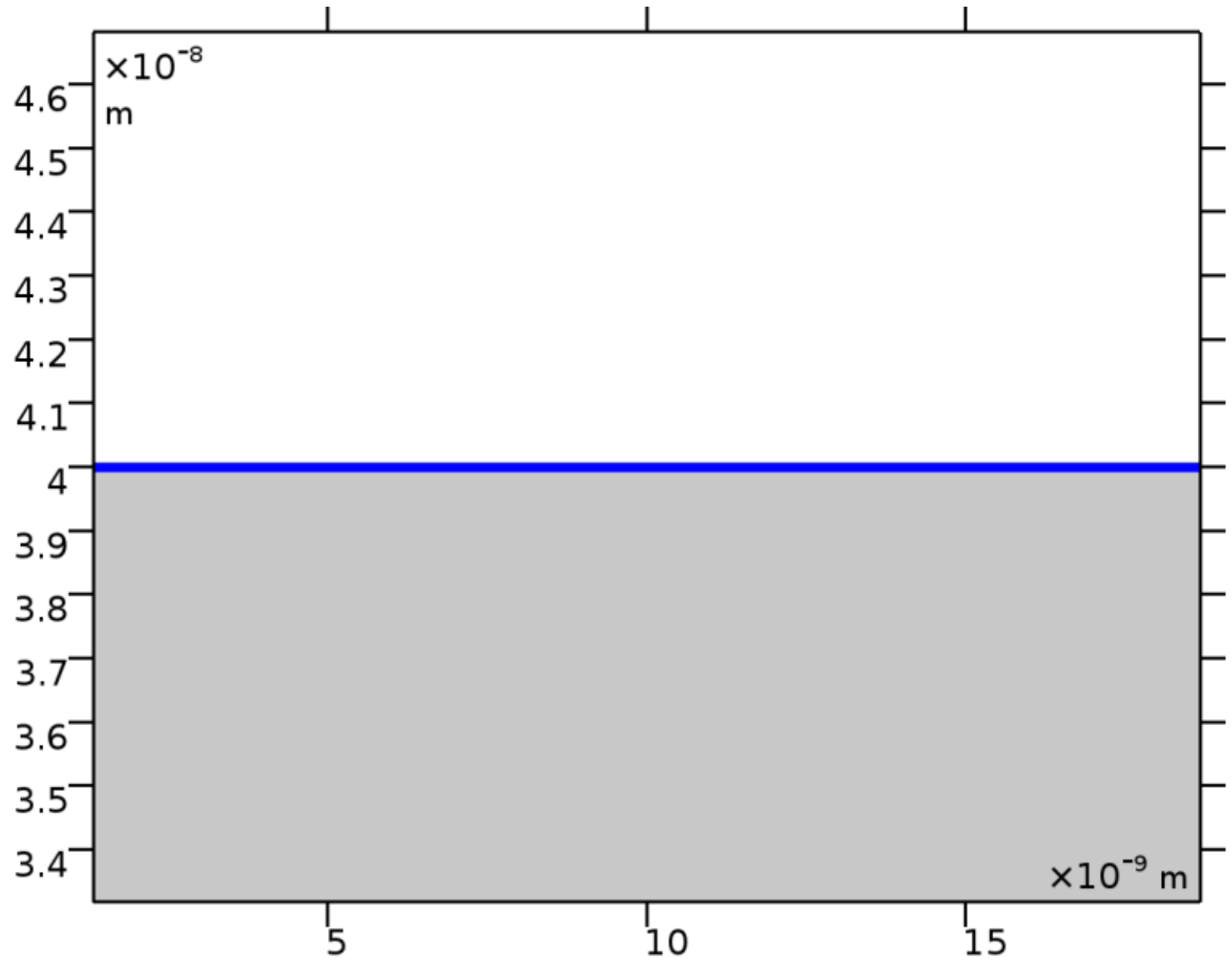

No Flux 1

SELECTION

Geometric entity level	Boundary
Selection	Boundary 4

EQUATIONS

$$-\mathbf{n} \cdot \mathbf{N}_i = 0$$

USED PRODUCTS

COMSOL Multiphysics

2.3.6 Initial Values 1

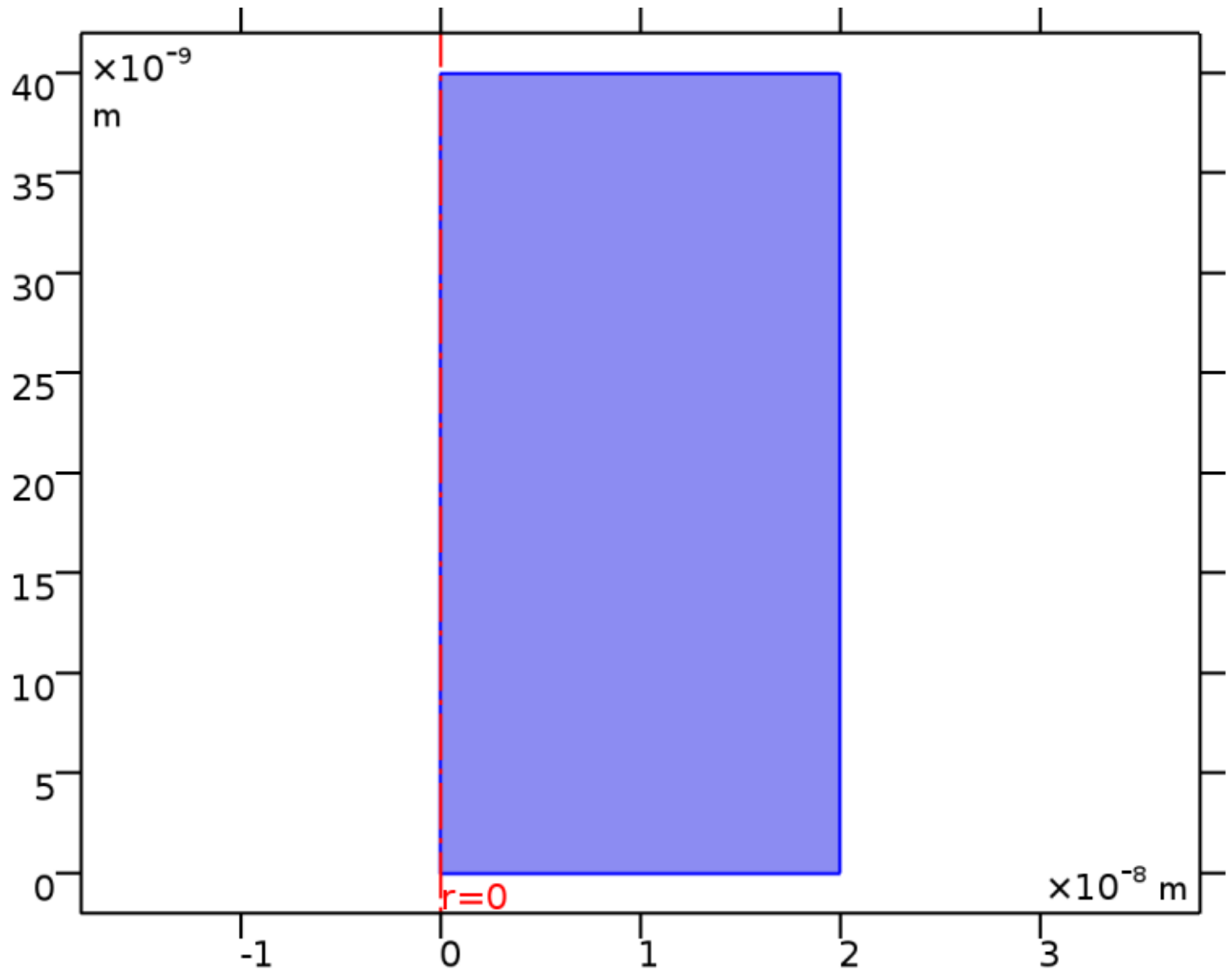

Initial Values 1

SELECTION

Geometric entity level	Domain
Selection	Domain 1

Initial values

SETTINGS

**Description**	**Value**
Concentration	{c1ini, 0}

USED PRODUCTS

COMSOL Multiphysics

2.3.7 Flux 1

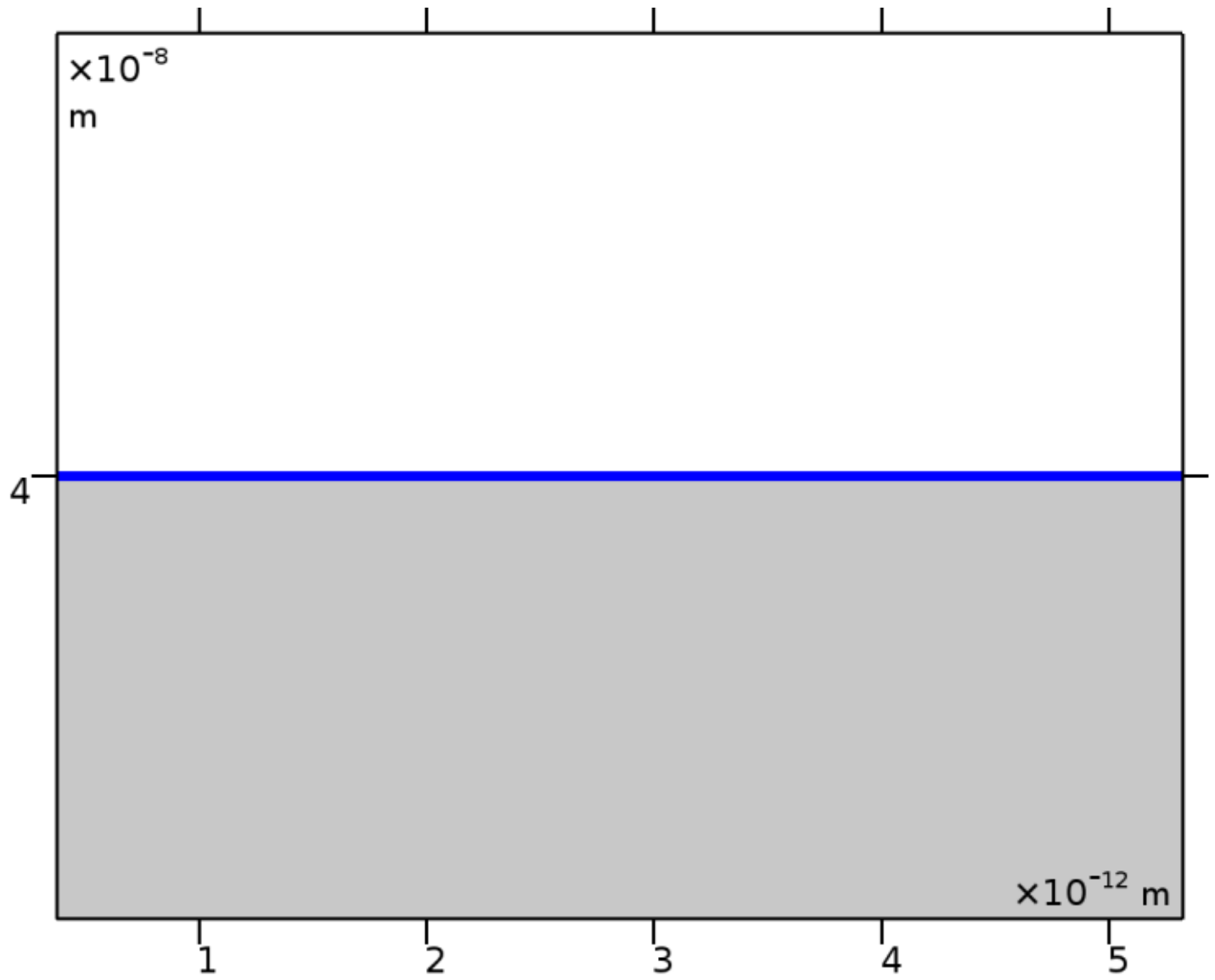

Flux 1

SELECTION

Geometric entity level	Boundary
Selection	Boundary 3

EQUATIONS

$$-\mathbf{n}\cdot\mathbf{N}_i = N_{0,i}$$

Inward flux

SETTINGS

Description	Value
Flux type	General inward flux
Species c1	On
Species c2	On
	{-kft*c1 + kbt*c2, -(-kft*c1 + kbt*c2)}

USED PRODUCTS

COMSOL Multiphysics

Weak expressions

Weak expression	Integration order	Integration frame	Selection
2*(-kft*c1+kbt*c2)*test(c1)*pi*r	2	Material	Boundary 3
-2*(-kft*c1+kbt*c2)*test(c2)*pi*r	2	Material	Boundary 3

2.3.8 Concentration 1

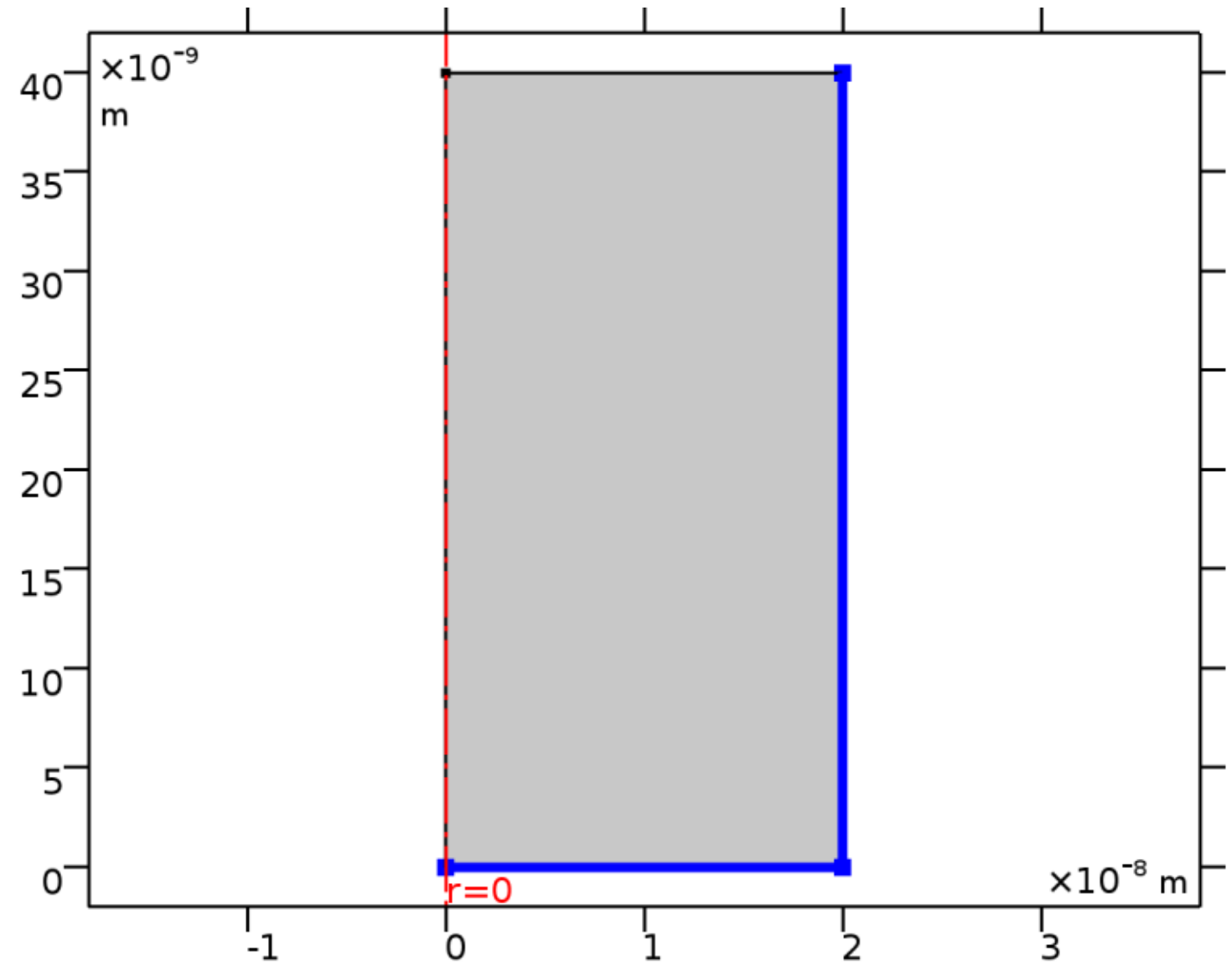

Concentration 1

SELECTION

Geometric entity level	Boundary
Selection	Boundaries 2, 5

EQUATIONS

$$c_i = c_{0,i}$$

Concentration

SETTINGS

**Description**	**Value**
Species c1	On
Species c2	On
Concentration	{c1ini, 0}

USED PRODUCTS

COMSOL Multiphysics

Variables

**Name**	**Expression**	**Unit**	**Description**	**Selection**
chds.c0_c1	c1ini	mol/m³	Concentration	Boundaries 2, 5
chds.c0_c2	0	mol/m³	Concentration	Boundaries 2, 5

Constraints

Constraint	Constraint force	Shape function	Selection	Details
-c1+chds.c0_c1	test(-c1+chds.c0_c1)	Lagrange (Linear)	Boundaries 2, 5	Elemental
-c2+chds.c0_c2	test(-c2+chds.c0_c2)	Lagrange (Linear)	Boundaries 2, 5	Elemental

2.4 MESH 1

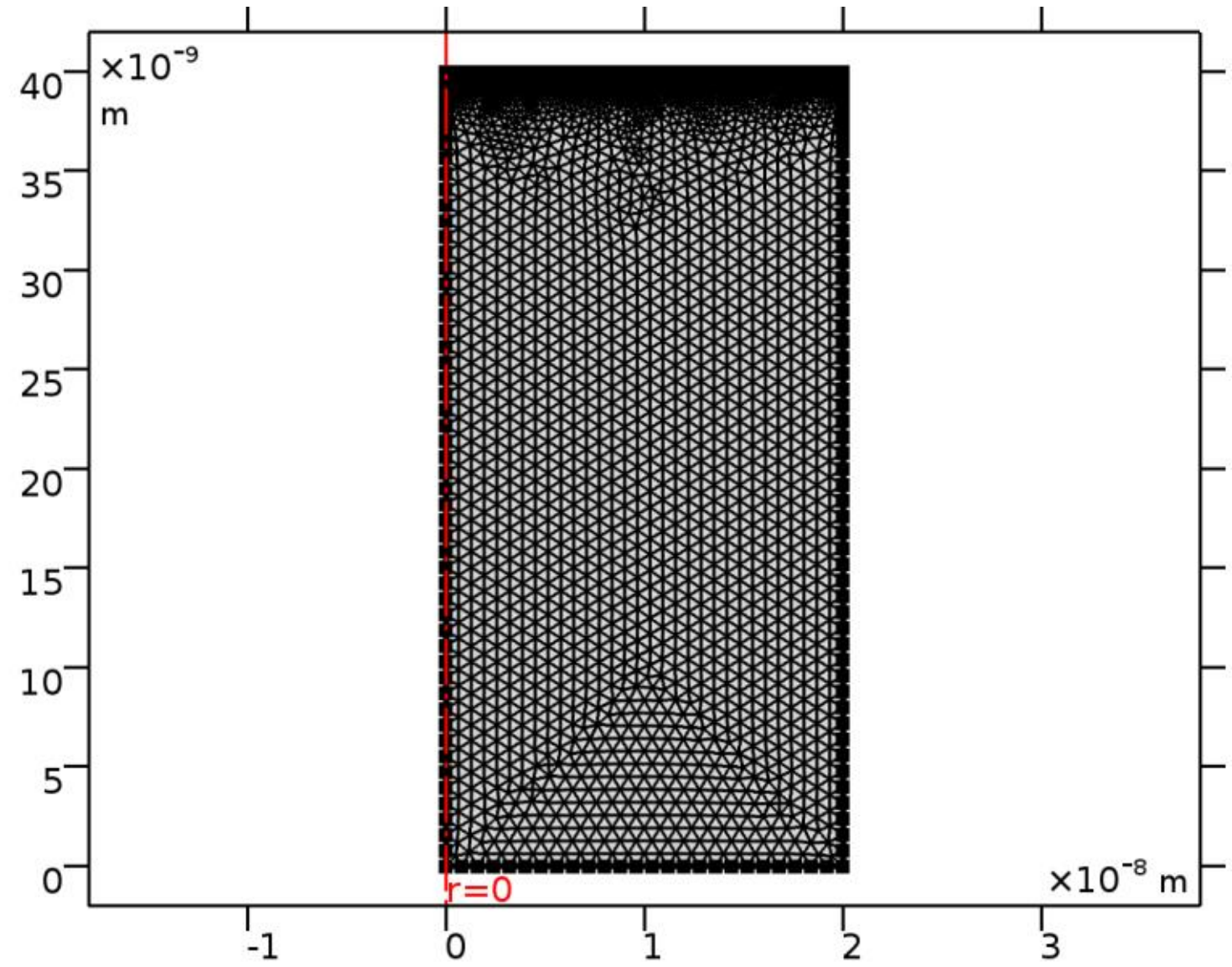

Mesh 1

2.4.1 Size (size)

SETTINGS

Description	Value
Maximum element size	8.0E-10
Minimum element size	3.0E-12
Curvature factor	0.25
Maximum element growth rate	1.2
Predefined size	Extra fine

2.4.2 Size 1 (size1)

SELECTION

Geometric entity level	Boundary
Selection	Boundary 3

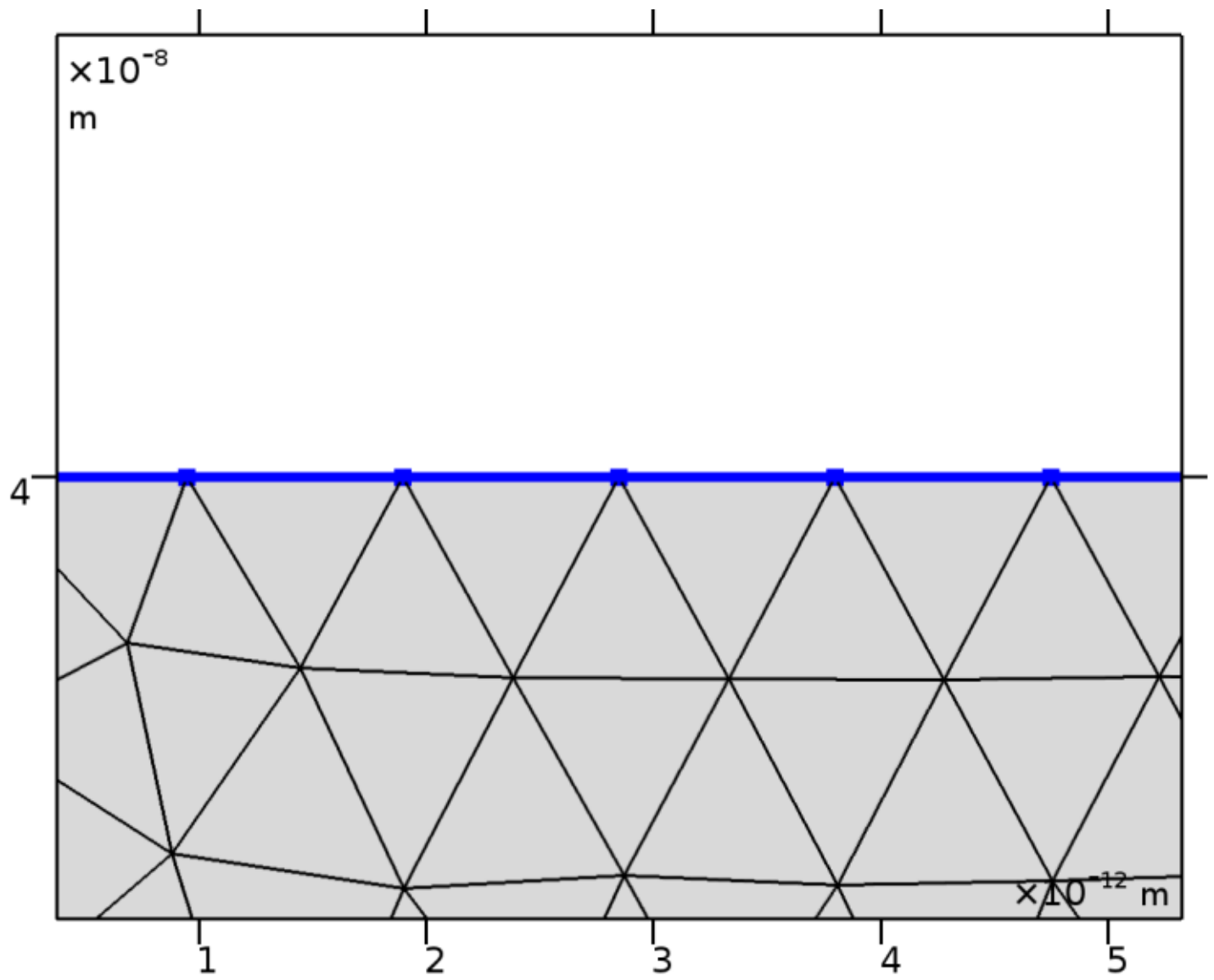

Size 1

SETTINGS

Description	Value
Maximum element size	1E-12
Minimum element size	0.01E-12
Curvature factor	0.3
Curvature factor	Off
Resolution of narrow regions	Off
Maximum element growth rate	1.3
Maximum element growth rate	Off
Custom element size	Custom

2.4.3 Size 2 (size2)

SELECTION

Geometric entity level	Boundary
Selection	Boundary 4

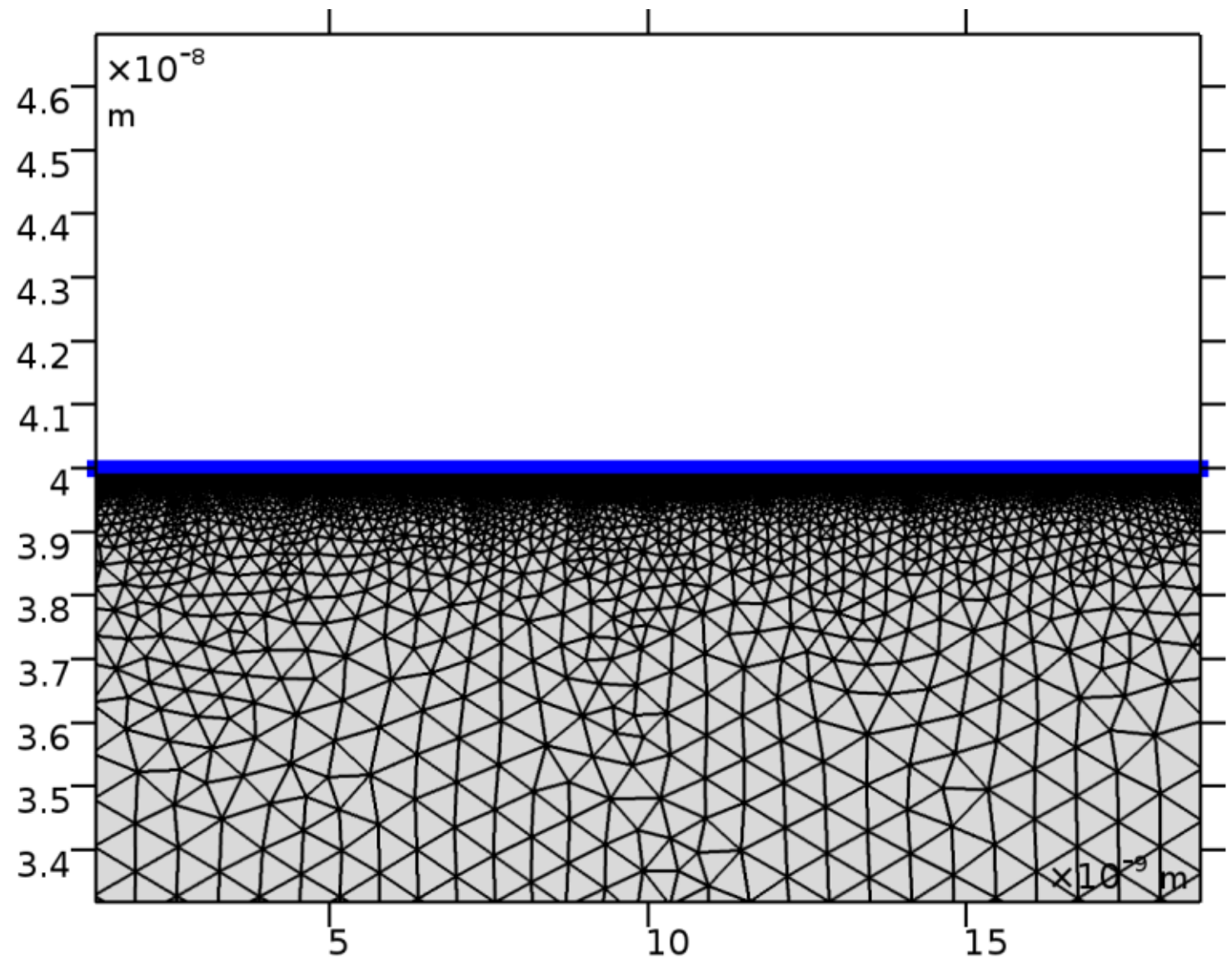

Size 2

SETTINGS

Description	Value
Maximum element size	0.003E-9
Minimum element size	0.001E-9
Curvature factor	0.3
Curvature factor	Off
Resolution of narrow regions	Off
Maximum element growth rate	1.3
Maximum element growth rate	Off
Custom element size	Custom

2.4.4 Free Triangular 1 (ftri1)

SELECTION

Geometric entity level	Domain
Selection	Remaining

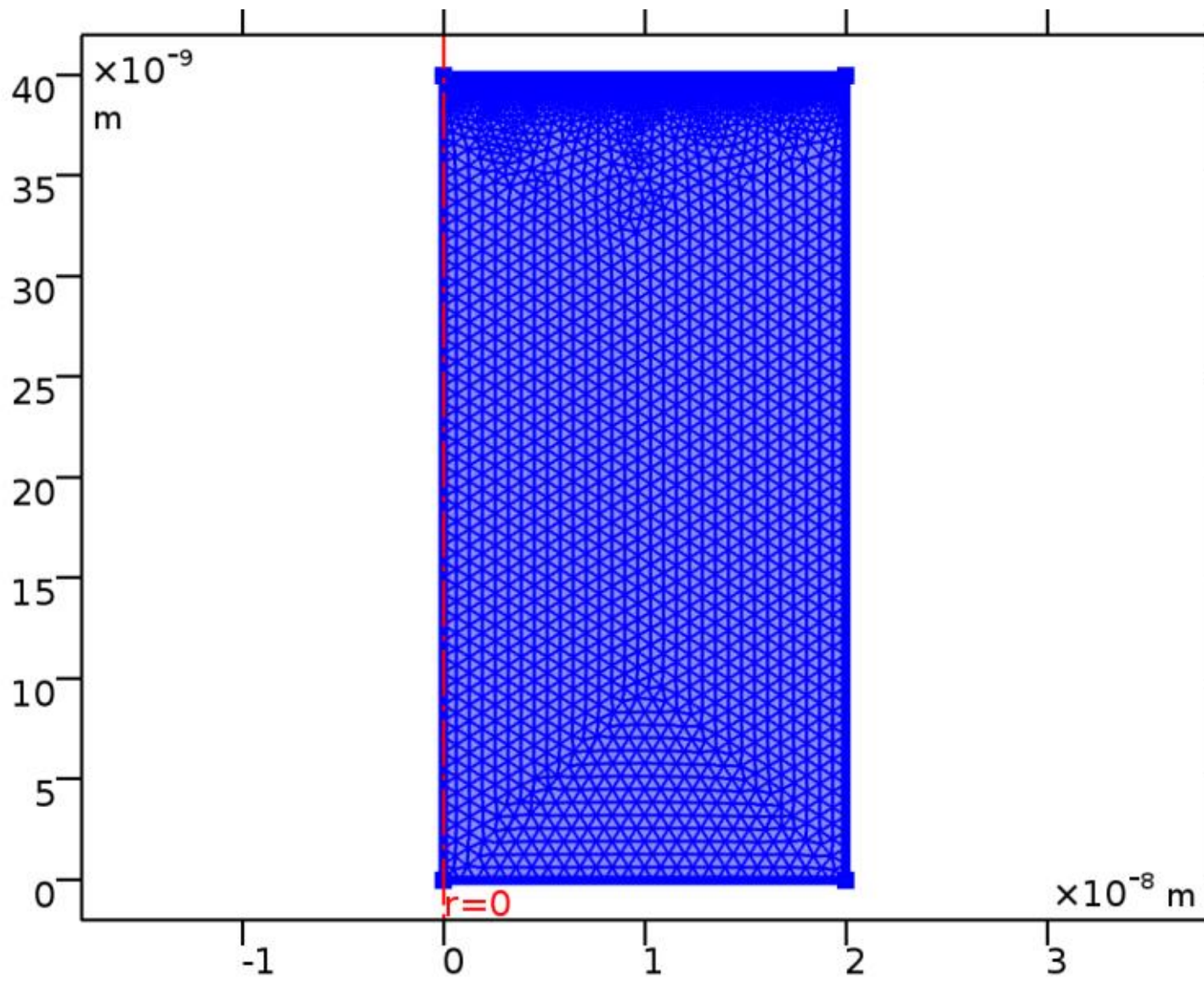

Free Triangular 1

3 Study 1

COMPUTATION INFORMATION

Computation time	1 min 5 s
CPU	Intel64 Family 6 Model 63 Stepping 2, 6 cores
Operating system	Windows 7

3.1 TIME DEPENDENT

Times	Unit
range(0,0.1,40)	s

PHYSICS AND VARIABLES SELECTION

Physics interface	Discretization
Transport of Diluted Species (chds)	physics

MESH SELECTION

Geometry	Mesh
Geometry 1 (geom1)	mesh1

3.2 SOLVER CONFIGURATIONS

3.2.1 Solver 1

Compile Equations: Time Dependent (st1)

STUDY AND STEP

Description	Value
Use study	Study 1
Use study step	Time Dependent

Dependent Variables 1 (v1)

GENERAL

Description	Value
Defined by study step	Time Dependent

SETTINGS

Constant name	Initial value source
t	range(0,0.1,40)
timestep	0.04[s]

Concentration (mod1.c2) (mod1_c2)

GENERAL

Description	Value
Field components	mod1.c2

Concentration (mod1.c1) (mod1_c1)

GENERAL

Description	Value
Field components	mod1.c1

Time-Dependent Solver 1 (t1)

GENERAL

Description	Value
Defined by study step	Time Dependent
Times	{0, 0.1, 0.2, 0.30000000000000004, 0.4, 0.5, 0.6000000000000001, 0.7000000000000001, 0.8, 0.9, 1, 1.1, 1.2000000000000002, 1.3, 1.4000000000000001, 1.5, 1.6, 1.7000000000000002, 1.8, 1.9000000000000001, 2, 2.1, 2.2, 2.3000000000000003, 2.4000000000000004, 2.5, 2.6, 2.7, 2.8000000000000003, 2.9000000000000004, 3, 3.1, 3.2, 3.3000000000000003, 3.4000000000000004, 3.5, 3.6, 3.7, 3.8000000000000003, 3.9000000000000004, 4, 4.1000000000000005, 4.2, 4.3, 4.4, 4.5, 4.6000000000000005, 4.7, 4.800000000000001, 4.9, 5, 5.1000000000000005, 5.2, 5.300000000000001, 5.4, 5.5, 5.6000000000000005, 5.7, 5.800000000000001, 5.9, 6, 6.1000000000000005, 6.2, 6.300000000000001, 6.4, 6.5, 6.6000000000000005, 6.7, 6.800000000000001, 6.9, 7, 7.1000000000000005, 7.2, 7.300000000000001, 7.4, 7.5, 7.6000000000000005, 7.7, 7.800000000000001, 7.9, 8, 8.1, 8.200000000000001, 8.3, 8.4, 8.5, 8.6, 8.700000000000001, 8.8, 8.9, 9, 9.1, 9.200000000000001, 9.3, 9.4, 9.5, 9.600000000000001, 9.700000000000001, 9.8, 9.9, 10, 10.100000000000001, 10.200000000000001, 10.3, 10.4, 10.5, 10.600000000000001, 10.700000000000001, 10.8, 10.9, 11, 11.100000000000001, 11.200000000000001, 11.3, 11.4, 11.5, 11.600000000000001, 11.700000000000001, 11.8, 11.9, 12, 12.100000000000001, 12.200000000000001, 12.3, 12.4, 12.5, 12.600000000000001, 12.700000000000001, 12.8, 12.9, 13, 13.100000000000001, 13.200000000000001, 13.3, 13.4, 13.5, 13.600000000000001, 13.700000000000001, 13.8, 13.9, 14, 14.100000000000001, 14.200000000000001, 14.3, 14.4, 14.5, 14.600000000000001, 14.700000000000001, 14.8, 14.9, 15, 15.100000000000001, 15.200000000000001, 15.3, 15.4, 15.5, 15.600000000000001, 15.700000000000001, 15.8, 15.9, 16, 16.1, 16.2, 16.3, 16.400000000000002, 16.5, 16.6, 16.7, 16.8, 16.900000000000002, 17,

Description	Value
	17.1, 17.2, 17.3, 17.400000000000002, 17.5, 17.6, 17.7, 17.8, 17.900000000000002, 18, 18.1, 18.2, 18.3, 18.400000000000002, 18.5, 18.6, 18.7, 18.8, 18.900000000000002, 19, 19.1, 19.200000000000003, 19.3, 19.400000000000002, 19.5, 19.6, 19.700000000000003, 19.8, 19.900000000000002, 20, 20.1, 20.200000000000003, 20.3, 20.400000000000002, 20.5, 20.6, 20.700000000000003, 20.8, 20.900000000000002, 21, 21.1, 21.200000000000003, 21.3, 21.400000000000002, 21.5, 21.6, 21.700000000000003, 21.8, 21.900000000000002, 22, 22.1, 22.200000000000003, 22.3, 22.400000000000002, 22.5, 22.6, 22.700000000000003, 22.8, 22.900000000000002, 23, 23.1, 23.200000000000003, 23.3, 23.400000000000002, 23.5, 23.6, 23.700000000000003, 23.8, 23.900000000000002, 24, 24.1, 24.200000000000003, 24.3, 24.400000000000002, 24.5, 24.6, 24.700000000000003, 24.8, 24.900000000000002, 25, 25.1, 25.200000000000003, 25.3, 25.400000000000002, 25.5, 25.6, 25.700000000000003, 25.8, 25.900000000000002, 26, 26.1, 26.200000000000003, 26.3, 26.400000000000002, 26.5, 26.6, 26.700000000000003, 26.8, 26.900000000000002, 27, 27.1, 27.200000000000003, 27.3, 27.400000000000002, 27.5, 27.6, 27.700000000000003, 27.8, 27.900000000000002, 28, 28.1, 28.200000000000003, 28.3, 28.400000000000002, 28.5, 28.6, 28.700000000000003, 28.8, 28.900000000000002, 29, 29.1, 29.200000000000003, 29.3, 29.400000000000002, 29.5, 29.6, 29.700000000000003, 29.8, 29.900000000000002, 30, 30.1, 30.200000000000003, 30.3, 30.400000000000002, 30.5, 30.6, 30.700000000000003, 30.8, 30.900000000000002, 31, 31.1, 31.200000000000003, 31.3, 31.400000000000002, 31.5, 31.6, 31.700000000000003, 31.8, 31.900000000000002, 32, 32.1, 32.2, 32.300000000000004, 32.4, 32.5, 32.6, 32.7, 32.800000000000004, 32.9, 33, 33.1, 33.2, 33.300000000000004, 33.4, 33.5, 33.6, 33.7, 33.800000000000004, 33.9, 34, 34.1, 34.2, 34.300000000000004, 34.4, 34.5, 34.6, 34.7, 34.800000000000004, 34.9, 35, 35.1, 35.2, 35.300000000000004, 35.4, 35.5, 35.6, 35.7, 35.800000000000004, 35.9, 36, 36.1, 36.2, 36.300000000000004, 36.4, 36.5, 36.6, 36.7, 36.800000000000004, 36.9, 37, 37.1, 37.2, 37.300000000000004, 37.4, 37.5, 37.6, 37.7, 37.800000000000004, 37.9, 38, 38.1, 38.2, 38.300000000000004, 38.400000000000006, 38.5, 38.6, 38.7, 38.800000000000004, 38.900000000000006, 39, 39.1, 39.2, 39.300000000000004, 39.400000000000006, 39.5, 39.6, 39.7, 39.800000000000004, 39.900000000000006, 40}

ABSOLUTE TOLERANCE

Description	Value
Tolerance method	Manual
Absolute tolerance	0.0010

SETTINGS: CONCENTRATION (MOD1.C2)

Description	Value
Tolerance method	Manual

SETTINGS: CONCENTRATION (MOD1.C1)

Description	Value
Tolerance method	Manual

TIME STEPPING

Description	Value
Initial step	0.0010
Maximum BDF order	2
Fraction of initial step for Backward Euler	0.0010

Fully Coupled 1 (fc1)

GENERAL

Description	Value
Linear solver	Direct 1

METHOD AND TERMINATION

Description	Value
Damping factor	0.9
Jacobian update	Once per time step
Maximum number of iterations	8

Direct 1 (d1)

GENERAL

Description	Value
Solver	PARDISO
Multithreaded forward and backward solve	Off
Out-of-core	Off

4 Results

4.1 DATA SETS

4.1.1 Solution 1

SOLUTION

Description	Value
Solution	Solver 1
Component	Save Point Geometry 1

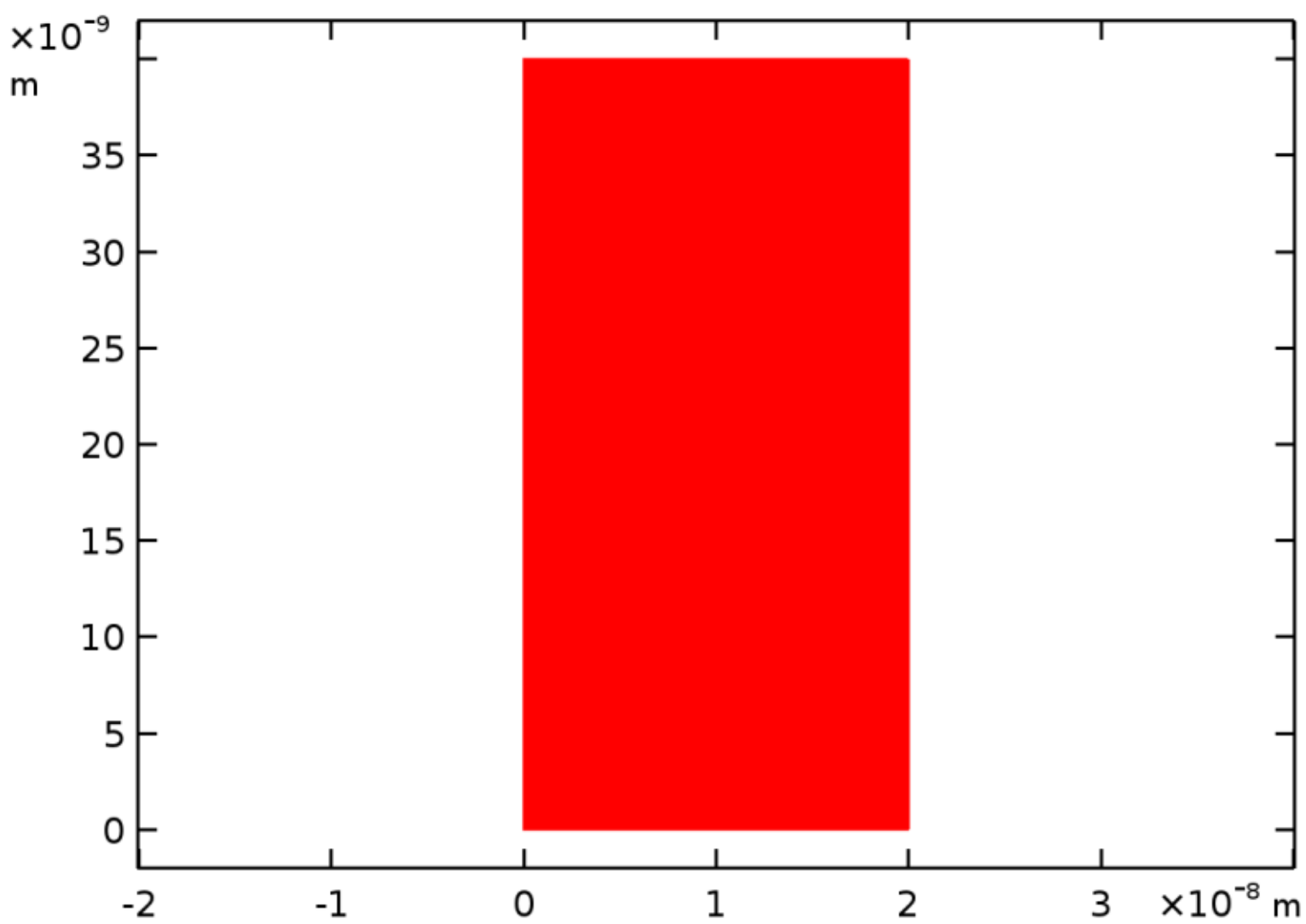

Data set: Solution 1

4.1.2 Revolution 2D 1

DATA

Description	Value
Data set	Solution 1

AXIS DATA

Description	Value
Axis entry method	Two points
Points	{{0, 0}, {0, 1}}

REVOLUTION LAYERS

Description	Value
Start angle	-90

Description	Value
Revolution angle	225

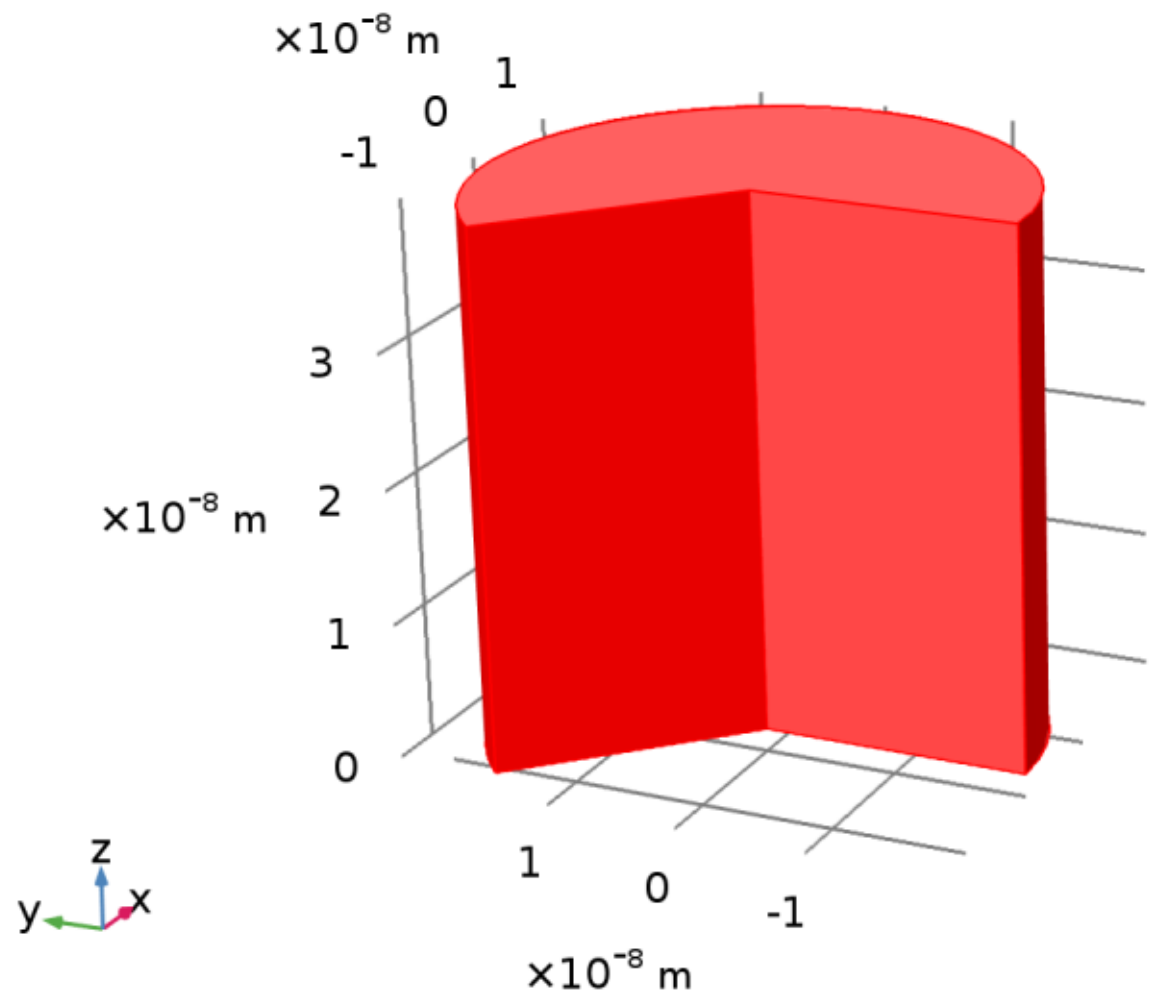

Data set: Revolution 2D 1

4.2 PLOT GROUPS

4.2.1 Concentration (chds)

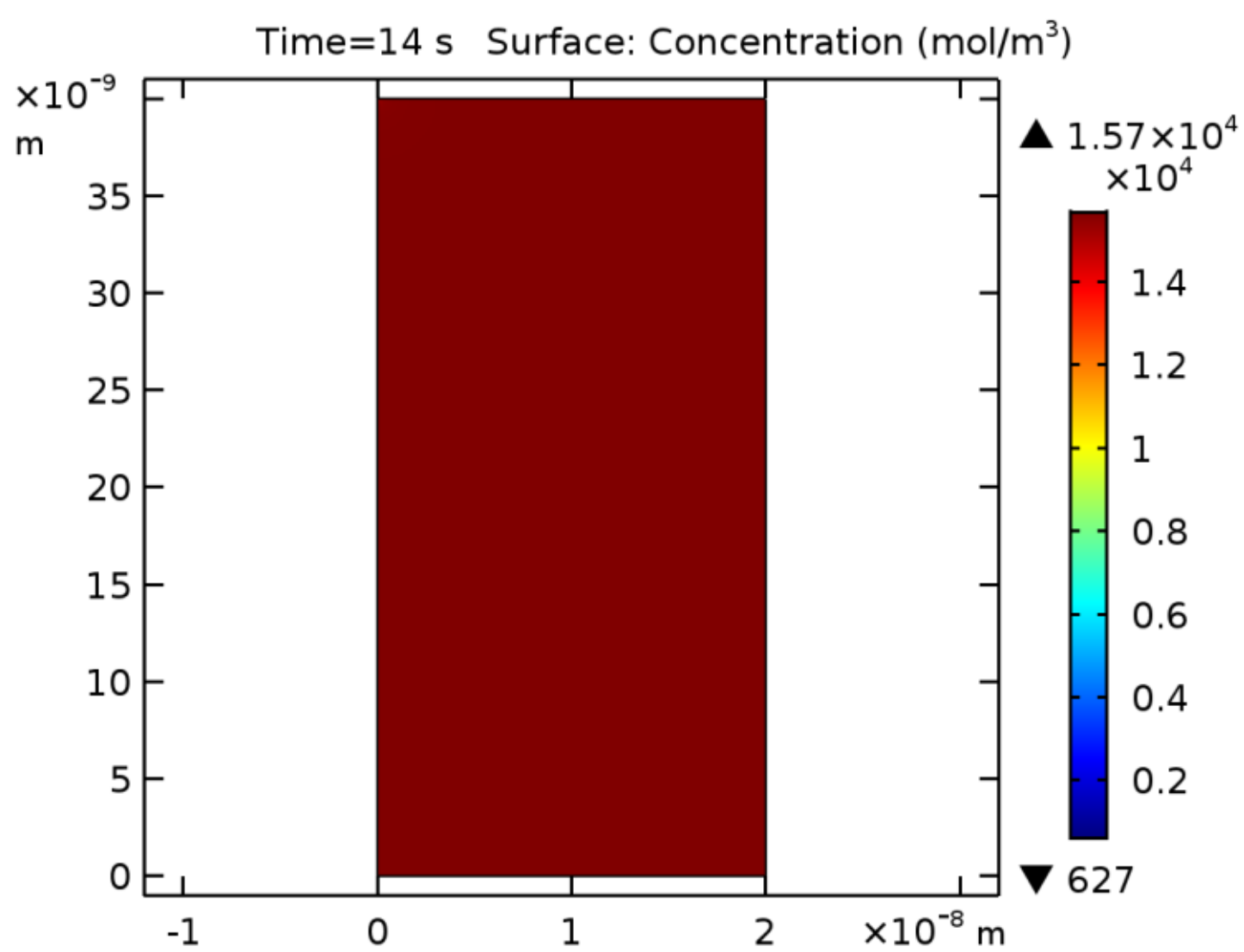

Time=14 s Surface: Concentration (mol/m^3)

4.2.2 Concentration, 3D (chds)

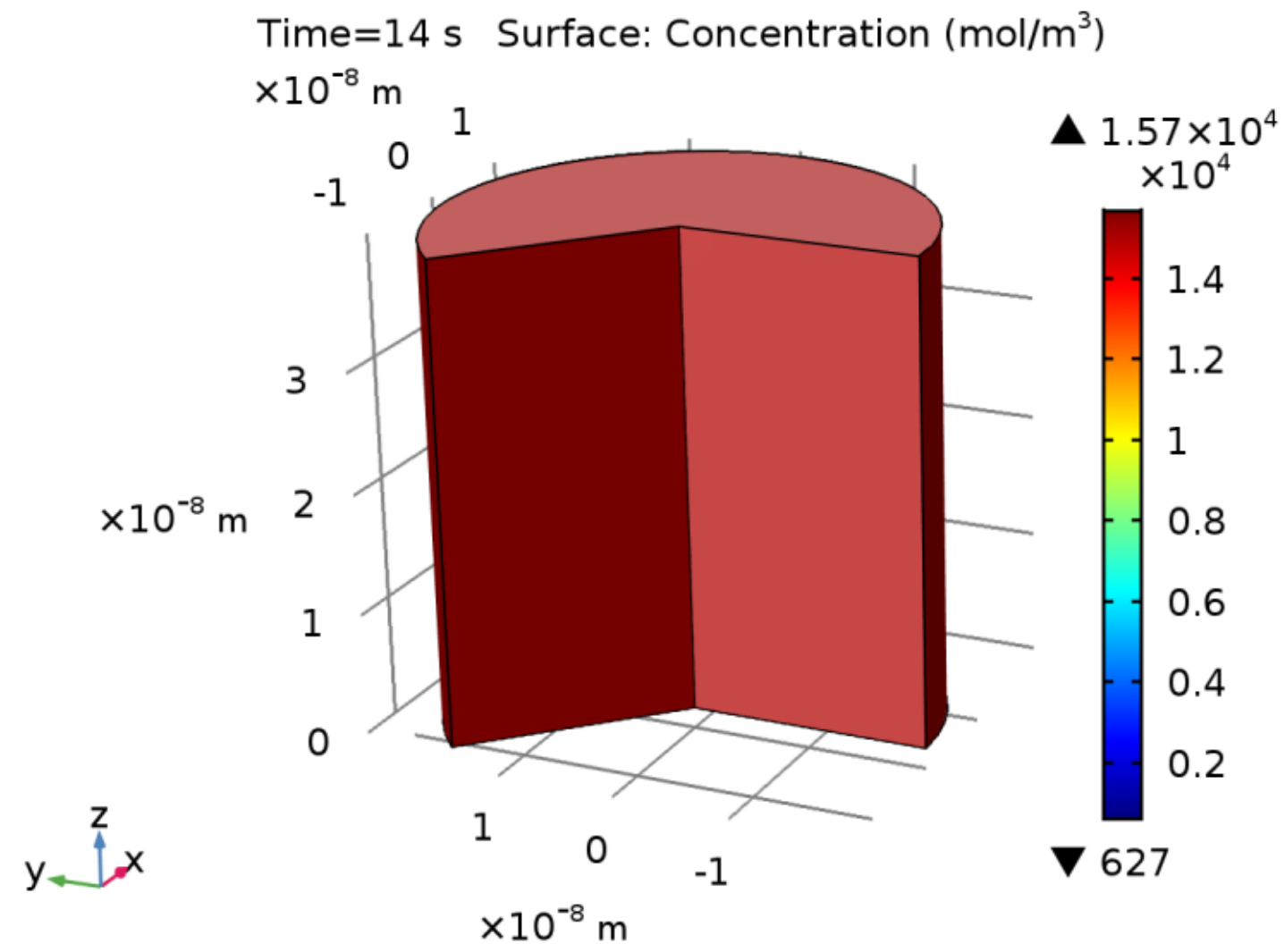

Time=14 s Surface: Concentration (mol/m^3)

4.2.3 1D Plot Group 3

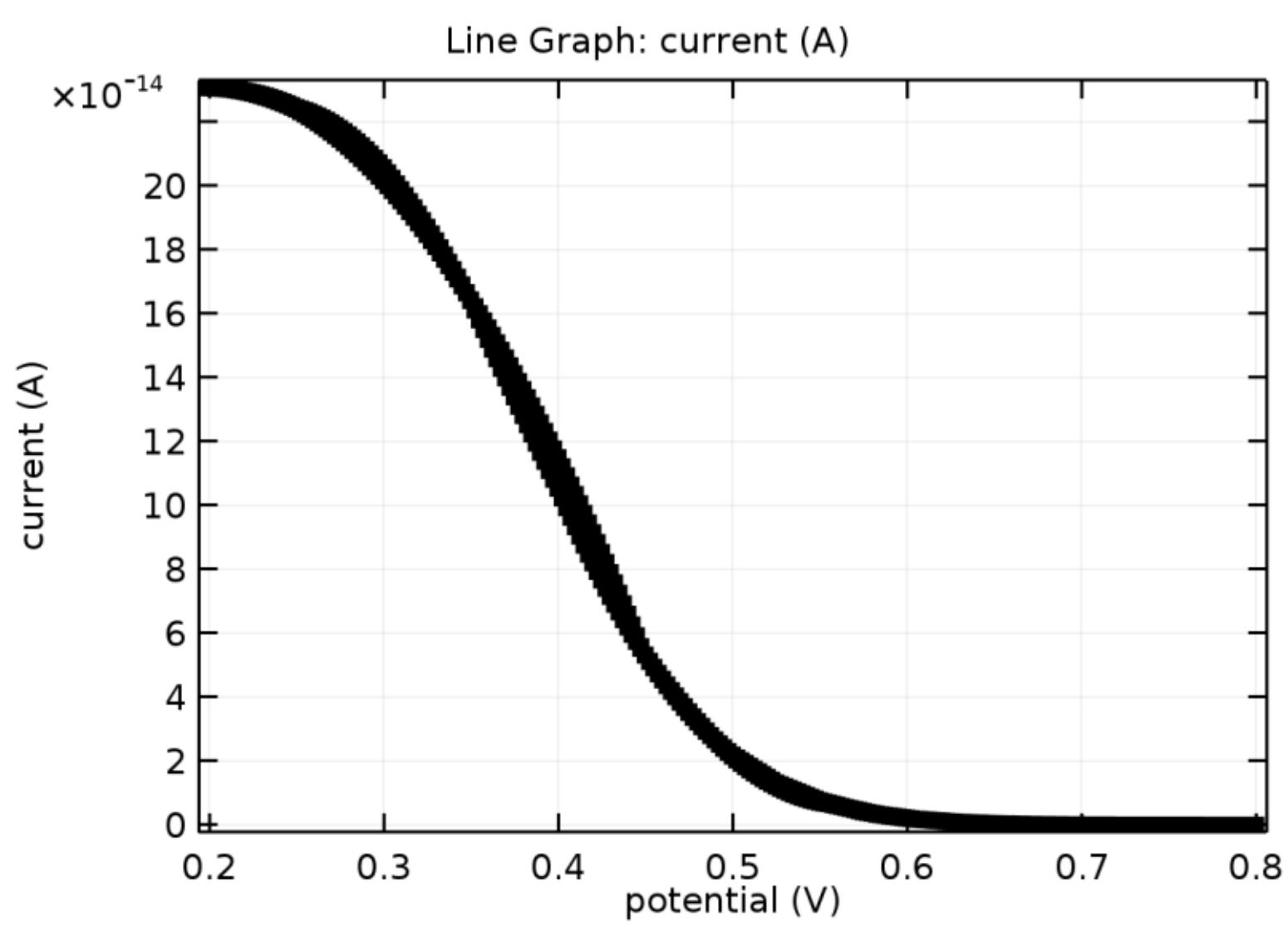

Line Graph: current (A)
